# Human hair–bearing skin organoids reveal cell type–specific susceptibility and innate immune responses to herpes simplex virus 1

**DOI:** 10.1126/sciadv.aef5563

**Published:** 2026-10-30

**Authors:** Emanuel Wyler, Silvia Albertini, Caroline C. Friedel, Artür Manukyan, Annalena Winterfeldt, Nina Bracker, Jiabin Huang, Susanne Krasemann, Izabela Plumbom, Janine Altmüller, Thomas Conrad, Rudolph Reimer, Carola Schneider, Helena Radbruch, Wali Hafezi, Arne Hansen, Adam Grundhoff, Markus Landthaler, Nicole Fischer, Manja Czech-Sioli

**Affiliations:** ^1^ Max Delbrück Center for Molecular Medicine in the Helmholtz Association (MDC), Berlin Institute for Medical Systems Biology, Berlin, Germany.; ^2^ Institute for Molecular Virology and Tumorvirology, University Medical Center Hamburg-Eppendorf, Hamburg, Germany.; ^3^ Institute for Informatics, Ludwig-Maximilians-Universität München, Munich, Germany.; ^4^ Institute of Medical Microbiology, Virology and Hygiene, University Medical Center Hamburg-Eppendorf, Hamburg, Germany.; ^5^ Institute of Neuropathology, University Medical Center Hamburg-Eppendorf, Hamburg, Germany.; ^6^ Genomics Technology Platform, Max Delbrück Center for Molecular Medicine in the Helmholtz Association, Berlin, Germany.; ^7^ Core Facility Genomics, Berlin Institute of Health at Charité - Universitätsmedizin Berlin, Berlin, Germany.; ^8^ Technology Platform Microscopy and Image Analysis/Electron Microscopy, Leibniz Institute of Virology, Hamburg, Germany.; ^9^ Department of Neuropathology, Charité - Universitätsmedizin Berlin, Berlin, Germany.; ^10^ Institute of Virology/Clinical Virology, University Hospital Muenster, Muenster, Germany.; ^11^ Institute of Experimental Pharmacology and Toxicology, University Medical Center Hamburg-Eppendorf, Hamburg, Germany.; ^12^ Department of Viral Genomics, Leibniz Institute of Virology, Hamburg, Germany.; ^13^ German Center for Infection Research, Partner Site Hamburg-Borstel-Lübeck-Riems, Hamburg, Germany.; ^14^ Institut für Biologie, Humboldt-Universität zu Berlin, Berlin, Germany.

## Abstract

Epithelial surfaces are the primary sites of lytic herpes simplex virus 1 (HSV-1) infection following initial exposure or reactivation from latency. To model this complex environment, we used human hair–bearing skin organoids and combined single-cell spatial transcriptomics with imaging to map viral spread and host responses across skin architecture. Our data suggest delayed progression of the HSV-1 life cycle in epidermal keratinocytes and hair follicle cells compared to the dermis. Infection triggered spatially organized host responses, including induction of IER3 in papillary fibroblasts. In basal keratinocytes, IRF3 was up-regulated in both infected and neighboring uninfected cells, consistent with paracrine activation of antiviral signaling. Inflammatory responses were compartmentalized in a cell type–specific manner, with TNFSF9 (4-1BBL) induced in papillary fibroblasts and tumor necrosis factor–α (TNF-α) induced in keratinocytes. Enzyme-linked immunosorbent assays confirmed secretion of these cytokines and the TNF-α–responsive chemokines CXCL2 and CXCL10. CXCL2 expression was enriched in cells adjacent to TNF-α–expressing cells, highlighting spatial coordination of inflammatory signaling. While interferon-β reduced viral replication, TNF-α and 4-1BBL did not, suggesting that their primary role is to orchestrate immune cell recruitment and activation. These findings establish human hair–bearing skin organoids as a physiologically relevant model for investigating spatial host–HSV-1 interactions.

## INTRODUCTION

Herpes simplex virus 1 (HSV-1) is a highly prevalent human pathogen with a global prevalence of ∼67% in under 50-year-old individuals (*1*). Primary infection typically occurs through mucosal surfaces or micro-lesions in the skin. Initially, HSV-1 productively replicates in the epithelial cells of skin and mucosa, causing local inflammation and tissue damage (*2*). Following replication in epithelial cells, HSV-1 enters the nerve endings of sensory peripheral neurons and undergoes retrograde axonal transport to the cell bodies in trigeminal ganglia where it establishes latency (*3*, *4*). Upon spontaneous reactivation, HSV-1 enters the lytic life cycle and produces infectious viral particles that migrate in an anterograde manner to the epithelial cells of skin and mucosa. Subsequent lytic replication in the epithelium leads to the characteristic herpes lesions that release virus to the environment (*2*).

A variety of different model systems have been used to elucidate the mechanisms of primary infection and reactivation by HSV-1 in the skin. Because of its complexity and the involvement of many different cell types, in vitro modeling by monolayer cell cultures can only recapitulate certain aspects of the viral life cycle. More complex model systems used to study virus susceptibility, spread, and cytopathic effects (CPEs) are animal models (*5*–*8*), ex vivo murine (*8*–*10*) and human skin explants (*8*, *11*, *12*), and epithelial raft cultures (*13*, *14*). While the understanding of HSV-1 pathogenesis in the skin has exceptionally increased using these model systems, many aspects remain elusive. In particular, the susceptibility and contributions of specific structures, like hair follicles (HFs), different fibroblast subpopulations, and low abundancy cell types, to primary infection, latency establishment, and reactivation are not understood.

The field of organoids is constantly evolving and creating potential new model systems for virus research (*15*–*17*). In this study, we used a highly complex skin organoid (SkO) model generated from human induced pluripotent stem cells (hiPSCs) for the analysis of HSV-1 pathogenesis in the skin (*18*, *19*). SkOs contain a stratified epidermis, fat-rich dermis, HFs producing pigmented hair, sebaceous glands, melanocytes, and Merkel cells. The HFs are innervated by sensory neurons that are covered in Schwann cells on their axons while their somata assemble in trigeminal-like clusters together with satellite glial cells. SkOs contain all cell types expected in the fetal skin of the second trimester of development except for sweat glands, vascularization, and immune cells (*18*, *19*). Similar to many other organoids, SkOs show an inside-out morphology with the dermis being exterior and exposed to the culture medium and the epidermis being interior with hair from HFs growing to the inside of the organoid. In this study, SkOs were infected with HSV-1 from the dermal side, leading to viral spread from the dermis to the epidermis. Given the direction of inoculation and the observed infection dynamics, this model most closely resembles a primary HSV-1 infection following deep dermal wounding. We analyzed the susceptibility of different cell types to HSV-1 infection using microscopy and spatial transcriptomics and found a restricted HSV-1 infection in keratinocytes and in HF cells of both dermal and epidermal origin. Furthermore, we found the host factors IER3 and IRF3 up-regulated in a spatiotemporal manner in response to the infection. We found a spatially coordinated inflammatory response by expression of tumor necrosis factor (TNF; coding for TNF-α) and TNFSF9 (4-1BB ligand) leading to downstream activation of TNF target genes. Overall, our study shows how complex human organoid infection models, combined with high-resolution spatial omics methods, enable the investigation of cellular responses and the progression of infection through complex three-dimensional tissues.

## RESULTS

### Infection of SkOs with HSV-1

SkOs were produced according to the protocol by Lee *et al.* (*19*) and their differentiation was verified by bright-field microscopy, immunohistochemistry (IHC), and whole-mount immunofluorescence (WMI) staining (fig. S1, A to D). We observed the expected full complexity at ∼120 days of differentiation with the dermis and epidermis in the head structure and cartilage in the tail. The keratinocytes in the epidermis showed a basal layer and differentiation into stratum spinosum and granulosum with terminally differentiated dying keratinocytes accumulating in the head of the SkOs (fig. S1D). HFs showed the expected structures, i.e., dermal papilla, matrix, inner root sheath, and outer root sheath. We detected Merkel cells and innervation of HFs with assembly of neuronal somata in the organoid tail (fig. S1, B to D).

SkOs at 120 days of differentiation were infected with HSV-1 strain 17 expressing GFP under the control of a CMV IE promoter. To observe spread of the infection within the tissue from single spots, infection was performed at a low viral dose (800 PFU per SkO). At 2 days postinfection (dpi), GFP expression was visible by fluorescence microscopy at spots on the surface of the organoid (Fig. 1A). In IHC staining, the first layers of fibroblasts in the dermis showed expression of HSV-1 glycoprotein D (gD) and GFP as well as indications of a CPE characterized by condensed nuclei and loss of tissue integrity. At 2 dpi, infection spread solely among cells of the dermis with the exclusion of HFs (Fig. 1B and fig. S2A). At 4 dpi, GFP fluorescence increased and covered the entire organoid (Fig. 1A). IHC staining for gD and GFP showed that the infection reached the inner structures of the organoid including the HFs and keratinocytes of the basal layer and the stratum spinosum. Terminally differentiated keratinocytes showed no signs of infection. Similarly, the different cell layers at the HFs were positive for gD and GFP except for the cornified hair structures (Fig. 1B and fig. S2A). Until 8 dpi, we observed no sign of infection in these structures, as in the cartilage of the organoid tail. At 4 dpi, a CPE could be observed mainly in the dermis. At 6 dpi, infected keratinocytes in the epidermis and the inner cell layers at the HFs started to show a CPE that resulted in a progressive loss of epidermal and HF integrity (Fig. 1B, green arrowheads). To compare kinetics of viral spread through three-dimensional tissue to two-dimensional cell culture, we infected SkOs and NHDF cells side by side with three different MOIs (fig. S2, B and C). Under all conditions, viral DNA levels in the culture supernatant increased rapidly. However, NHDF cells reached peak viral DNA levels as early as 2 dpi, whereas SkOs did not reach comparable levels until 4 dpi. In SkOs, higher infection doses resulted in a greater number of infection foci on the organoid surface and accelerated the accumulation of viral DNA in the supernatant. Nevertheless, the maximum viral DNA levels were comparable across all inocula. Overall, these findings indicate delayed HSV-1 replication kinetics in three-dimensional SkO tissue compared with two-dimensional NHDF cultures (fig. S2, B and C) and that HSV-1, over the course of several days, infects almost all cell types of SkOs with indications of differential spreading in different cell types.

**Fig. 1.**
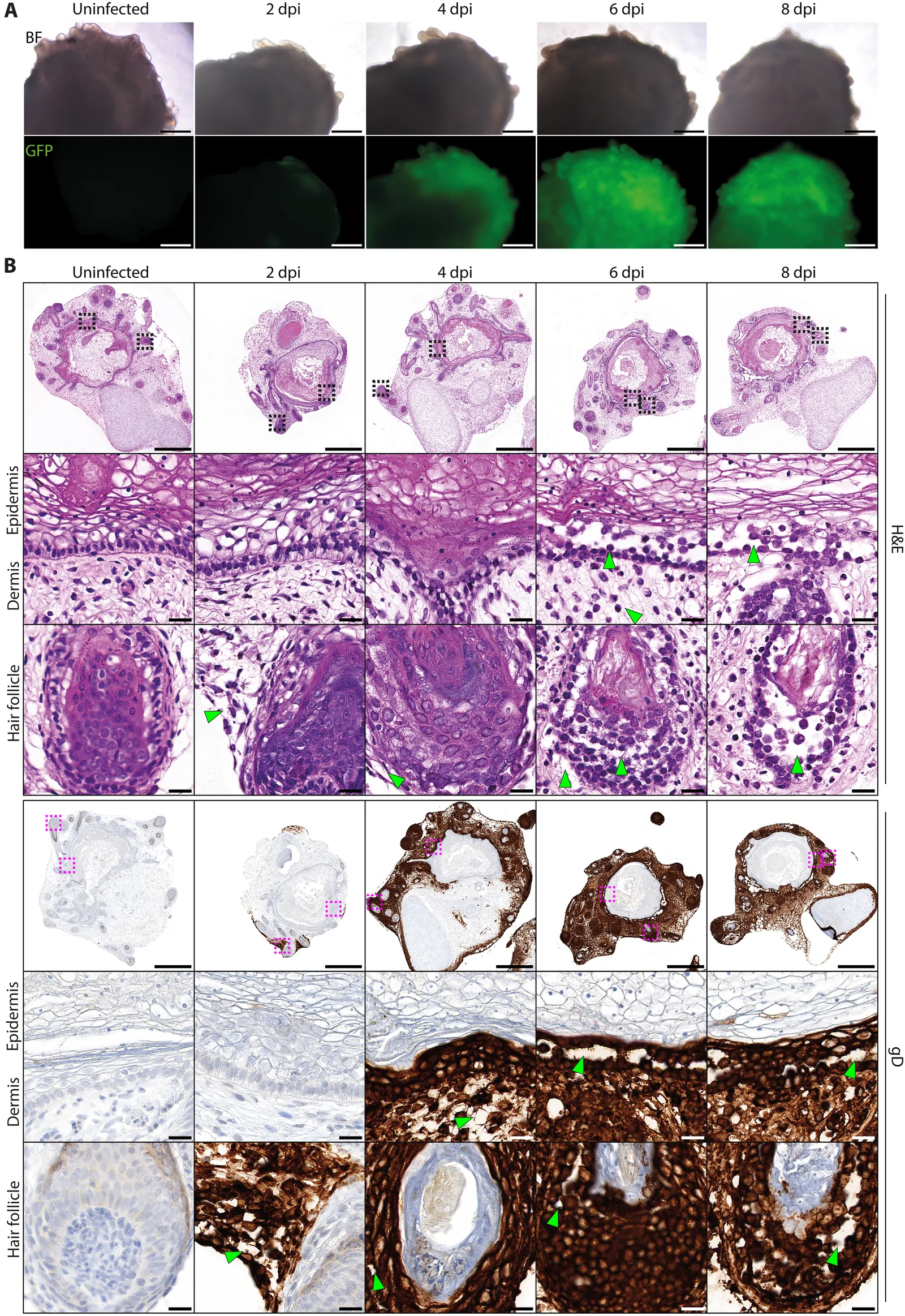
Infection of skin organoids with HSV-1 leads to cytopathic effect (CPE). (**A**) Bright-field (BF) and fluorescence (GFP) images of skin organoids (SkOs) infected with HSV-1 (expressing GFP from a CMV-IE promoter) at 120 days of differentiation. Shown are uninfected and HSV-1–infected SkOs at 2, 4, 6, and 8 days postinfection (dpi). Scale bars, 500 μm. (**B**) IHC staining of SkOs infected with HSV-1 as shown in (A). Boxes in overview images indicate areas of higher magnification shown below. Scale bars, 500 μm (overview images) and 20 μm (zoom-ins). Upper picture of zoom-ins shows epidermis on the top and dermis below. Lower picture of zoom-ins shows hair follicle. Green arrowheads indicate CPE in dermis and epidermis characterized by condensed nuclei and loss of tissue integrity. H&E, hematoxylin and eosin stain; gD, staining against HSV-1 glycoprotein D.

### Bulk transcriptomics of HSV-1–infected SkOs

To analyze the dynamics of viral gene expression and the host response to the viral infection, we performed bulk RNA sequencing (RNA-seq) of uninfected and HSV-1–infected SkOs at 2, 4, 6 and 8 dpi. At 2 dpi, viral transcripts accounted for less than 5% of all sequencing reads. This proportion increased markedly to approximately 20% at 4 dpi and subsequently showed only a modest further increase to ∼25% at 6 and 8 dpi (Fig. 2A). Concomitantly, we observed only 20 differentially expressed genes (DEGs) at 2 dpi, whereas the number of DEGs increased to ∼1000 at 4 dpi and ∼5000 at 6 and 8 dpi (Fig. 2B and fig. S3, A and B). At 4 dpi, Gene Ontology (GO) terms associated with inflammation signaling (e.g., NF-κB signaling and response to TNF) were enriched among genes up-regulated relative to uninfected controls (Fig. 2C). By contrast, at 6 and 8 dpi, GO terms related to cellular differentiation (e.g., skin development and molting cycle) were prominent among the down-regulated DEGs. This could reflect the typical HSV-1–induced host cell shutoff (*20*) and the observed CPE (Fig. 1B, green arrowheads). Terminal deoxynucleotidyl transferase dUTP nick end labeling (TUNEL), which identifies DNA fragmentation, together with staining for cleaved caspase-3 detecting apoptosis, confirmed that cell death becomes widespread at 6 dpi but not at 4 dpi (fig. S4). Together, these data indicated that key transcriptional changes occurs within the first 4 dpi, whereas increasing viral dominance and cell death characterized later time points, thereby defining the time window for the following experiments.

**Fig. 2.**
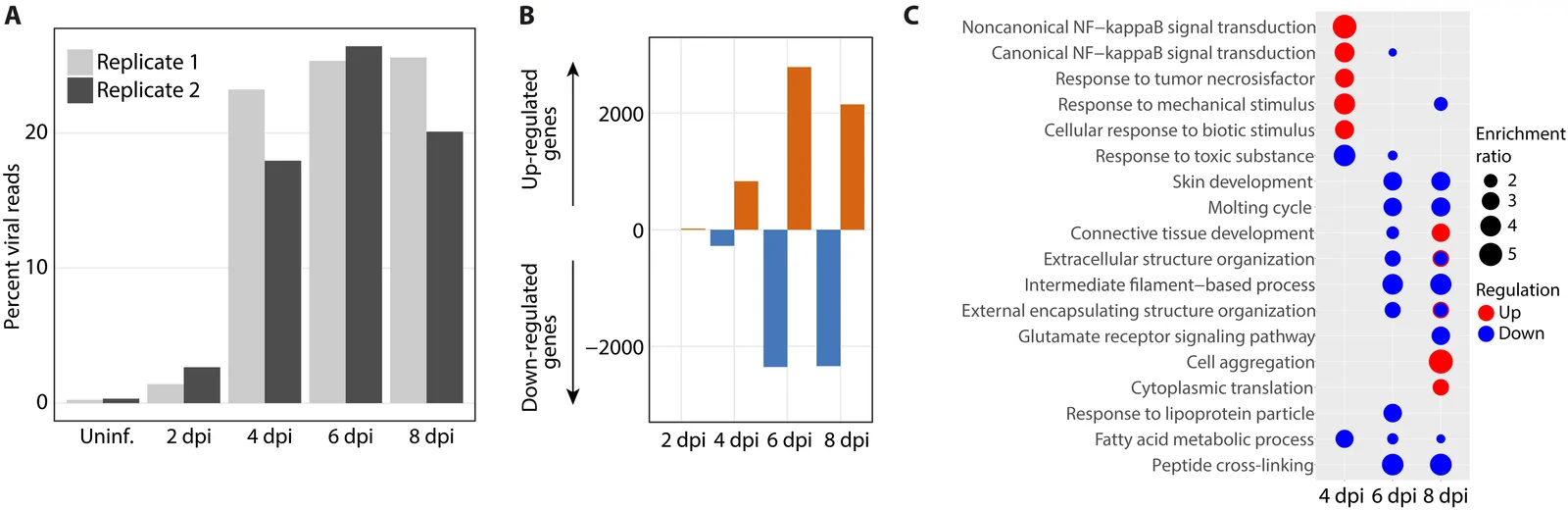
Bulk RNA-seq of HSV-1–infected SkOs. (**A**) Percentage of total reads mapping to HSV-1 in uninfected SkOs and at 2, 4, 6, and 8 dpi. Each bar shows one replicate. (**B**) Barplot showing numbers of differentially expressed host genes at 2, 4, 6, and 8 dpi versus uninfected SkOs. (**C**) GO analysis (WebGestalt) was performed using significantly (*P*_adj_ ≤0.05) up- or down-regulated genes (log2FC ≥1/≤−1) from RNA-seq analysis of SkOs infected with HSV-1 at 4, 6, and 8 dpi compared to uninfected SkOs. Shown are the top five significantly enriched GO terms (FDR ≤ 0.01, sorted by enrichment ratio) in the up- as well as down-regulated genes of each time point. At 2 dpi, no GO terms were significantly enriched. At 4 dpi only 2 GO terms were associated with the down-regulated genes. At 6 dpi, no GO terms were significantly enriched in the up-regulated genes. Overlapping blue and red color at 8 dpi indicates enrichment of the GO term in the up- as well as down-regulated genes.

### Spatial transcriptomics of HSV-1–infected SkOs

To understand the spatiotemporal dynamics of the viral infection and the host response in the SkO model system, we performed two spatial transcriptomics experiments with single cell resolution on the Xenium platform. In the pilot experiment, we profiled sections from three SkOs per condition, including uninfected SkOs and SkOs at 2 dpi and 6 dpi. Because of a strong CPE- and HSV-1–induced host cell shutoff, cell-type annotation was not possible for most cells at the 6-dpi time point (Supplementary Text and figs. S5 and S6). We therefore focused on earlier time points of infection and analyzed uninfected SkOs and SkOs at 2, 3, and 4 dpi in the main experiment (Fig. 3 and figs. S7 to S11). In addition to the predesigned human multitissue panel (377 genes), we included 100 custom genes, including five viral ones (US1, UL27, UL29, UL54, and LAT). Custom host genes were selected to improve cell-type assignment as well as to investigate immune-related signaling events based on published literature (*21*–*25*) as well as prior bulk RNA-seq experiments. Cell-type identities were assigned based on cellular transcriptomes (Fig. 3, figs. S7 to S11, and table S1; see Supplementary Text for cell-type assignment strategy). We identified the expected cell types based on single-cell RNA-seq data and immunostaining performed with SkOs by Lee *et al.* (*18*, *25*). In addition, we could distinguish between subtypes that coincide with specific localization such as different fibroblast types (e.g., papillary 1, 2, and mesenchymal) that localize proximal or distal to the epidermis. Keratinocyte subtypes at different stages of differentiation were distinguishable and clearly spatially restricted. We distinguished stratum basale and stratum corneum while the stratum spinosum and granulosum were combined as one cluster, since the markers in our panel were not sufficient for separation. Cell types of the HFs (dermal sheath, outer root sheath, inner root sheath, matrix, bulge region, and dermal papilla) were clearly identified and matched their expected localization. We furthermore verified the expression of the HSV-1 entry receptors HVEM/TNFRSF14 and NECTIN-1 and especially found the latter to be broadly expressed on all cell types except chondrocytes (fig. S12).

**Fig. 3.**
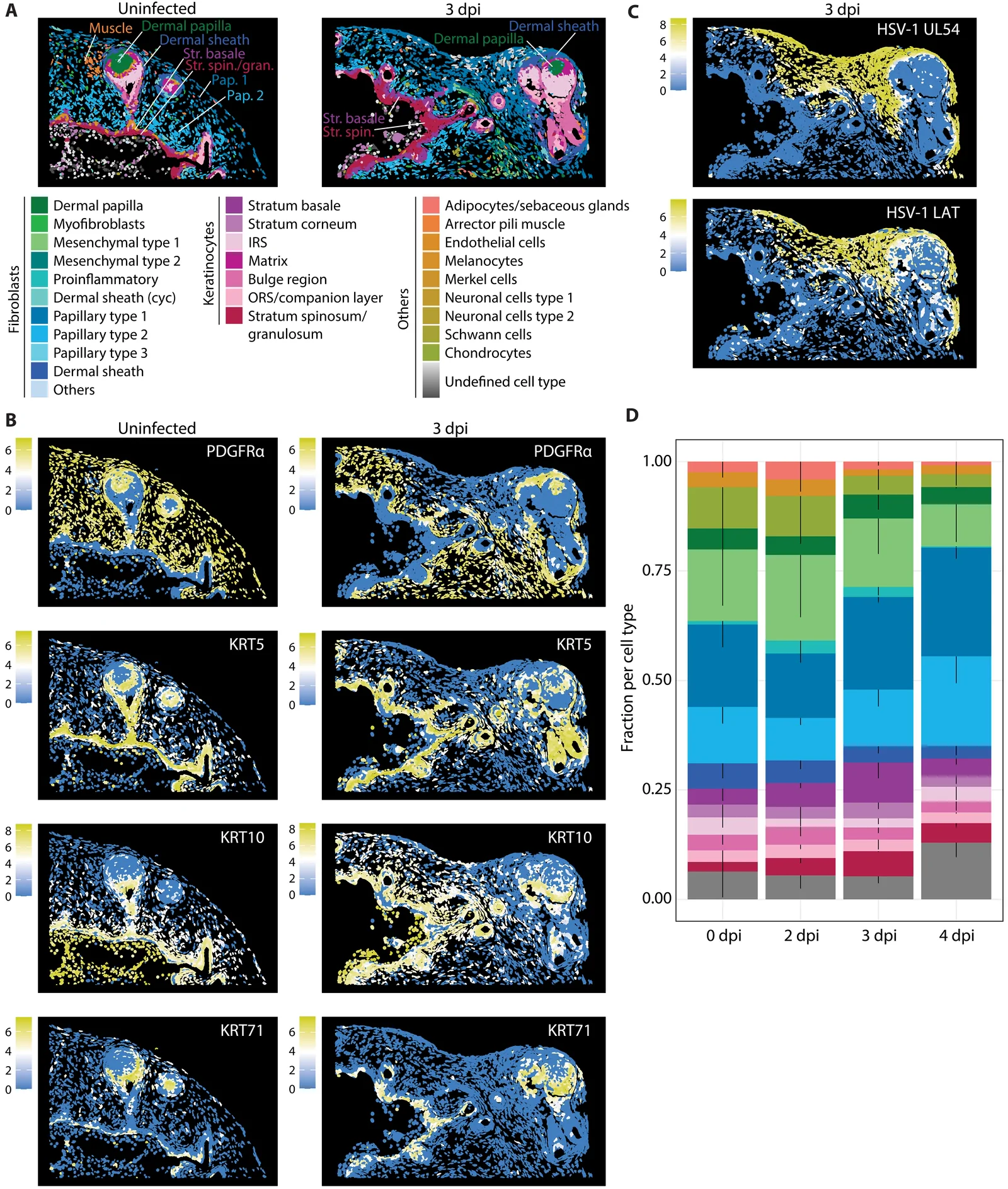
Spatial transcriptomics analysis of HSV-1–infected SkOs. (**A**) Representative regions from an uninfected SkO (left) and a SkO at 3 dpi (right), with cells colored by annotated cell type. (**B** and **C**) The same organoid regions as in (A), colored by expression levels of the indicated genes. (**D**) Relative abundances of major cell types across the indicated time points. Thin lines denote SDs calculated across three organoids per time point. Cell-type color coding is consistent with (A).

Cell-type assignments were also successful in infected SkOs (Fig. 3, A and D, and figs. S13 and S14), despite down-regulation of host transcripts in cells with high viral transcript levels. HSV-1–induced host cell shutoff was exemplified by the complete loss of PDGFRA transcripts, encoding the pan-fibroblast marker PDGFRα, in highly infected cells (*20*) (Fig. 3B, top). Therefore, some highly infected cells, particularly fibroblasts, lost their transcriptional identity and were thus assigned as “undefined” (Fig. 3, A and D, gray colored cells, and figs. S11A and S14). Accordingly, the proportion of “undefined” cells increased in highly infected (4 dpi) versus uninfected organoids but did not exceed ∼15% of all cells (Fig. 3D). Further analysis confirmed that this increase is due to the appearance of cells with high viral load (fig. S11C).

Spatial expression patterns of the viral transcripts US1/ICP22, UL54/ICP27, UL29/ICP8, UL27/gB, and LAT matched the protein staining, showing infection starting in the outermost fibroblast layers of the dermis at 2 dpi and then spread through the organoid until the infection reached the epidermis at 4 dpi (Fig. 3C and fig. S13). In summary, the spatial transcriptomics experiment identified the spectrum of expected cell types in SkOs and confirmed the gradual spread of the virus through the tissue.

### Susceptibility to viral infection varies widely between cell types

To investigate susceptibility and viral gene expression between different cell types in SkOs, we considered the spatial features of an infection spreading in a tissue compared to an infection in a monolayer cell culture. Protein staining and spatial transcriptomics showed that the majority of infected cells were in direct contact with other infected cells (Fig. 1 and figs. S2A and S13), suggesting that diffusion of viral particles within the tissue is either limited or slower than spread between neighboring cells. As a consequence, HSV-1 infects the outer layers of the organoids first and later the more inner parts leading to an asynchronous infection. To assess the susceptibility of the different cell types to viral infection, we first calculated the proportion of infected cells and the viral load in the most abundant subtypes, papillary fibroblasts type 1/2, keratinocytes of the stratum basale and stratum spinosum/granulosum, and four cell types in HFs (dermal sheath, dermal papilla, outer root sheath, and inner root sheath; Fig. 4A). At 3 dpi, papillary fibroblasts type 1, which is the outermost cell layer of SkOs, and the dermal sheath fibroblasts had the highest number of HSV-1–infected cells (∼25%). Also, papillary fibroblasts type 1 had a significantly higher viral load than all other cell types including the dermal sheath. At 4 dpi, when the infection reached the inner parts of the organoid, the proportion of infected cells increased in all cell types. However, stratum basale and spinosum/granulosum keratinocytes still showed significantly less infected cells than papillary fibroblasts (Fig. 4A, top). Viral loads were significantly lower than in papillary fibroblasts type 1 in all cell types except papillary fibroblasts type 2, which reside in the dermis directly below the epidermis (Fig. 4A, bottom). To exclude the idea that this effect was caused by the asynchronous infection spreading from the outside to the inside, we compared stratum basale keratinocytes with papillary fibroblasts at equal distance ranges from the exterior surface (fig. S15, A and B). Again, we found significantly lower viral loads. Furthermore, we analyzed the influence of infectious cells in proximity to basal keratinocytes (<50 μm distance) (fig. S16A). Infectious cells were defined as cells with 20% or more viral transcripts. As expected, we found an increase of the viral load with rising numbers of infectious cells in the neighborhood. Interestingly, papillary fibroblasts reached their maximum viral load (up to 70% viral transcripts) with one to two infectious cells in proximity. Keratinocytes of the stratum basale showed a significantly lower viral load compared to papillary fibroblasts despite the same number of infectious cells in their neighborhood. Even with >6 infectious cells in proximity, the mean viral load did not exceed 25% of all transcripts. To analyze the dynamics of the viral life cycle, we ordered and binned infected cells along increasing viral load. We found in papillary fibroblasts a strong increase of UL54 RNA at the beginning followed by a plateau, with the LAT RNA constantly accumulating (Fig. 4B, top). In stratum basale keratinocytes, there was an indication of a longer lag phase and a much slower increase of viral gene expression (Fig. 4B, bottom). These results suggest that keratinocytes of the stratum basale have a delayed or restricted viral life cycle.

**Fig. 4.**
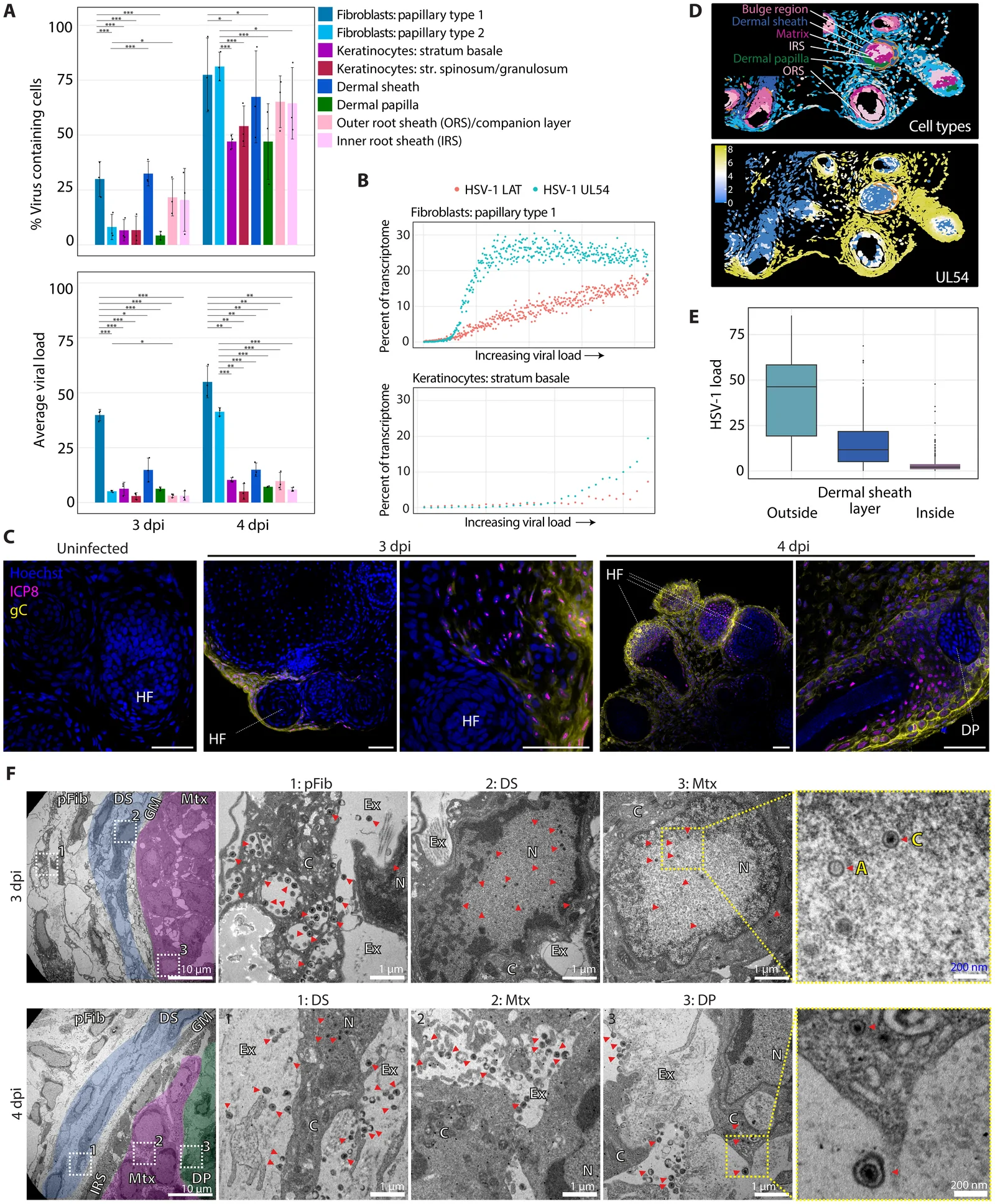
Susceptibility to viral infection varies widely between cell types. (**A**) Percent of cells with at least two HSV-1 transcript counts (top part) and average viral loads (bottom part) showing individual values from three SkOs per time point and SDs. Displayed are significant changes in comparison to papillary fibroblasts type 1/2 determined with a generalized linear mixed-effects model (GLMM) (*P_adj_ < 0.05, **P_adj_ < 0.01, ***P_adj_ < 0.001). All *P* values are listed in table S4. (**B**) Cells with at least two viral counts were ordered according to viral load and binned with 20 cells per bin. Shown are percentages of HSV-1 UL54/LAT transcripts within the bin for papillary fibroblasts type 1 (top) and stratum basale keratinocytes (bottom). (**C**) Sections from WMI staining of HSV-1–infected SkOs stained for HSV-1 proteins ICP8 and gC. Scale bars, 50 μm. (**D**) HSV-1 UL54 expression around HFs in a region from a SkO at 4 dpi. Top: Cells colored by cell type. Bottom: Cells colored by normalized HSV-1 UL54 expression. Surrounded with orange are dermal sheath cells in proximity to highly infected papillary fibroblasts. (**E**) Box plot of HSV-1 load (percent viral transcripts). Dermal sheath layer: Dermal sheath cells next to highly infected cells [surrounded with orange in (D)]. Outside/inside: Other cells within 50-μm distance from dermal sheath layer outside/inside HFs. (**F**) Transmission electron microscopy (TEM) of HFs from HSV-1–infected SkOs at 3 and 4 dpi. Left: Overviews with colored layers of HFs. pFib, papillary fibroblasts; DS, dermal sheath; GM, glassy membrane; Mtx, matrix; IRS, inner root sheath; DP, dermal papilla; Ex, extracellular space; N, nucleus; C, cytoplasm. Boxes with white and yellow lines indicate areas of zoom-ins. Red arrowheads indicate viral particles, and A and C denote capsids in a nucleus of a matrix cell.

In dermal sheath fibroblasts, the dermal papilla, and the keratinocyte subtypes of HFs (e.g., matrix and root sheaths), we also observed significantly lower viral loads than in the surrounding papillary fibroblasts (Fig. 4A). Staining for the viral early protein ICP8 and the late protein gC showed that viral infection spreads first into the dermis and excludes the HF layers (Fig. 4C). HF cell layers are infected after the infection already spread to the inner parts of the organoids at 4 dpi with the dermal papilla being infected last (Fig. 4C, right) but eventually infection reaches all layers of HFs with a CPE being observable at 6 to 8 dpi (Fig. 1B). We hypothesized three potential explanations for this observation. First, there could be a layer of nonpermissive cells surrounding the HFs. Second, a barrier of extracellular matrix might act protectively. Third, the infection cycle in the different cell types within the HFs could be slower compared to the surrounding papillary fibroblasts. To investigate these possibilities, we defined dermal sheath fibroblasts located next to highly infected papillary fibroblasts (fig. S16B). The viral load in these dermal sheath fibroblasts showed a strong drop from the cells outside in direct proximity (Fig. 4E). As for keratinocytes of the stratum basale, the lower viral load in cell types of HFs was not driven by spatial effects like the localization relative to the organoid boundary or a lower number of infected cells in proximity (figs. S15, A and B, and S16A). Lower viral transcript levels in HF-associated cells were not explained by reduced expression of the entry receptor genes NECTIN1 and TNFRSF14, which showed equal or higher transcript levels than in surrounding papillary fibroblasts (fig. S12). Immunofluorescence (IF) staining of the analyzed cells showed close contact between dermal sheath and papillary fibroblasts with no indication of any physical barriers between these cell layers (fig. S16C). This is supported by electron microscopy, which showed close proximity of papillary fibroblasts and dermal sheath fibroblasts (Fig. 4F and fig. S17). We observed that, at 3 dpi, many viral particles were present in the papillary fibroblast layer inside cells (nucleus/cytoplasm) as well as in the extracellular space (Fig. 4F, zoom-ins). In the dermal sheath layer, the matrix, the outer root sheath, and outer parts of the inner root sheath, only a small number of viral particles were detectable in the cytoplasm and the nucleus but not in the extracellular space. In the nuclei, we found viral A and C capsids indicating viral replication [A capsids lack DNA, B capsids lack DNA but contain the scaffolding protein, and C capsids contain the viral DNA (*26*)] (Fig. 4F, 3 dpi, zoom-in on matrix). At 3 dpi, no viral particles were observed in the innermost layers of the HFs (inner root sheath cuticle, companion layer, Huxley’s layer, and dermal papilla). However, at 4 dpi, we detected viral particles in the extracellular space, the cytoplasm, and the nucleus in all HF layers present in our sections (dermal sheath, outer root sheath, matrix, inner root sheath cuticle, companion layer, Huxley’s layer, and dermal papilla) except for Henle’s layer (Fig. 4F and fig. S17B). Again, we found A and C but also B capsids in replication compartments of the nucleus in these HF cell types. From these results, we concluded that HSV-1 lytically replicates in most cell types of HFs, but similar to keratinocytes of the stratum basale, viral replication is delayed in these cells, which slows down viral spread.

### Neurons in SkOs are infected by HSV-1 at very late time points

Apart from the distinct fibroblast and keratinocyte subtypes in dermis and epidermis, SkOs contain a complex neuronal system that innervates the HFs (*18*). Lee *et al.* (*18*) showed that these neurons express class III tubulin β (TUBB3) and compromise two major subtypes, neurons with medium-to-large somata expressing neurofilament heavy chain (NEFH) and peripherin (PRPH), and neurons with small somata that express PRPH but lack NEFH. The majority of neurons in SkOs are NEFH^+^ pseudounipolar neurons with S100β^+^ Schwann cells covering their axons and satellite glial cells covering their cell bodies (*18*). Accordingly, we found both neuronal subtypes in the SkOs generated by us by WMI staining (Fig. 5 and figs. S1, B to D, S18, and S19). In the spatial transcriptomics dataset, we identified two types of neuronal cells that express TUBB3 (Fig. 5, fig. S7, and table S1) with one expressing relatively higher levels of NEHF and the other expressing more PRPH (fig. S18B). However, in the tissue sections used for spatial transcriptomics, neuronal somata and axons could only be rarely and incomprehensively identified, thereby impairing analysis of viral gene expression on the single-cell level in these cells.

**Fig. 5.**
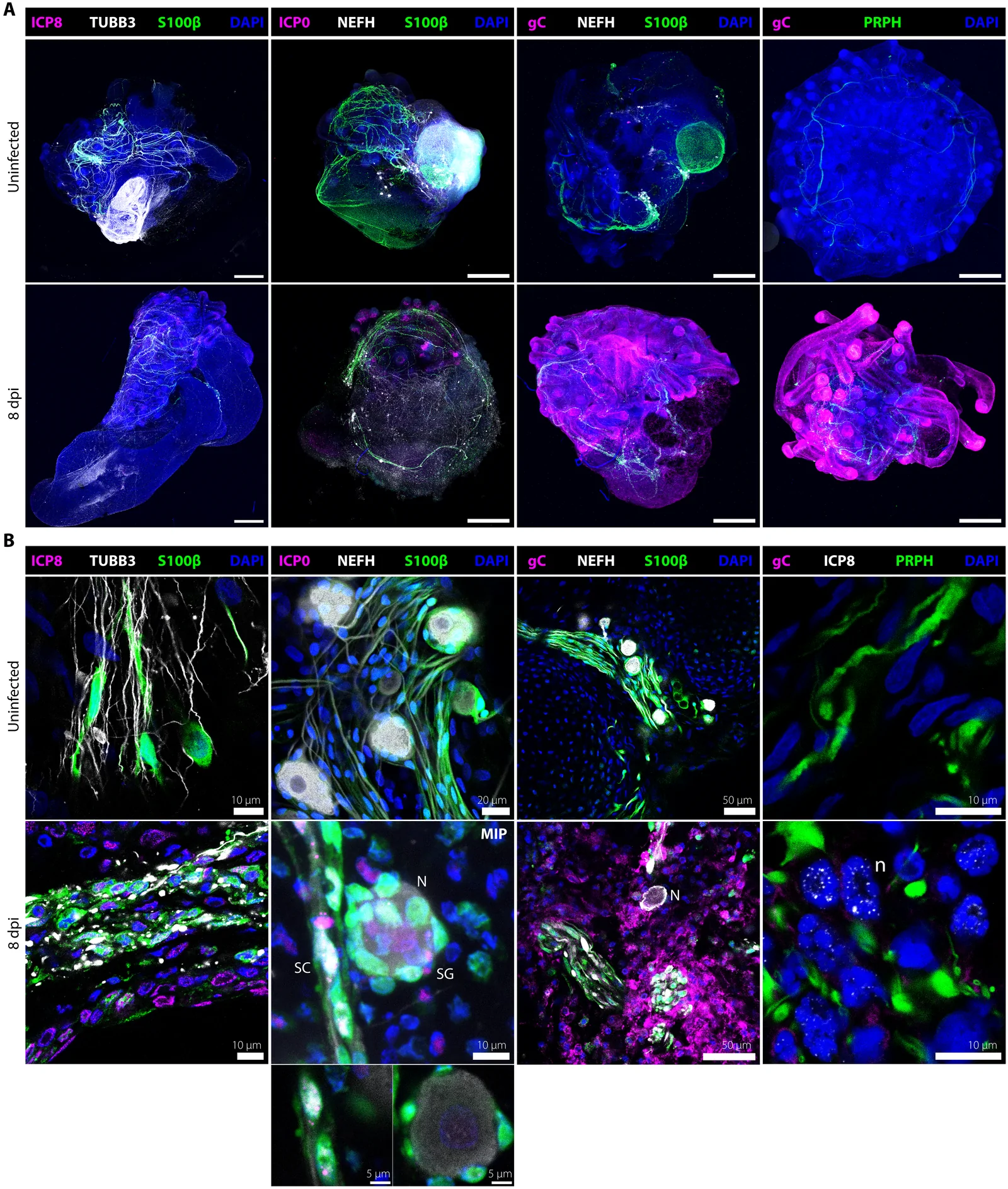
HSV-1 gene expression in neurons and Schwann cells of SkOs. (**A**) Overviews of WMI staining for TUBB3 (neurons), S100β (Schwann cells, satellite glial cells), NEFH (sensory neurons), PRPH (sensory neurons), and HSV-1 ICP8, ICP0, and gC in uninfected SkO and at 8 dpi. Shown are maximum intensity projections of acquired Z-stacks. Scale bars, 500 μm. Images displaying individual fluorescence channels are presented in fig. S18 (**B**) Zoom-ins on sections from WMI staining showing infection in neurons and Schwann cells. At 8 dpi, the first panel shows strong CPE of TUBB3^+^ neurons indicated by condensed TUBB3 staining and strong ICP8 expression in Schwann cells. The second panel shows a maximum intensity projection (MIP) from a Z-stack of a large NEHF^+^ soma (N) with satellite glial cells (SG) on the cell body and Schwann cells (SC) covering the axon. Pictures below are zoom-ins on sections from the Z-stack showing strong ICP0 expression in Schwann cells and diffuse weaker staining in nucleus of the NEFH^+^ neuronal soma. The third panel shows gC expression in a big NEFH^+^ neuronal soma (N). The fourth panel shows ICP8 and gC expression in a small PRPH^+^ neuronal soma (n). Images displaying individual fluorescence channels are presented in fig. S19.

We therefore focused on WMI staining on HSV-1–infected SkOs and assessed viral gene expression in TUBB3^+^ neurons, NEFH^+^ large soma neurons, and PRPH^+^ small soma neurons (Fig. 5 and figs. S18 and S19). At 8 dpi, we detected evidence of viral infection in the cell bodies of TUBB3^+^ neurons, NEFH^+^ large soma neurons, and PRPH^+^ small soma neurons through the expression of HSV-1 ICP0, ICP8, and gC (Fig. 5B), whereas no viral protein expression was detected at earlier time points. In PRPH^+^ soma, ICP8 foci in the nucleus together with gC expression indicated ongoing viral replication. The expression of gC and ICP0 was relatively low in NEHF^+^ cells in comparison to adjacent S100β^+^ Schwann cells and satellite glial cells (Fig. 5B). We could not assess ICP8 expression in NEFH^+^ neurons because the validated antibodies against ICP8 and NEFH were both mouse IgG1. Sequential staining could not be implemented within the WMI staining and clearing protocol used here, and we were unable to identify alternative NEFH or ICP8 antibodies with a compatible isotype that performed reliably under these staining conditions.

To investigate viral gene expression in the spatial transcriptomics dataset despite the inability to reliably identify neuronal cell bodies, we analyzed fully infected “neuronal regions” enriched for Schwann cells surrounding putative axons (fig. S19B). Although the overall viral load in these regions was only slightly lower than in neighboring fibroblast-only regions (fig. S19C), the ratio of LAT to UL54 transcripts was markedly increased (fig. S19D). While spatial transcriptomics cannot distinguish whether these viral transcripts originate from axons or Schwann cells, which is more likely due to their higher abundance and bigger size, the observed shift in the LAT-to-UL54 ratio suggests a distinct viral gene expression program within the neuronal compartment of SkOs.

Apart from the differential viral gene expression, the majority of neuronal cells exhibited CPEs comparable to those observed in other cell types at 8 dpi. The neuronal network displayed widespread cellular condensation and structural disruption (Fig. 5A and fig. S18). TUBB3^+^ axons and neuronal cell bodies showed pronounced degeneration, while infected PRPH^+^ neurons exhibited condensed chromatin (Fig. 5B and fig. S19). In contrast, a subset of NEFH^+^ cells that appeared in WMI staining were morphologically intact at 8 dpi despite viral gene expression (see ICP0 stain; Fig. 5B). Whether this reflects a transient delay in cytopathic changes or a more sustained resistance to HSV-1–induced damage remains unclear. Together, these findings indicate that the neuronal system in SkOs exhibits partial resistance to HSV-1 infection at early time points. However, this resistance is ultimately overcome, resulting in productive lytic infection and cytopathic degeneration in the majority of neuronal cells at later time points.

### IER3 is induced at the infection front

HSV-1 infection leads to a general shutoff of host genome transcription (*20*) as well as read-through (*27*) and antisense transcription (*28*), but also induction of several genes (*29*–*32*). We used the spatial transcriptomics data to investigate how host gene expression changes are related to the spread of the virus through the tissue. First, we focused on papillary fibroblasts, the most abundant infected cell type. We subclustered cells based on a set of 190 genes deregulated upon infection in papillary fibroblasts (Fig. 6A and fig. S20A). By projecting the clusters back onto the section of a 3-dpi organoid shown in Fig. 3, we observed that the transcriptome-based clustering is reflected in space (Fig. 6B and figs. S20B and S21). On the one hand, this allowed us to estimate the initial infection site from which the virus gradually spread through the tissue. On the other hand, we could use the gene expression data to investigate what happens at, before, and after the “infection front” in a spatiotemporal manner. We calculated DEGs between sets of papillary fibroblasts at different locations, which showed that the host gene IER3 is strongly induced at the infection front, with a subsequent decrease in cells later in the viral life cycle (Fig. 6C and fig. S20, A to C). This is consistent with the transient changes observed in bulk RNA-seq (fig. S20D). To corroborate our findings at the protein level, we performed IER3/ICP8 costaining in whole-mount organoids (Fig. 6D). ICP8 was used as a reliable marker of HSV-1 infection progression in this model. IER3 protein expression was detectable at the infection front and, in contrast to IER3 mRNA, remained detectable in cells behind the infection front that had progressed to later stages of the viral life cycle. IER3 is a target gene of the MAP kinase pathway (*33*) and HSV-1 infection has been shown to stabilize IER3 through ICP27 via this pathway (*34*–*36*). Consistently, UL54/ICP27 and IER3 transcript expression overlapped at the infection front. However, whereas IER3 transcript levels decreased behind the infection front, UL54/ICP27 expression remained high (Fig. 6C). As we were unable to establish reliable staining for ICP27 protein in SkOs, we cannot determine from our data whether the sustained IER3 protein expression observed in infected cells is mediated by ICP27.

**Fig. 6.**
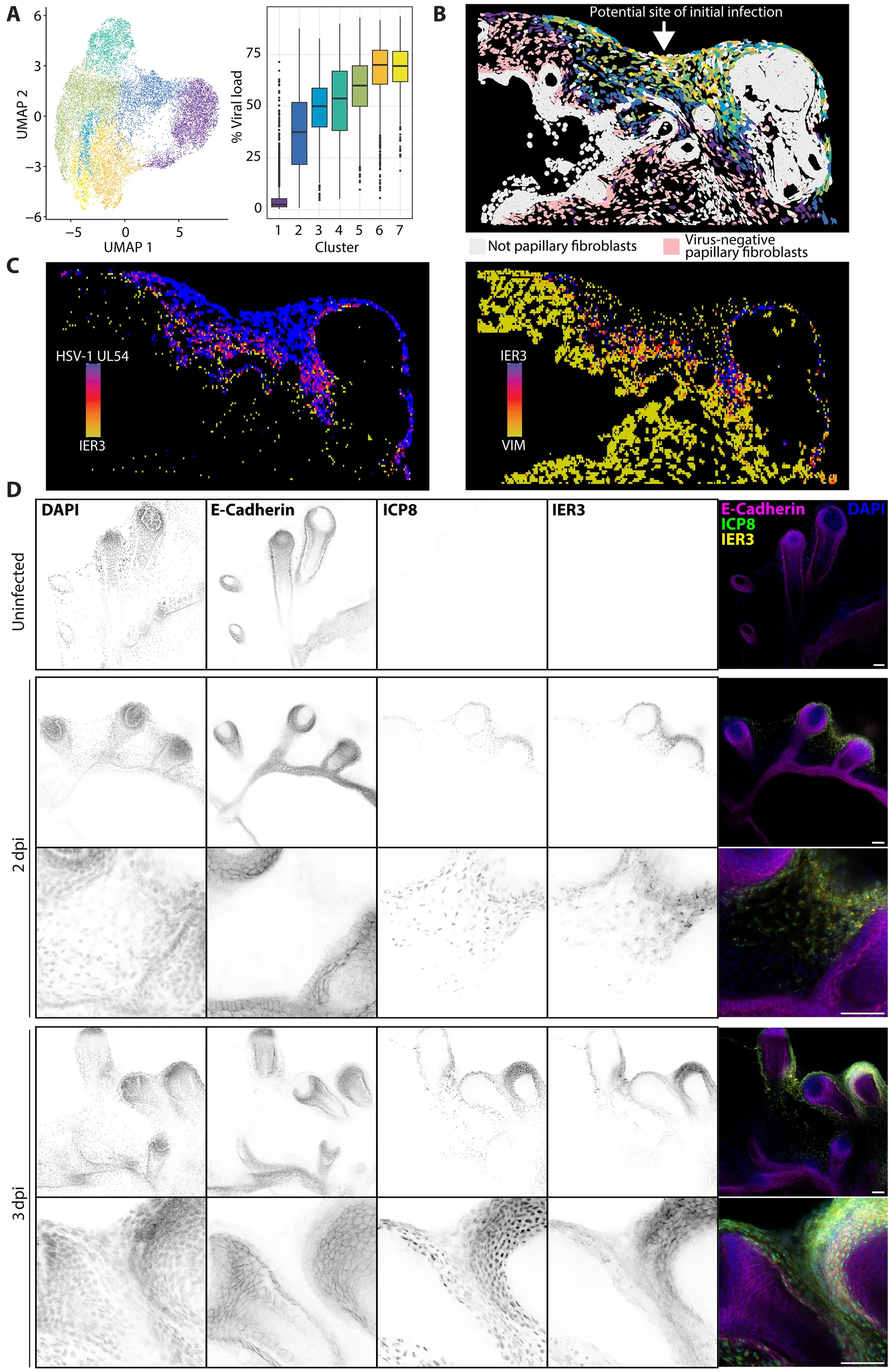
IER3 is induced at the HSV-1 infection front in SkOs. (**A**) Papillary fibroblast types 1 and 2 with at least two viral RNA counts were clustered based on viral gene expression and infection-induced host gene dysregulation, resulting in seven distinct clusters. Left: Two-dimensional projection of cells colored by cluster membership. Right: Boxplot showing the percentage of viral transcripts in the cells of each cluster. (**B**) Region of an infected organoid at 3 dpi shown in Fig. 3. Papillary fibroblasts are colored by cluster membership as in (A), or in light pink if containing less than two viral RNA counts (papillary fibroblasts without virus). Other cell types, including “undefined” cells, which includes highly infected papillary fibroblasts are in light gray. (**C**) Shown are transcript densities in papillary fibroblasts for genes as indicated in spatial bins. Red colors indicate that both transcripts are present. VIM, vimentin, general marker for fibroblasts. (**D**) Sections from WMI of uninfected and HSV-1–infected SkOs at 2 and 3 dpi stained for ICP8, IER3, and E-cadherin (marker for epidermis). Scale bars, 50 μm.

We also used the infection front analysis to investigate whether the virus continuously spreads into the organoid from the outer rim, or whether viral particles can physically penetrate the tissue and establish infection foci within the organoid. Except for one site, we found in all organoids a gradual progression through the previously defined infection stages from the organoid rim to the inside (fig. S21). Overall, this example shows how genes can be transiently up-regulated at the infection front as infection progresses through the tissue, thus creating a specific virus–host cell interaction microenvironment.

### HSV-1 infection up-regulates the transcription factor IRF3 in keratinocytes

Because defining an “infection front” requires a high number of infected cells from a specific cell type, we could not perform this approach with the lower abundant cell types in our model system. To still identify host genes responding to the infection, we calculated differential expression values per cell type between infected and uninfected organoids. We separated cells into (i) no viral RNA (less than two HSV-1 counts), (ii) low viral RNA (viral load below the median calculated within the cell type and time point but across all individual organoids), and (iii) high viral RNA (viral load above the median) (Fig. 7A). As positive controls, DUX4, antisense, and read-through transcripts were all found to be induced in cells with high viral RNA, although to different extent in different cell types (fig. S22A).

**Fig. 7.**
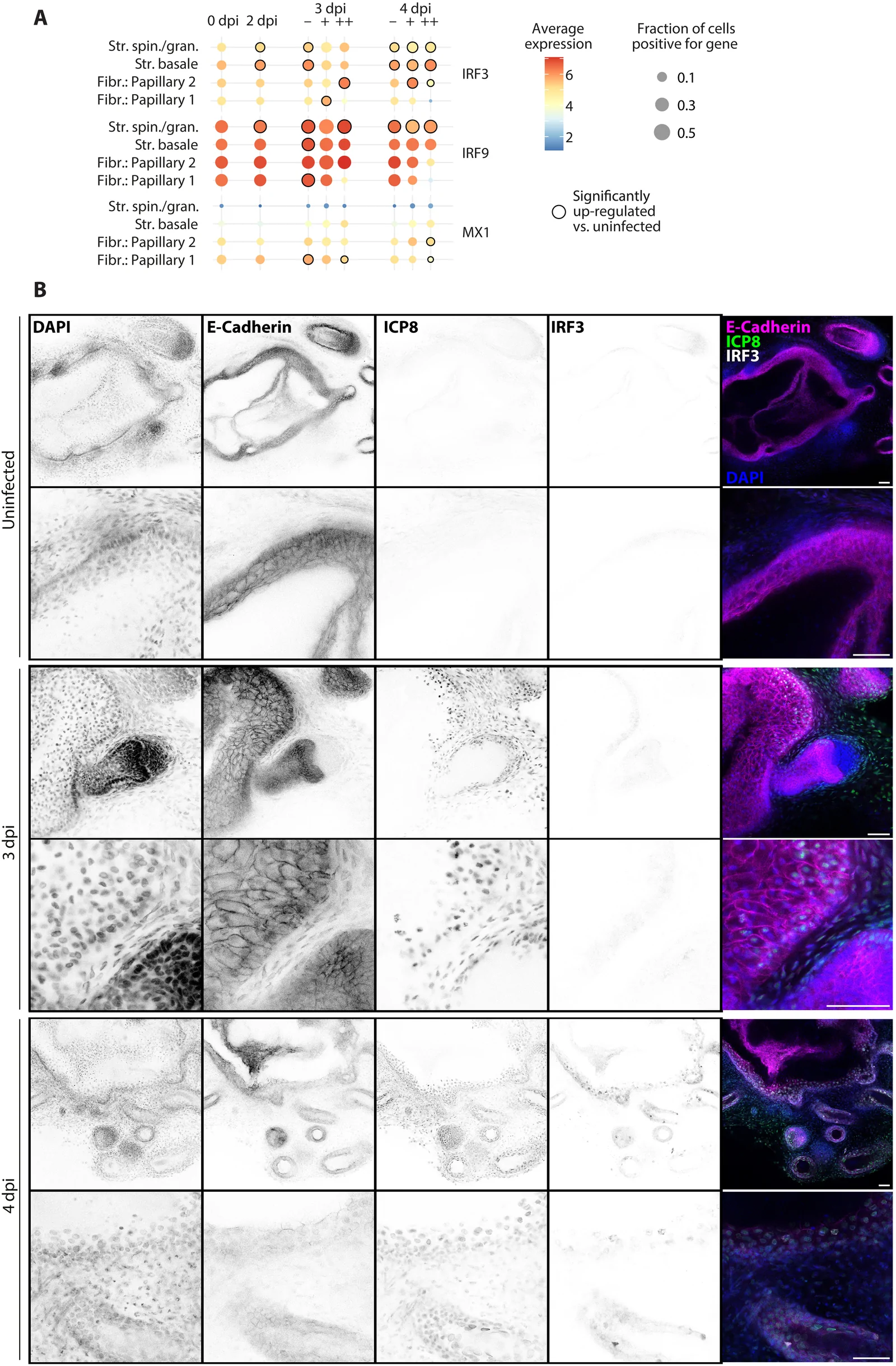
The transcription factor IRF3 is induced in keratinocytes. (**A**) Expression values of the indicated genes in a subset of cell types. For uninfected and 2 dpi SkOs, all cells were combined for the analysis. For 3 and 4 dpi, cells are split up in those with no or only one viral count (−), and those with viral load below (+) and above (++) the median. The median viral load was calculated only within cells with more than one count. Dot sizes represent the fraction of cells having at least one count of the specified gene, and the color denotes the average expression. Black circles indicate significant differential expression (*P*_adj_ < 0.05) in a pseudobulk comparison versus all cells in uninfected cells determined with DESeq2. All *P* values are listed in table S4. (**B**) Sections from WMI of uninfected and HSV-1–infected SkOs at 3 and 4 dpi stained for ICP8, IRF3, and E-cadherin (marker for epidermis). Scale bars, 50 μm.

Owing to the absence of immune cells in the SkO model, we were primarily interested in the innate immune response to HSV-1 infection. Because HSV-1 is well known to modulate the interferon (IFN) response, we focused on genes involved in IFN signaling and present in the gene panel, namely, the transcription factors IFN regulatory factor 3 and 9 (IRF3/IRF9), as well as the antiviral, IFN-stimulated gene (ISG) MX dynamin-like GTPase 1 (MX1) (*37*). For MX1, we did not find a strong up-regulation (Fig. 7A). This is in line with the observation that IFN-α, IFN-β, and IFN-γ included in our panel showed no up-regulation. Also, in bulk RNA-seq, we did not detect up-regulation of IFNs or IFN-stimulated genes at any time point. For IRF9 and particularly IRF3, however, we observed clear indications of up-regulation in keratinocytes early on, including cells not containing any viral RNA (Fig. 7A). This effect was not well detectable in bulk RNA-seq (fig. S22B). As a confirmation, we detected up-regulation of IRF3 also on the protein level, but only when keratinocytes were virus positive (Fig. 7B). This likely reflects the lag time during which newly synthesized protein accumulates until detectable levels are reached, but still confirms that IRF3 is up-regulated in response to the infection.

### HSV-1 induces a spatially coordinated expression of TNF family cytokines

In our bulk RNA-seq experiment, we found that genes involved in response to TNF were overrepresented in the significantly up-regulated genes at 4 dpi (Fig. 2C). The cytokines TNF (coding for TNF-α) and TNFSF9 (coding for 4-1BBL) were significantly up-regulated by HSV-1 infection in bulk RNA-seq (fig. S22B). Notably, spatial transcriptomics showed that their expression is highly cell type dependent and largely mutually exclusive, with TNF induced in keratinocytes (mainly stratum basale) and TNFSF9 in papillary fibroblasts with expression of both cytokines clearly depending on viral presence (Fig. 8A). Their expression increased with increasing viral load up to a plateau level (Fig. 8B). Published data from HSV-1–infected fibroblasts (*27*) and HaCat keratinocytes (*38*) support the findings of a cell type–specific up-regulation of TNFSF9 in fibroblasts and TNF in keratinocytes. Both the bulk RNA-seq data generated here in SkOs and the published bulk RNA-seq did not indicate that up-regulation of TNF and TNFSF9 is the result of HSV-1–induced read-in transcription from an upstream gene. To investigate whether expression of TNF would lead to functional sensing in surrounding cells, we defined papillary fibroblasts proximal to TNF-expressing cells (fig. S22C) and analyzed them for induction of TNF-α target genes (Fig. 8D) based on a published RNA-seq dataset generated in fibroblasts (*39*). By calculating the differential expression in cells proximal versus distal to the TNF-expressing cells, we found that four of the eight genes included in our panel were significantly up-regulated especially in cells without viral gene expression (SERPINB2, CXCL10, CXCL2, and TNF; Fig. 8D). We found a high number of cells expressing CXCL2, a TNF-α–induced gene, in the epidermis and in the dermis close to TNF-expressing keratinocytes (Fig. 8C, top row, last panel). To investigate whether this could also be caused by 4-1BBL (TNFSF9), we analyzed a region with high TNFSF9 but low TNF expression (Fig. 8C, bottom row). CXCL2 levels in these regions were lower, suggesting that the cell type–specific induction of the TNF gene leads to secretion of the TNF-α cytokine and subsequently induction of target genes in the immediate surrounding cells. To confirm the induction of the aforementioned genes not only on the RNA but also on the protein levels, we conducted IF-IHC on 4-1BBL and CXCL2 in HSV-1–infected SkOs at 4 dpi. Similar to the spatial transcriptomics, virus-positive cells were also positive for 4-1BBL, whereas CXCL2 was also found in virus-negative cells in close proximity to infected locations (Fig. 8E and fig. S23). Together, the data indicate that SkOs respond to HSV-1 infections with cell type–dependent, functional expression of TNF cytokine family members.

**Fig. 8.**
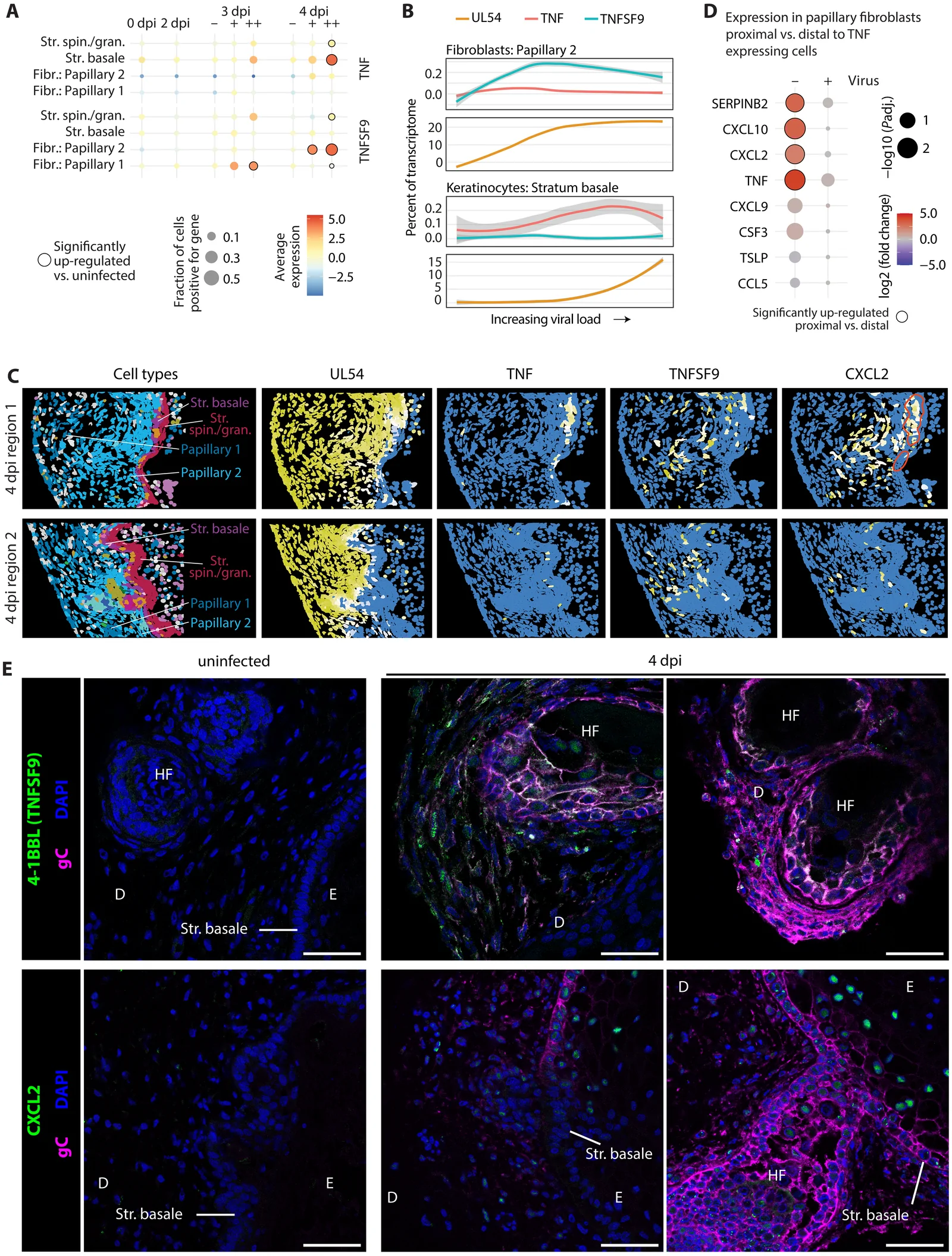
TNF induction leads to proximal expression of target genes. (**A**) Expression values of the indicated genes in a subset of cell types. For uninfected and 2 dpi SkOs, all cells were combined for the analysis. For 3 and 4 dpi, cells are split up in those with no or only one viral count (−), and those with viral load below (+) and above (++) the median. The median viral load was calculated only within cells with more than one count. Dot sizes represent the fraction of cells having at least one count of the specified gene, and the color denotes the average expression. Black circles around the dots indicate significant differential expression (*P*_adj_ < 0.05) in a pseudobulk comparison versus all cells in uninfected cells determined with DESeq2. All *P* values are listed in table S4. (**B**) Cells with at least two viral counts were ordered along viral load and binned with 20 cells per bin. Shown are the percentages of TNF, TNFSF9, and HSV-1 UL54 transcripts within the bin for papillary fibroblasts type 2 (top) and stratum basale keratinocytes (bottom). (**C**) Shown are two regions of a 4-dpi organoid, with cells colored by cell type or expression levels of the indicated genes. Cells encircled in red in the CXCL2 expression panel at 3 dpi are expressing TNF. (**D**) Differential expression of TNF target genes in papillary fibroblasts proximal versus distal to TNF-expressing cell groups determined with DESeq2. Black circles indicate significant differential expression (*P*_adj_ <0.05). All *P* values are listed in table S4. (**E**) IHC staining of SkOs uninfected and at 4 dpi for expression of HSV-1 gC and 4-1BBL (TNFSF9) and CXCL2. D, dermis; E, epidermis; HF, hair follicle; Str. basale, stratum basale. Scale bars, 50 μm. Images displaying individual fluorescence channels are presented in fig. S23.

### Differential gene expression programs and viral restriction in response to IFN-β and TNF-α

The spatial transcriptomics experiment showed induction of TNF family cytokines dependent on HSV-1 infection. In addition to 4-1BBL (Fig. 8E), we confirmed the presence of TNF-α upon infection in SkO supernatant by enzyme-linked immunosorbent assay (ELISA; Fig. 9A). Next, we analyzed the influence of TNF-α and 4-1BBL treatment on transcriptome, chemokine production, and virus replication (Fig. 9, B and C, and fig. S24). We could confirm that both CXCL2 and CXCL10 are induced by infection and, much stronger, by TNF-α treatment, with 4-1BBL having no or only a minute effect (Fig. 9B). This was recapitulated in a bulk RNA-seq experiment (Fig. 9C and fig. S24, C and D). As a comparator, we treated SkOs with IFN-β, which induced two classical ISGs (ISG15 and MX1) regardless of infection (Fig. 9C). Whereas TNF-α alone did not induce IFN-β, its combination with infection led to IFN-β induction, coinciding with ISG expression (Fig. 9C). Viral replication was strongly inhibited by IFN-β as expected, but not by TNF cytokines (fig. S24, A to C). We therefore conclude that their primary role is not to induce an antiviral state in neighboring cells, but rather to activate immune cells that are absent from the SkOs.

**Fig. 9.**
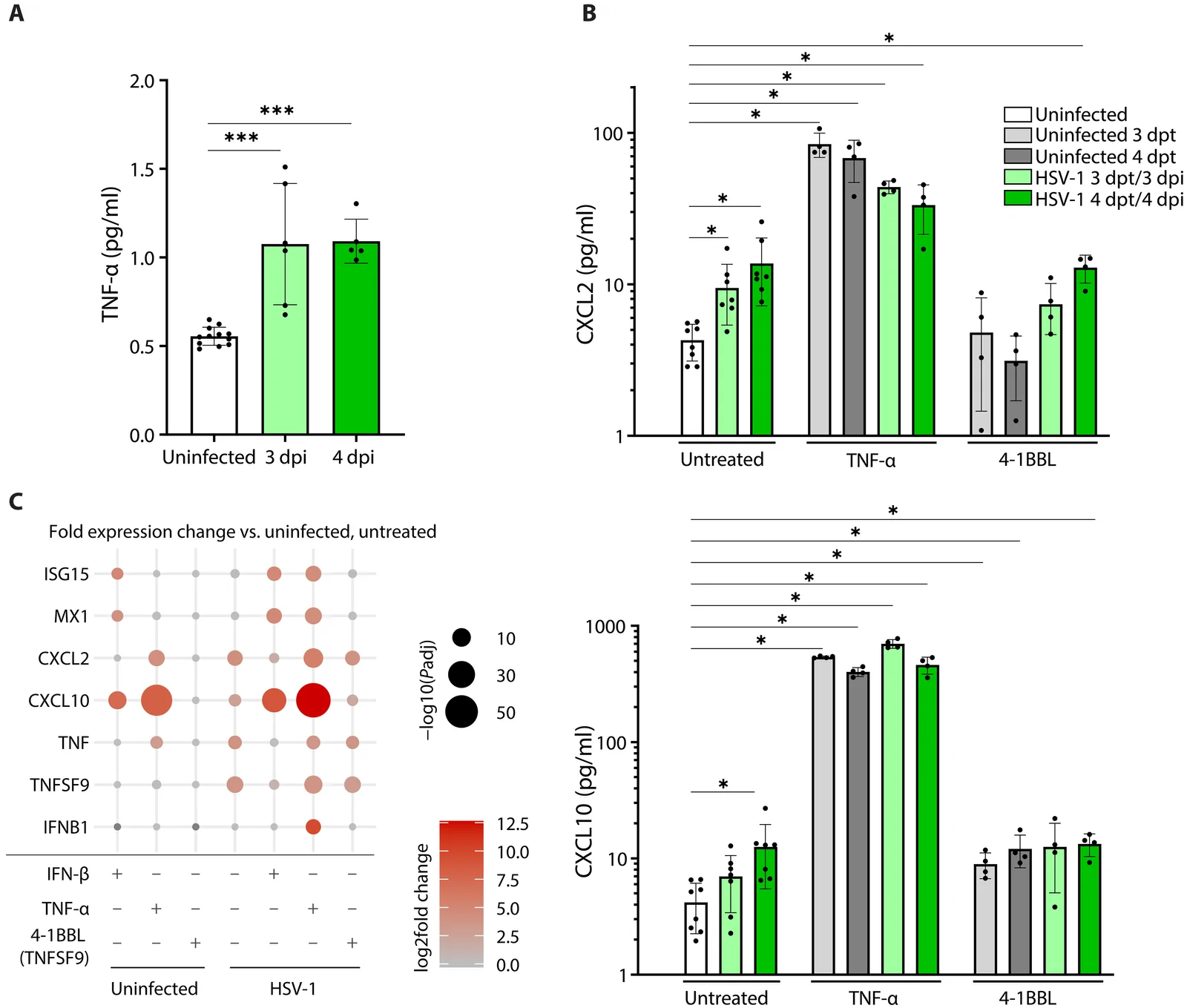
HSV-1–infected SkOs release TNF-α, CXCL2, and CXCL10. (**A**) ELISA measuring TNF-α release into the culture medium of HSV-1–infected SkOs. Biological replicates were *n* = 12 for uninfected controls, *n* = 6 for 3 dpi, and *n* = 5 for 4 dpi HSV-1–infected SkOs. (**B**) ELISAs measuring CXCL2 and CXCL10 release into the culture medium of HSV-1–infected SkOs, either untreated or treated with the indicated factors. Biological replicates were *n* = 8 for uninfected untreated controls and *n* = 7 for both 3 and 4 dpi HSV-1–infected untreated groups; treated groups were *n* = 4 for all conditions. TNF-α or 4-1BBL (TNFSF9) treatment was performed simultaneously with infection. Statistical comparisons between groups shown in (A) and (B) were performed with pairwise Mann-Whitney *U* test followed by Benjamini-Hochberg adjustment of *P* values. Displayed are significant changes in comparison to the untreated group (*P_adj_ < 0.05, ***P_adj_ < 0.001). All *P* values are listed in table S4. Dpt, days posttreatment; dpi, days postinfection. (**C**) Bulk RNA-seq analysis of HSV-1–infected SkOs at 4 dpi, either untreated or treated with TNF-α, 4-1BBL (TNFSF9), or IFN-β, showing fold change in gene expression relative to uninfected, untreated SkOs. Adjusted *P* values were determined with DESeq2. All *P* values are listed in table S4. Heatmaps showing individual biological replicates are presented in fig. S24.

## DISCUSSION

In this study, we showed an infection of complex human hair–bearing SkOs with HSV-1. Infection with a low dose of HSV-1 resulted in a gradual spread of the virus from the outer to the inner regions of the organoid. When analyzing the susceptibility of the cells to HSV-1 in the SkO model, we found that eventually at 8 dpi, all cell types in our model system were infected by HSV-1 except for the terminally differentiated keratinocytes, cornified structures in the HFs, and chondrocytes in the tail. This is expected due to a lack of immune cells that could clear the infection. Among all cell types in SkOs, chondrocytes have the lowest expression level of the viral entry receptor NECTIN-1, which could explain their resistance to viral infection (fig. S12B). A resistance of the stratum corneum against HSV-1 infection has been reported by many other studies using mouse models and ex vivo skin showing that the cornified keratinocyte layer prevents HSV-1 infection by the external surface (*6*, *9*). The terminally differentiated keratinocytes of the epidermis in the SkO model express markers of the stratum corneum (LOR and KRT10); however, they do not eventually cornify because they are not exposed to air. Despite this difference, their complete resistance to infection is well in line with findings from other models. Studies also showed that infection of all other layers of the epidermis by HSV-1 strongly depends on the infection route and the model system (dermal versus epidermal, human versus mouse, and skin versus mucosal skin) (*40*, *41*). In human, ex vivo skin wounding from the external side through different approaches leads to only limited infection of the epidermis (*11*, *12*). Only infection from the dermal side by either removal of the dermis or deeper wounding through the epidermis into the dermis leads to the efficient infection of the basal keratinocytes and spread to the higher differentiated keratinocyte layers (*9*, *42*). In the SkO model, the dermis was efficiently infected by HSV-1 and viral loads in papillary fibroblasts were much higher than in keratinocytes of the stratum basale and stratum spinosum/granulosum despite the presence of infectious cells in the neighborhood. While the basal membrane as an extracellular matrix barrier likely slows down viral infection, we show evidence that the viral life cycle in keratinocytes is delayed compared to papillary fibroblasts. When we analyzed the spread of HSV-1 into HFs, we observed that dermal sheath fibroblasts pose a barrier to HSV-1 infection of the more inner layers. The close physical proximity of dermal sheath fibroblasts to heavily infected papillary fibroblasts, in the absence of an intervening or expanded extracellular matrix, suggests that extracellular matrix structures are unlikely to account for the observed barrier function. Furthermore, the finding that dermal sheath cells are not infected less frequently but instead harbor a reduced viral load indicates that this phenotype is unlikely due to limited accessibility of a viral entry receptor. This conclusion is supported by the abundant expression of NECTIN-1 in these cells and instead suggests that the reduced viral burden reflects a delayed progression of the viral life cycle in this cell type. Our electron microscopy impressively shows that eventually almost all cell types in the HFs produce mature viral particles that were found to be intra- and extracellular. We hypothesize that the delayed HSV-1 infection in HFs could be important during reactivation when viral particles are released by the nerve endings of sensory neurons that innervate HFs. A restriction of the viral life cycle in dermal sheath fibroblasts could give immune cells time to counteract the infection. In the future, we would like to test this hypothesis by further analyzing the immune reaction in dermal fibroblasts and by including immune cells into the SkO model.

Analyzing HSV-1 infection in the neuronal system of SkOs proved challenging with spatial transcriptomics due to the low abundance of neuronal cell bodies. Analysis by three-dimensional WMI staining revealed a partial resistance of neuronal cells to HSV-1 infection, resulting in delayed infection compared with other cell types. Nevertheless, by later time points, most neuronal cells displayed evidence of lytic viral replication. Although we observed indications of distinct viral gene expression in neurons, there was no evidence for latency establishment. The establishment of HSV-1 latency in the peripheral nervous system relies on many different factors, among them the site of viral entry at the axon (*2*). In the SkO model, peripheral neurons innervate HFs, while their cell bodies are organized either in ganglion-like clusters or as individual neurons within the organoid tail. Consequently, HSV-1 can access neurons from multiple locations rather than exclusively through distal axons. This mode of infection may limit the establishment of latency and instead favors productive lytic infection.

The inside-out morphology of SkOs and the infection therefore spreading from the dermis to other cell types represent a limitation of the model system for studying primary HSV-1 skin infection. However, this infection direction also recapitulates aspects of HSV-1 reactivation, when infectious particles are released from neuronal cells into the fibroblast layer of the dermis (*43*). This makes our model highly relevant for investigating HSV-1–induced pathologies, because most of them are a result of reactivation and not primary infection. For instance, recurrent herpes labialis has an average incidence of about 1.6 per 1000 patients each year (*44*). Conversely, the fact that viral particles are not initially released from reactivating neurons in SkOs, but that the neuronal compartment is infected at later stages, more closely resembles aspects of primary HSV-1 infection involving deep dermal wounding. In the future, combining different organoid systems representing the skin and neuronal compartments through coculture approaches could enable the development of a more physiologically relevant HSV-1 skin reactivation model.

Despite these limitations, the analysis of the host response demonstrated a highly physiologically relevant, coordinated, and cell type–specific immune and inflammatory response to HSV-1 infection, highlighting the ability of the model to recapitulate key aspects of host-virus interactions. We analyzed these responses at the single-cell level and further investigated their spatiotemporal dynamics by examining cells located at the infection front within the dermis. We found IER3 transcripts specifically up-regulated in papillary fibroblasts at the leading edge of the infection. IER3 mRNA contains an AU-rich instability element (ARE) in its 3′ untranslated region, a feature that promotes rapid mRNA degradation by deadenylation under normal conditions and stabilization by proinflammatory signaling like the p38 MAPK pathway (*45*, *46*). Many studies have shown before in cell culture that the IER3 mRNA is stabilized by HSV-1 (*34*, *35*), particularly by ICP27 (*36*). Transcript up-regulation was transient, while the up-regulated protein was very stable in the heavily infected cells in SkOs. A study using HeLa cells showed a transient induction of IER3 protein expression 1 hour postinfection (hpi) and a rapid decline already at 3 hpi (*35*). This could indicate a different turnover of the IER3 protein in tissue in comparison to a cell line. The biological significance of IER3 induction during HSV-1 infection remains unclear. IER3 has been reported to exert context-dependent pro- and antiapoptotic functions (*47*) and, while it can itself be induced by TNF-α, has also been shown to protect cells from TNF-α–induced cell death (*48*, *49*). In SkOs, pronounced infection-associated cell death was observed only at a relatively late stage of infection, at 6 dpi (fig. S4). We therefore hypothesize that the persistence of IER3 protein in cells after the infection front may contribute to maintaining cell viability despite ongoing viral replication and increasing inflammatory signaling, including TNF-α. Such prolonged survival of infected cells could provide additional time for viral replication and cell-to-cell spread and may thereby facilitate dissemination into more resistant cell populations, including neurons. However, functional experiments targeting IER3 will be required to determine whether it indeed contributes to cell survival or viral spread in this model.

The fact that we found a well-known target of HSV-1 infection through our spatiotemporal analysis shows the validity of the approach. It allows a comprehensive analysis of spatial transcriptomics data from infected tissues that goes beyond the discrimination between cell types and viral loads. However, IER3 was one of only three genes specifically up-regulated at the infection front. This is likely an effect of the limited number of host genes included in our spatial transcriptomics experiment (470 genes) and we speculate that panels with more genes included will be even more informative to understand how a tissue reacts to a locally spreading infection. Another limitation of this approach is that to define an infection front, a certain number of cells from the same cell type is needed; thus, we could perform this only for papillary fibroblasts of the dermis. Thus, for lower abundant and spatially more distributed cells, we compared uninfected cells and cells with low viral load and high viral load assuming that cells with a higher viral load have been infected for a longer time. We found that IRF3 is up-regulated at the transcript and protein level in keratinocytes of the stratum basale. Similar to IER3, we find a delayed effect of protein induction in comparison to the transcripts and no induction of IRF3 on protein level in virus-negative cells. Because we do not find induction of IFN-α, IFN-β, or IFN-γ, or the ISGs included in our panel, we can only speculate which downstream signaling might be induced by IRF3. An indication for a potential antiviral effect is that IRF3 protein is mainly up-regulated in infected keratinocytes of the stratum basale where we also found lower viral loads in comparison to fibroblasts of the dermis.

The host response to HSV-1 infection in SkO is further dominated by inflammation. Spatial transcriptomics showed that TNF (coding for TNF-α) was induced in keratinocytes of the stratum basale and TNFSF9 (coding for 4-1BBL) in papillary fibroblasts. We also observed induction of TNF target genes in surrounding cells and a potential priming of adjacent uninfected cells, indicating that gene activation is indeed functional. Induction of TNF and subsequent tissue damage were previously associated with HSV-1 in cerebral infections (*24*). In ex vivo mouse whole corneal culture, HSV-1 spread was reduced by TNF treatment but also resulted in increased cell death (*50*). Treatment of SkOs with TNF-α resulted in a strong induction of CXCL2 and CXCL10 expression. In contrast to IFN-β treatment, however, TNF-α stimulation did not significantly reduce viral replication. This finding is consistent with the primary function of these chemokines, which is to recruit and activate immune cells capable of controlling viral infection, rather than directly inducing antiviral activity in the tissue cells. Similarly, treatment of SkOs with 4-1BBL did not reduce viral replication, likely because the cognate receptor, 4-1BB (CD137), is predominantly expressed on activated immune cells rather than on the resident cell populations present in SkOs. We hypothesize that in an immune-competent system, TNF signaling may contribute to viral control through the recruitment and activation of immune effector cells with the drawback of increased tissue damage. Conversely, this offers opportunities to specifically reduce HSV-1–induced inflammation and tissue damage by treatment with inhibitors for TNF-α or 4-1BBL.

To test this hypothesis, incorporation of immune cells and vascular components into the SkO model system will be essential, representing a key limitation of the current model. Recent studies included autologous T cells in human intestinal organoids (*51*) or reported the successful incorporation of macrophages and improved angiogenesis in SkOs (*25*). Building on these advances, future work will aim to further refine this model for HSV-1 and deepen our understanding of HSV-1 pathogenesis in the skin.

In summary, our findings establish SkOs as a physiologically relevant model for studying HSV-1 infection in human skin. Combining this model with single-cell–resolution spatial transcriptomics enabled a detailed analysis of viral gene expression, tissue spread, and cell type–specific host responses. Compared with ex vivo skin models, SkOs offer several advantages, including the potential for genetic manipulation and prolonged maintenance in culture. These features may make SkOs particularly suitable for investigating HSV-1 mutants with impaired replication or growth. Future advances in organoid technology, including multiorganoid coculture systems and the integration of immune and vascular components, may further enhance the physiological relevance of this model and expand its applications to the study of viral persistence, latency, host-virus interactions, and antiviral therapies.

## MATERIALS AND METHODS

### SkO generation

SkOs were generated from the human iPS cell line UKEi001-A (RRID:CVCL_A8PR). All procedures involving hiPSC lines were approved by the local ethics committee in Hamburg (Az PV4798, 28.10.2014). HiPSCs were cultivated in six-well plates coated with Geltrex (Thermo Fisher Scientific, catalog no. A1413302) in StemFlex medium (Gibco, catalog no. A3349401) under standard conditions (37°C, 5% CO_2_). Generation of SkOs was performed according to the protocol by Lee *et al.* (*19*) with hiPSCs between passages 31 and 39. Only SkOs showing correct differentiation as determined by the presence of HFs at 110 to 120 days postdifferentiation were used for further experiments.

### Virus infection

HSV-1 17 CMV-IEproEGFP expressing EGFP under the control of the CMV IE promoter was described before (*52*). The virus was propagated on Vero cells, and the infection titer was determined by plaque assay. Medium on SkOs was exchanged and infection was performed by adding 800 PFU per SkO to 1 ml of standard SkO growth medium. After 12 hours, the medium was exchanged with standard SkO growth medium, and afterward, complete medium exchange was performed every day. For comparison of HSV-1 infections in SkOs with NHDF cells, the number of cells on the organoid surface was estimated to be approximately 8 × 10^4^ cells. Accordingly, 8 × 10^4^ NHDF cells were seeded in 1 ml of DMEM + 10% fetal bovine serum + 1% penicillin/streptomycin in a 12-well plate. NHDF cells and individual SkOs cultured in 1 ml of medium in a 12-well plate were infected with 8 × 10^2^, 8 × 10^3^, or 8 × 10^4^ PFU HSV-1 (viral titer determined on Vero cells), corresponding to MOIs of 0.01, 0.1, and 1, respectively.

### Cytokine treatment of SkOs

Treatment of SkOs with cytokines was performed simultaneously to viral infection. Afterward, cytokines were added freshly at the 12-hour postinfection media change and every 24-hour media change. Uninfected controls were treated accordingly. The following concentrations of cytokines were used: 1000 U/ml TNF-α (Peprotech, no. 300-01A-50UG), 100 ng/ml 4-1BBL (Peprotech, no. 310-11-20UG), and 100 U/ml IFN-β (Peprotech, no. 300-02BC-20UG).

### Enzyme-linked immunosorbent assays

For detecting cytokines and chemokines in the medium of SkOs, the following ELISAs were used: LEGEND MAX High Sensitivity Human TNF-α ELISA Kit (BioLegend, no. 430217), Human GRO-β (CXCL2) Standard ABTS ELISA Development Kit (Peprotech, no. 900-K120K), and Human IP-10 (CXCL10) Standard ABTS ELISA Development Kit (Peprotech, no. 900-K39K). ELISAs were performed with 50 μl of culture supernatant from SkOs diluted with 50 μl of phosphate-buffered saline (PBS) according to the manufacturer’s instructions. Absorbance measurements were performed with a Tecan Spark. Calculation of standard curves and concentrations, as well as visualization, was performed with GraphPad Prism 9.

### Quantification of HSV-1 DNA

HSV-1 DNA in the culture medium of infected SkOs or NHDF cells was quantified using a laboratory developed test for the simultaneous detection of HSV-1, HSV-2, and VZV DNA including an internal full-process control on a fully automated PCR system (cobas X800 series, Roche). For HSV-1, a dual-target approach was selected with primers and probes (HSV-1) adapted from Köller *et al.* (*53*) and Weidmann *et al.* (*54*). Validation of the assay was performed according to current European guidelines [Regulation (EU) 2017/746 on IVDR]. Oligonucleotides for HSV-1 were for target one (FAM): forward primer: GGC CTG GCT ATC CGG A(Ometh-G)A, reverse primer: G(+C)G (+C)(+A)G AGA CAT C, probe: CAG CAC ACG ACT TGG CGT TCT GTG T and for target two (FAM): forward primer: CGG CCR TGT GAC AYY AT(Ometh-C) G, reverse primer: CTC GTA AAA TGG CCC CT(Ometh-C) C, probe: CCV TAC CGA CMA CAY CGA CGA. Visualization of results was performed with GraphPad Prism 9.

### Immunostaining and microscopy

For IHC, SkOs were fixed in 4% phosphate-buffered formalin overnight at 4°C and then transferred to PBS. Afterward, they were protected in 3% agarose, dehydrated using a Leica ASP300S tissue processor, and embedded in paraffin. Organoid paraffin sections were cut at 3 μm and stained with hematoxylin and eosin (H&E) according to standard procedures. For IHC staining, the sections were processed as follows: After dewaxing and inactivation of endogenous peroxidases (3% hydrogen peroxide), antibody-specific antigen retrieval was performed using the Ventana BenchMark XT autostainer (Ventana, Tucson, AZ, USA). Sections were blocked and afterward incubated with the primary antibodies (table S3). For detection of specific binding and DAB staining, the UltraView Universal DAB Detection Kit (Roche, no. 760-500) was used, which contains both anti-mouse and anti-rabbit secondary antibody. Counter staining and bluing were performed with hematoxylin (Ventana Roche, no. 760-2021) and bluing reagent (Ventana Roche, no. 760-2037) for 4 min. Subsequently, stained sections were mounted in mounting medium. For IF-IHC staining, organoid paraffin sections were cut at 5 μm and thoroughly deparaffinized in 2× 15 min in xylene and a descending alcohol row. Antigen retrieval was performed by steam heating the sections in Universal R buffer (no. AP0530-500; Aptum, UK) for 45 min at 95°C (Steamy 800; Sommertal). Sections were briefly rinsed and blocked for 1 hour (MAXblock, Active Motif). Antibodies (see table S3) were incubated overnight at 4°C. After intensive washing, Alexa Fluor 488–, Alexa Fluor 568–, or Alexa Fluor 647–coupled secondary antibodies (see table S3) were applied for 1 hour. Sections were washed again, counterstained with DAPI, and mounted in Fluoromount-G (SouthernBiotech, Birmingham, USA).

Pictures of IHC staining were acquired with the Sysmex Pannoramic MIDI II slide scanner or with a Leica TCS SP8 X confocal microscope. Image cropping and processing was performed with QuPath0.4.3, Imaris, and ImageJ 2.14, and pictures were assembled with Adobe Illustrator.

For whole mount staining of SkOs, the qCe3D method was used according to Lee *et al.* (*19*) with the following adjustments: Clearing was performed for 48 hours, and stained and cleared SkOs were mounted in iSpacer (SUNJinLab) four-well imaging spacers. For antibodies, see table S3. Imaging was performed with a Leica TCS SP8 X confocal microscope. Images were processed with Imaris and ImageJ 2.14 and pictures were assembled with Adobe Illustrator.

Life bright-field and fluorescence images of SkOs were acquired with an EVOS M5000 microscope (Thermo Fisher Scientific). Images were cropped and processed with ImageJ 2.14 and pictures were assembled with Adobe Illustrator.

### Electron microscopy

SkOs were fixed overnight in 2% paraformaldehyde and 2.5% glutaraldehyde, washed in PBS, and postfixed in 1% osmium tetroxide for 60 min. Following additional rinses in PBS and double-distilled water, samples were stained with 1% aqueous uranyl acetate for 60 min, dehydrated through a graded ethanol series, and embedded in Epon (Carl Roth). Ultrathin sections (50 nm) were prepared using an Ultracut microtome (Leica Microsystems) and poststained with 1% uranyl acetate in ethanol. Transmission electron micrographs were acquired on a FEI Tecnai G20 Twin operated at 80 kV and equipped with a 2K CCD Veleta camera (Olympus SIS). To correlate TEM data across entire organoids, serial sections were mounted on silicon wafers and imaged using a Tescan Clara SEM with backscattered-electron detection at 2.8 kV.

### TUNEL and cleaved caspase-3 staining

Analysis of cell death in SkOs was performed with the Click-iT Plus TUNEL Assay Kit for In Situ Apoptosis Detection (Thermo Fisher Scientific, no. C10618). The assay was performed on 5-μm FFPE sections of paraffin-embedded SkOs according to manufacturer’s instructions including the optional DNase digest for a positive control. The optional antibody staining was performed for cleaved caspase-3 (Cell Signaling, no. 9661T, dilution 1:200) and HSV-1 gC (Santa Cruz Biotechnology, no. sc-51626, dilution 1:50) overnight at 4°C. Secondary antibody was used, and nuclear stain and mounting was performed as described for IF-IHC. Images were acquired with a Leica TCS SP8 X confocal microscope. Image cropping and processing was performed with ImageJ 2.14 and pictures were assembled with Adobe Illustrator.

### Bulk RNA-seq

For bulk RNA-seq, four SkOs per condition (uninfected and 2, 4, 6, and 8 dpi) were used. Total RNA was isolated individually from each SkO by adding QIAzol lysis reagent (Qiagen) and disrupting the organoid by grinding with a pestle. The lysate was homogenized with a QIAshredder (Qiagen). After one round of chloroform extraction, RNA was cleaned up with the RNeasy MinElute Cleanup Kit (Qiagen). DNA was two times digested with the DNA-free DNA removal kit (Thermo Fisher Scientific). RNA concentration was determined with the Qubit RNA broad range assay (Thermo Fisher Scientific), and equal amounts of RNA from two SkOs of the same condition were pooled, resulting in duplicates for each condition for sequencing. RNA integrity was determined via a Bioanalyzer RNA 6000 Pico Assay (Agilent, 5067-1514). RIN values were in the range between 5.5 and 9.2. From each pooled sample, 520 ng of total RNA was subjected to rRNA depletion (following the Lexogen RiboCop rRNA Depletion Kit for Human/Mouse/Rat V2 protocol, catalog number 144.24) and further processed via the RNA-Seq V2 Library Prep Kit with UDIs (Lexogen, catalog number: 175.96) in the short insert size variant (RTM) according to the manufacturer’s instructions. Libraries were quality controlled on a Bioanalyzer High Sensitivity DNA Assay (Agilent, 5067-4626) and were sequenced on an Illumina NextSeq 500 instrument with NextSeq 500/550 High Output Kit v2.5 (75 Cycles, product number: 20024906) in a single-read mode (1× 83 bp). After demultiplexing via bcl2fastq, 20.7 to 28.6 million (M) read pairs were assigned to each sample. All samples passed quality control by fastqc and were subjected to downstream analysis. Processing was done with the nf-core/RNA-seq pipeline (v3.12.0) within the nf-core framework (*55*). The pipeline was executed with Nextflow v23.04.1 (*56*), using STAR (v2.6.1d) (*57*) for alignment to the GRCh38 human genome and Human herpesvirus 1 genome (X14112.1), as well as Salmon (v1.10.1) (*58*) for transcript-level quantification based on STAR’s alignment output. To eliminate duplicate reads, the --with_umi option was enabled. Gene-level quantification was derived from transcript-level estimates using the R package tximport (v1.34.0) (*59*), which sums transcript abundances for each gene. The generated raw gene-level counts were normalized and variance stabilized using the regularized log transformation (rlog) from DESeq2 (v1.34.0) (*60*), addressing the variance-over-mean trend. Principal components analysis (PCA) confirmed high reproducibility across biological replicates. Differential expression analysis was performed using DESeq2, identifying genes as significantly differentially expressed (DEGs) if they exhibited a fold change ≥2 and an adjusted *P* value ≤0.05. The normalized rlog-transformed counts were then used for clustering and visualization. An overrepresentation analysis of differentially expressed host genes (*P*_adj_ ≤ 0.05 and log2FC ≥1/≤−1) was performed with WebGestalt (*61*). GO terms associated with “Biological processes, nonredundant” were selected with an FDR ≤0.01.

For bulk RNA-seq of cytokine-treated SkOs, three SkO were used for each condition, except for INF-β–treated SkOs (one replicate uninfected and two replicates infected). RNA was isolated and DNase digested as described before, without pooling RNA from individual SkOs. RIN values were in the range between 6 and 9. From each sample, 600 ng of total RNA was converted to an RNA-seq library using the NEBNext Ultra II Directional RNA Library Prep Kit with polyA RNA selection for Illumina (NEB, catalog no. E7760 and E7490) according to the manufacturer’s instructions (60% reaction volume). Libraries were quality controlled on a Tape Station D1000 Assay (Agilent, catalog no. 5067-5582) and were sequenced on an Illumina Novaseq plus instrument in 2× 151-bp mode to obtain 16 to 25 read pairs per sample. Reads were aligned to a combined human/HSV-1 genome using hisat2 (*62*) and counted using featureCounts (*63*). PCA confirmed high reproducibility across biological replicates. Differential expression analysis was performed using DESeq2, and tpm values were used for gene expression analysis of individual samples.

### Spatial transcriptomics experiments

SkOs were imbedded in paraffin as described for IHC staining. Three SkOs for each condition were assembled in one agarose mold before embedding all conditions (uninfected/2 dpi/3 dpi/4 dpi for the main experiment and uninfected/2 dpi/6 dpi for the pilot experiment). In situ RNA measurements were performed using the Xenium system (10x Genomics). The pilot experiment was done at the MDC/BIH genomics platform using nuclear expansion based on DAPI staining for cell segmentation. The main experiment was performed at the 10x Genomics facility in Stockholm using multimodal segmentation (nuclear stain, RNA and protein-based cell staining, and cell surface staining). For both experiments, the human multitissue gene panel was used, supplemented by the 100-gene custom panel CZBXK3 (table S2). Sections of 5 μm thickness were placed on a Xenium slide according to the manufacturer’s protocol, with drying at 42°C for 3 hours and overnight placement in a desiccator at room temperature, followed by deparaffinization and permeabilization to make the mRNA accessible. The Probe Hybridization Mix was prepared according to the user guide (CG000582, Rev D, 10x Genomics). The staining for Xenium was performed using Xenium Nuclei Staining Buffer (10x Genomics product number 2000762) as a part of the Xenium Slides & Sample Prep Reagents Kit (PN-1000460). Following the Xenium run, H&E staining was performed on the same section according to the Post-Xenium Analyzer H&E Staining user guide (CG000613, Rev B, 10x Genomics).

### Spatial transcriptomics analysis

The spatial transcriptomics data were analyzed in R using the VoltRon package (*64*), as well as packages from tidyverse (*65*). Other packages used include DESeq2 (*60*), apeglm (*66*), sf (*67*), and concaveman (*68*). Briefly, we first assessed cell-type identity only on cellular transcriptomes, i.e., without taking spatial information into account (table S1 and see Supplementary Text for more information). Viral load was defined as percent viral counts (i.e., sum of the five viral genes UL54, US1, UL27, UL29, and LAT) of all counts per cells. Cells with 10 or less cellular RNA counts were filtered out. Organoid boundaries and distances from these boundaries were identified using the concaveman/st_boundary/st_distance function from the concaveman (*68*) and sf (*67*) R packages. To classify cells based on the number of infectious cells in the neighborhood, the absolute number of cells with more than 20% viral load and within 50 μm (centroid to centroid distance) was counted for every cell. Differential expression using DESeq2 (*60*) was calculated as pseudo-bulk per cell type, i.e., counts were summed up per group (uninfected and 2 dpi all cells, 3 and 4 dpi either cell with one or less viral count “−,” viral load below “+,” or above the median “++” within the group). The infection front analysis was initiated by clustering papillary fibroblasts with at least two viral counts using only genes that are differentially expressed upon infection. Cells of cluster 1, with the lowest viral load, which were in groups of at least five cells within 150 μm, were defined as the infection front. Cells with less than two viral counts and within 50 μm of these cells were classified as “uninfected next to infection front.” For the TNF proximity analysis, cells were defined as “TNF expressing,” if at least three cells with at least one TNF gene count were not more than 30 μm apart from each other, and the proximal area defined at 280 μm around these cells. ChatGPT model 5 (OpenAI, https://chat.openai.com) and Devstral 2 123b instruct 2512 (Mistral, running on academiccloud.de) assisted in writing R code. To compare “neuronal regions” with fibroblast-containing control regions, areas of interest were manually annotated in Xenium Explorer, and region statistics (area and transcript counts) were exported and processed in R for quantitative analysis.

### Biological replicates and statistical analyses

For the quantification of viral DNA in the supernatants of HSV-1–infected NHDF cultures and SkOs by qPCR (fig. S2B), three biological replicates were analyzed for each MOI and model (*n* = 3 independent NHDF cultures and *n* = 3 SkOs). For the quantification of viral DNA in the supernatants of HSV-1–infected SkOs following treatment (fig. S24B), *n* = 4 SkOs were analyzed for the untreated group and the TNF-α–and 4-1BBL–treated group, and *n* = 3 SkOs for the IFN-β–treated group. Supernatants from the same SkOs were collected and analyzed longitudinally at the indicated time points. Statistical analysis was performed with aligned rank transform (ARTool package) (*69*) with the formula log(copies/ml) ∼ treatment × day + (1|treatment:well) where treatment and days are represented as fixed effects and well is represented as a random effect. The *art.con* function was used to adjust *P* values of pairwise differences using the Holm method.

For bulk RNA-seq of HSV-1–infected SkOs at 2, 4, 6, and 8 dpi (Fig. 2 and figs. S3, S20D, and S22B), four SkOs were collected per time point. Equal amounts of RNA from two SkOs of the same time point were pooled, resulting in two independently pooled RNA samples per time point for sequencing. For bulk RNA-seq of untreated and cytokine-treated SkOs (Fig. 9C and fig. S24, C and D), the following numbers of biological replicates were analyzed: uninfected/untreated, *n* = 3; uninfected/IFN-β–treated, *n* = 1; uninfected/TNF-α–treated, *n* = 3; uninfected/4-1BBL-treated, *n* = 3; HSV-1–infected/untreated, *n* = 3; HSV-1–infected/IFN-β–treated, *n* = 2; HSV-1–infected/TNF-α–treated, *n* = 3; and HSV-1–infected/4-1BBL–treated, *n* = 3.

For Xenium spatial transcriptomics, *n* = 3 SkOs were analyzed per experiment and time point. The main experiment comprised uninfected SkOs and HSV-1–infected SkOs at 2, 3, and 4 dpi (*n* = 3 SkOs per time point), while the pilot experiment comprised uninfected SkOs and HSV-1–infected SkOs at 2 and 6 dpi (*n* = 3 SkOs per time point).

To compare gene expression values between conditions in bulk and spatial transcriptomics (Figs. 7A, 8, A and D, and 9C and figs. S20, C and D, and S22, A and C), we used the DESeq2 package in R, using the lfcShrink function (with type = “apeglm”) (*66*) to shrink overestimated fold changes. This function uses a Wald test with Benjamini-Hochberg multiple-testing correction to adjust *P* values.

For the statistical testing of presence or absence of virus or viral RNA content in Fig. 4A and fig. S16A, we used generalized linear mixed-effects models (GLMM) using the lmer and lmerTest R packages (*70**,* *71*). For the top part of Fig. 4A, the variable HSV1Status was defined as plus or minus depending on the presence of viral RNA. Subsequently, we tested the difference between each pair of cell types by a GLMM model, defining cell types as fixed effects and organoid replicates as random effects, i.e., with the formula: HSV1Status ∼ cell type + (1|cell type:organoid), with logit transform set as the link function [family = binomial(link = “logit”)]. Adjusted *P* values across cell-type pairs were then calculated using the FDR method, separately for each time point. For the bottom part of Fig. 4A, we used only cells containing viral RNA, and the normalized viral loads instead, i.e., the formula: HSV1Load ∼ cell type + (1|cell type:organoid), with identity link function [family = gaussian(link = “identity”)]. Figure 4A shows only adjusted *P* values for comparisons including papillary type 1 and 2 fibroblasts smaller than 0.05. For fig. S16A, the same method was used as for the bottom part of Fig. 4A, with adjusted *P* values calculated separately for each category.

For ELISAs shown in Fig. 9, A and B, each organoid was considered a biological replicate, and cell culture supernatants were collected at the indicated time points for analysis. For TNF-α ELISA, supernatants from 12 uninfected control SkOs and 6 HSV-1–infected SkOs were analyzed at 3 dpi. At 4 dpi, supernatant was available from only five HSV-1–infected SkOs; therefore, *n* = 5 biological replicates were analyzed. For CXCL2 and CXCL10 ELISAs, supernatants from eight untreated, uninfected SkOs and seven untreated, HSV-1–infected SkOs were analyzed at both 3 and 4 dpi.

For the TNF-α and 4-1BBL treatment experiments, *n* = 4 uninfected and *n* = 4 HSV-1–infected SkOs were used for each treatment condition at 3 and 4 days posttreatment (dpt). Cell culture supernatants were collected at 3 and 4 dpt and analyzed by ELISA. For all ELISA measurements, supernatants were analyzed as technical replicates. Standards were measured in triplicate according to the manufacturer’s instructions. ELISA standard curves were fitted using a sigmoidal four-parameter logistic nonlinear regression model with least-squares fitting, without weighting or parameter constraints, in GraphPad Prism 9.5.1. Individual replicate measurements were included as separate data points, and sample concentrations were interpolated from the fitted standard curves. The mean concentration of the two technical replicates was calculated for each biological replicate. Comparisons between groups were performed with pairwise Mann-Whitney *U* test followed by Benjamini-Hochberg adjustment of *P* values using the built-in R Stats package with the *wilcox.test* function. All *P* values and adjusted *P* values generated by the described statistical analyses above are available in table S4.

## Supplementary Materials

### The PDF file includes:

Supplementary Text Figs. S1 to S24 Tables S1 to S3 Legend for table S4 References
