# Supplementary material for: Human hair–bearing skin organoids reveal cell type–specific susceptibility and innate immune responses to herpes simplex virus 1
Source: Sci Adv. 2026 Oct 30;12(44):eaef5563. doi: 10.1126/sciadv.aef5563 (PMC13721972; doi:10.1126/sciadv.aef5563)
Supplement: Supplementary file 1 [file sciadv.aef5563_sm.pdf]

Science Advances
AAAS

Supplementary Materials for

Human hair–bearing skin organoids reveal cell type–specific susceptibility and innate immune responses to herpes simplex virus 1

Emanuel Wyler *et al.*

Corresponding author: Manja Czech-Sioli, m.czech-sioli@uke.de

Sci. Adv. **12**, eaef5563 (2026)
DOI: 10.1126/sciadv.aef5563

The PDF file includes:

Supplementary Text
Figs. S1 to S24
Tables S1 to S3
Legend for table S4
References

Other Supplementary Material for this manuscript includes the following:

Table S4

Supplementary Text

Processing and cell type annotation of spatial transcriptomics data

For the main experiment, three organoids per timepoint (uninfected as well as 2/3/4dpi) were measured on a Xenium slide. We used the pre-designed human multi-tissue and cancer panel with 377 genes as well as a custom 100 gene panel including 5 viral genes (table S2) for multiplexed transcript detection. For improved cell segmentation, we used the cell segmentation add-on kit, which allows segmentation based on DAPI staining, internal protein and rRNA detection, as well as cell surface staining. Raw data including cell segmentation was pre-processed on the Xenium device using manufacturer's defaults to yield the initial data folder, available through NCBI GEO (accession number GSE313919).

Subsequently, data was read into R for further analysis. Very low quality cells (less than 10 counts) were filtered out. To increase sensitivity for cell type detection, we included three uninfected organoids from an infection experiment run in parallel using skin organoids and Merkel cell polyoma virus infection *(72)*. Next, cells from all time points (i.e., three organoids each from this experiment uninfected/2/3/4dpi as well as three uninfected organoids from the polyoma virus experiment, in total 15 skin organoid sections) together were clustered based on their transcriptome using Seurat *(73)* (fig. S8). Expression of marker genes across clusters was used to manually assign clusters to cell types (fig. S9, table S1). Next to major fibroblast and keratinocyte clusters, we found a range of less abundant cell types (fig. S10). Some clusters were assigned as "undefined" cells, which frequently contained high amounts of viral RNAs (figs. S11C, S14). Likely, as a consequence cell type identity could not be assigned due to loss of marker genes. Of note, we also attempted cell type annotation following integration using harmony *(74)* or SCtransform *(75)*, as well as annotation using the BuildNicheAssay function in the Seurat package *(73)*, or BANKSY *(76)*. While these algorithms successfully identified the expected major cell types, they failed to detect low-abundance populations such as melanocytes and Merkel cells, which occur as single cells embedded within clusters of another cell type, for example in the stratum basale. In contrast, cell-type annotation based solely on cellular transcriptomes was superior, enabling robust detection of both major cell types and these rare populations.

Pilot experiment

Prior to the main experiment described above, which is the one presented in the main figures, we performed a pilot experiment. We used the same gene panels as described above, however with the timepoints uninfected/2/6dpi, and with DAPI-staining based nuclear expansion for cell segmentation instead of the cell segmentation add-on kit. Cells were re-segmented with a 10 µm expansion distance instead of the standard 15 µm distance using CellRanger (10x Genomics). Otherwise, cell types were assigned as described above for the main experiment (Fig. S5, S6). In contrast to the main experiment, we failed to identify several low-abundant cell types, which could be due to decreased sensitivity with the nuclear expansion method. At 6dpi, most cells could not be assigned to a cell type, likely due to high amounts of viral RNA across the entire organoid sections and the virus induced host cell shutoff. From the pilot experiment, we concluded that advanced cell segmentation using the cell segmentation add-on kit which we used in the main experiment was superior to nuclear expansion–based cell type annotation. In addition, by 6dpi, HSV-1 infection had progressed too far to be suitable for spatial transcriptomics experiments.

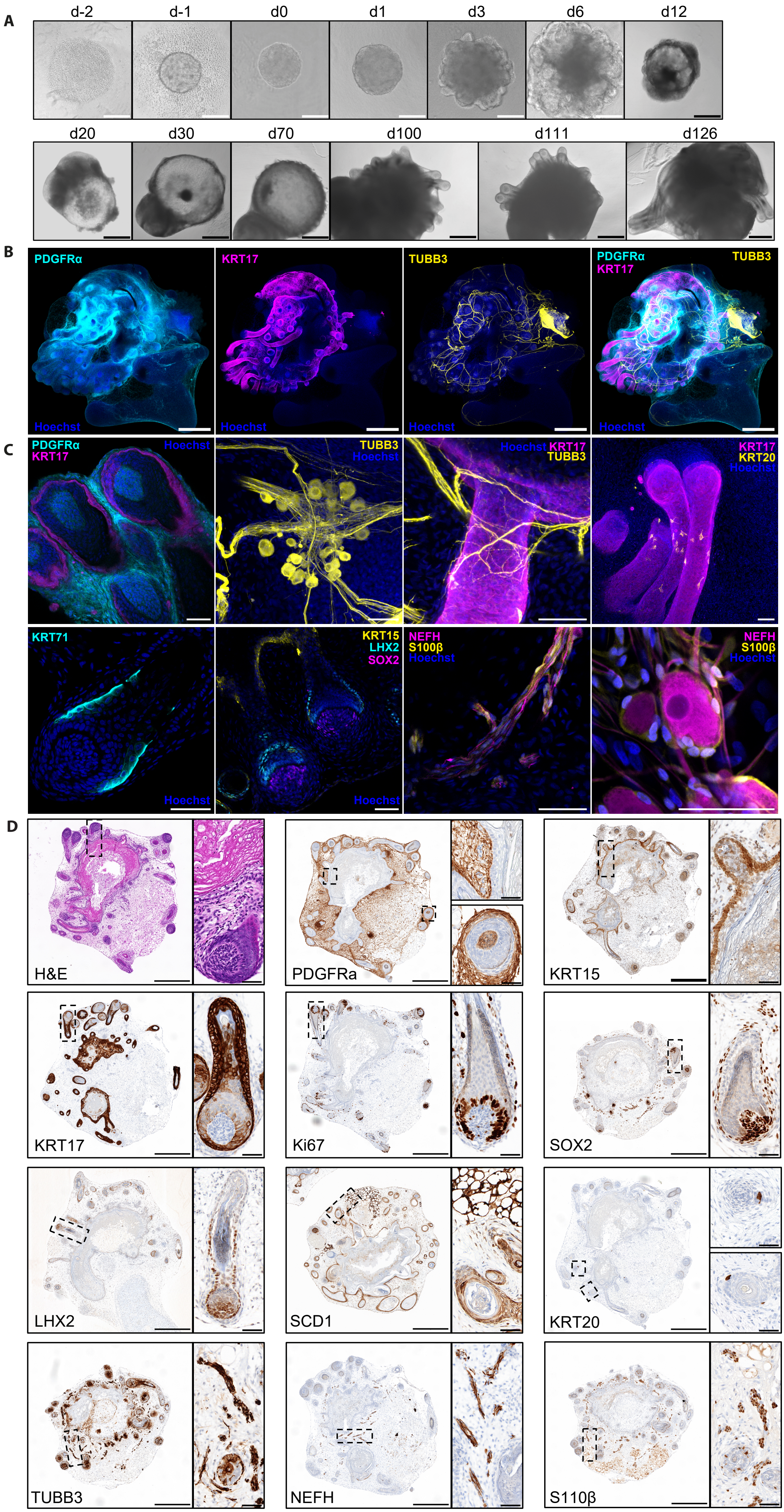

Fig. S1. Characterization of skin organoid (SkO) differentiation. (**A**) Brightfield images of SkOs from day -2 (d-2) of differentiation to 126 days (d126). White scale bars represent 200µm, black scale bars 500µm. (**B**) Maximum intensity projections from Z-stacks of overview images from WMI staining of SkOs at 120 days of differentiation. Scale bars 500µm. (**C**) Zoom-ins of WMI staining. Scale bars 50µm. (**D**) IHC staining of SkOs at 120 days of differentiation. Boxes in overview images indicate areas of higher magnification shown at the right. Scale bars represent 500µm in overview images and 50µm in zoom-ins. H&E: hematoxylin and eosin stain. Hoechst: nuclear stain. Ki67: proliferating cells. KRT15: basal layer of epidermis, outer root sheath, medulla. KRT17: epidermis, outer root sheath of HFs. KRT20: Merkel cells. KRT71: inner root sheath of HFs. LHX2: bulge region, hair germs, hair pegs, hair placodes. NEFH: sensory neurons, large soma neurons. PDGFRα: dermal fibroblasts, dermal papilla. S100β: Schwann cells, satellite glial cells, adipocytes, melanocytes. SCD1: adipocytes, sebaceous glands. SOX2: dermal condensates, dermal papilla, melanocytes, Merkel cells. TUBB3: neuronal cells, Schwann cells.

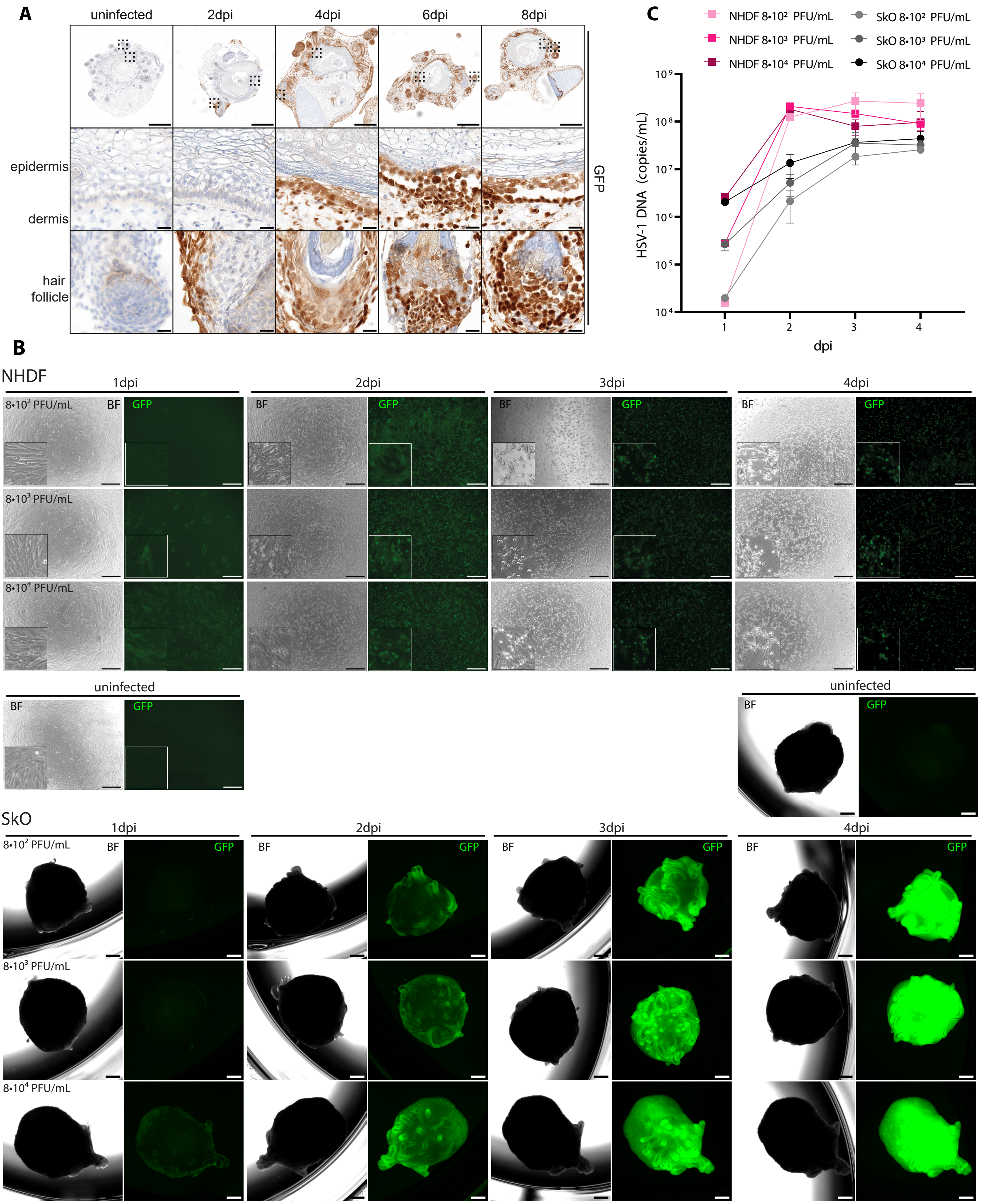

Fig. S2. Spread of HSV-1 infection in SkOs and NHDF cells. (**A**) IHC staining against GFP of 120 days old SkOs infected with HSV-1 expressing GFP from CMV-IE promoter. Boxes in overview images indicate areas of higher magnification shown below. Scale bars 500μm in overview images and 20μm in zoom-ins. Upper picture of zoom-ins shows epidermis on the top and dermis below. Lower picture in zoom-ins shows hair follicle. **(B)** Representative brightfield (BF) and fluorescence (GFP) images of NHDF cells and SkOs infected with different PFU of HSV-1 17 CMV-IEproEGFP. Uninfected and HSV-1-infected NHDF cells and SkOs are shown at 1, 2, 3, and 4 days post infection (dpi). Boxes in the left bottom corner of NHDF show zoom-ins on individual cells. Scale bars: 500 μm. (**C**) Quantification of HSV-1 DNA in the culture supernatants of infected NHDF cells and SkOs shown in (B) by qPCR. Three biological replicates were analyzed for each condition (n = 3 NHDF cultures and n = 3 SkOs). Error bars represent the standard deviation (SD).

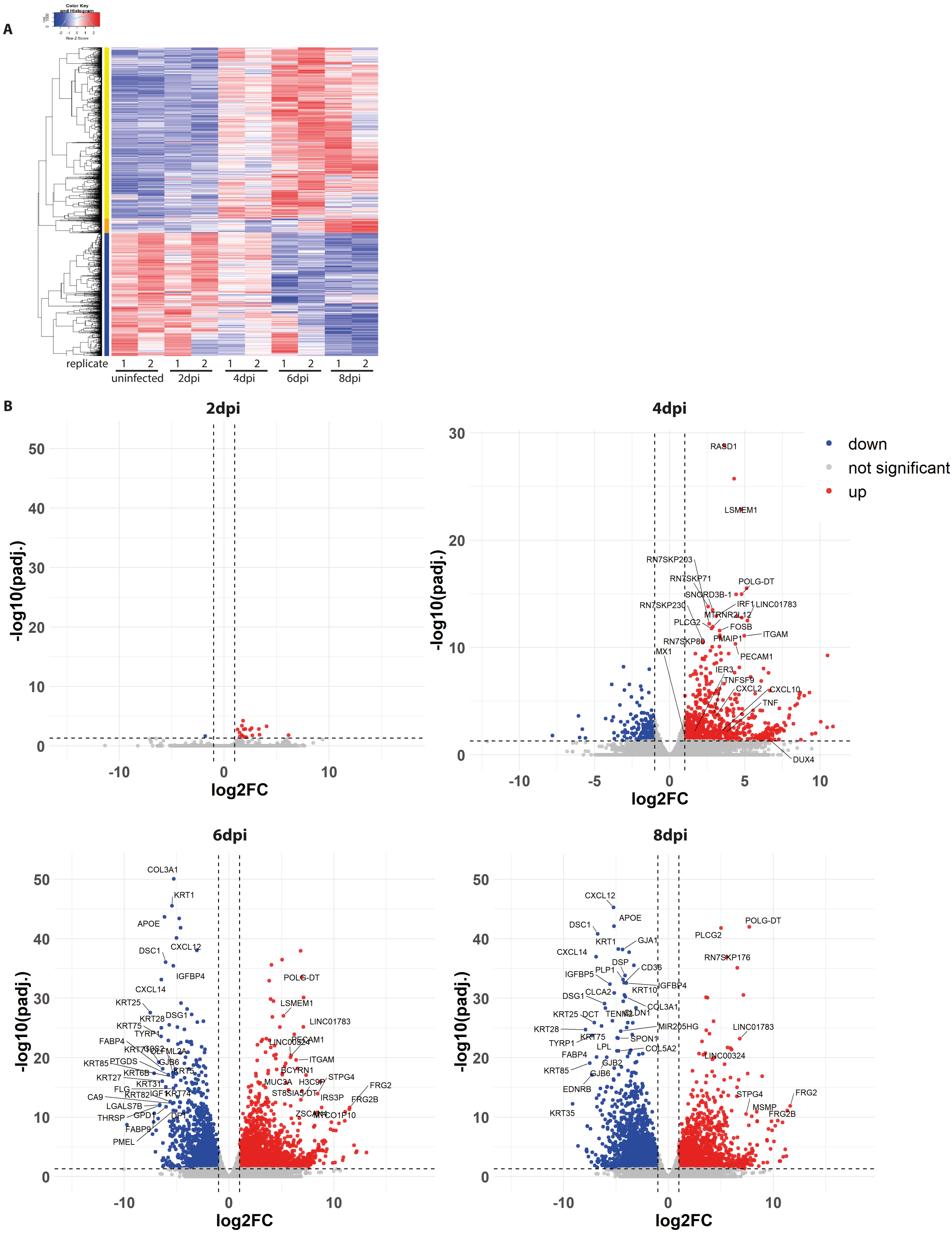

Fig. S3. (**A**) Heatmap of bulk RNA-seq of HSV-1 infected SkOs showing deregulated genes of HSV-1 infected SkOs at 2, 4, 6, and 8dpi vs uninfected SkOs in the individual replicates. (**B**) Volcano plots of bulk RNA-seq showing all differentially expressed host genes at 2, 4, 6, and 8dpi vs uninfected SkOs. All fold changes and p-values are listed in table S4.

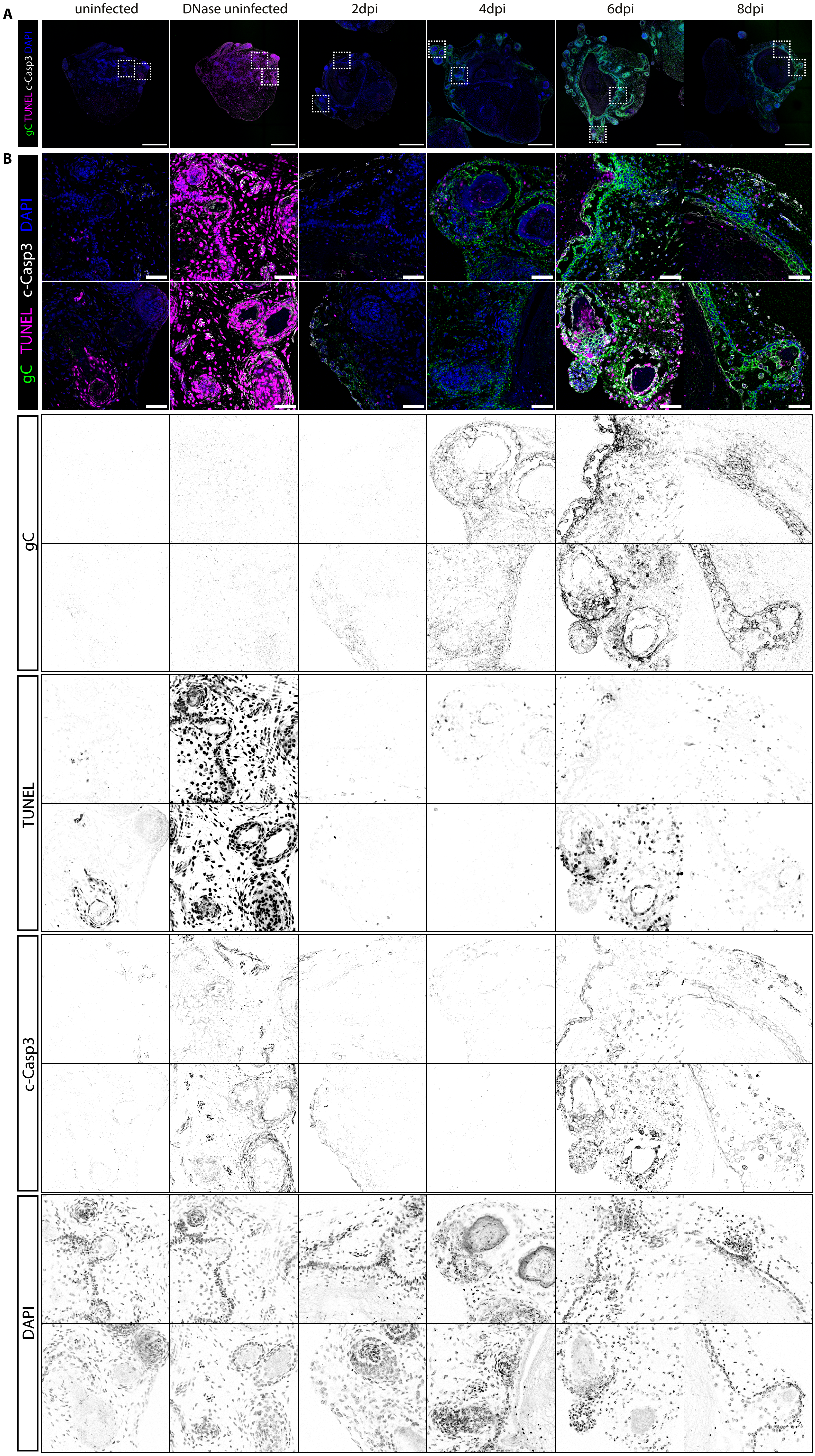

Fig. S4. Cell death in HSV-1 infected SkOs. (**A**) Overview images of TUNEL and cleaved Caspase-3 (c-Casp3) staining performed on FFPE sections of HSV-1 infected SkOs. As a positive control an uninfected tissue section was treated with DNase. White boxes indicate zoom-in areas shown in (B). Scale bars 500µm. (**B**) Zoom-ins of images shown in (A). Upper panels display merged channels, images below individual channels. Scale bars 50µm.

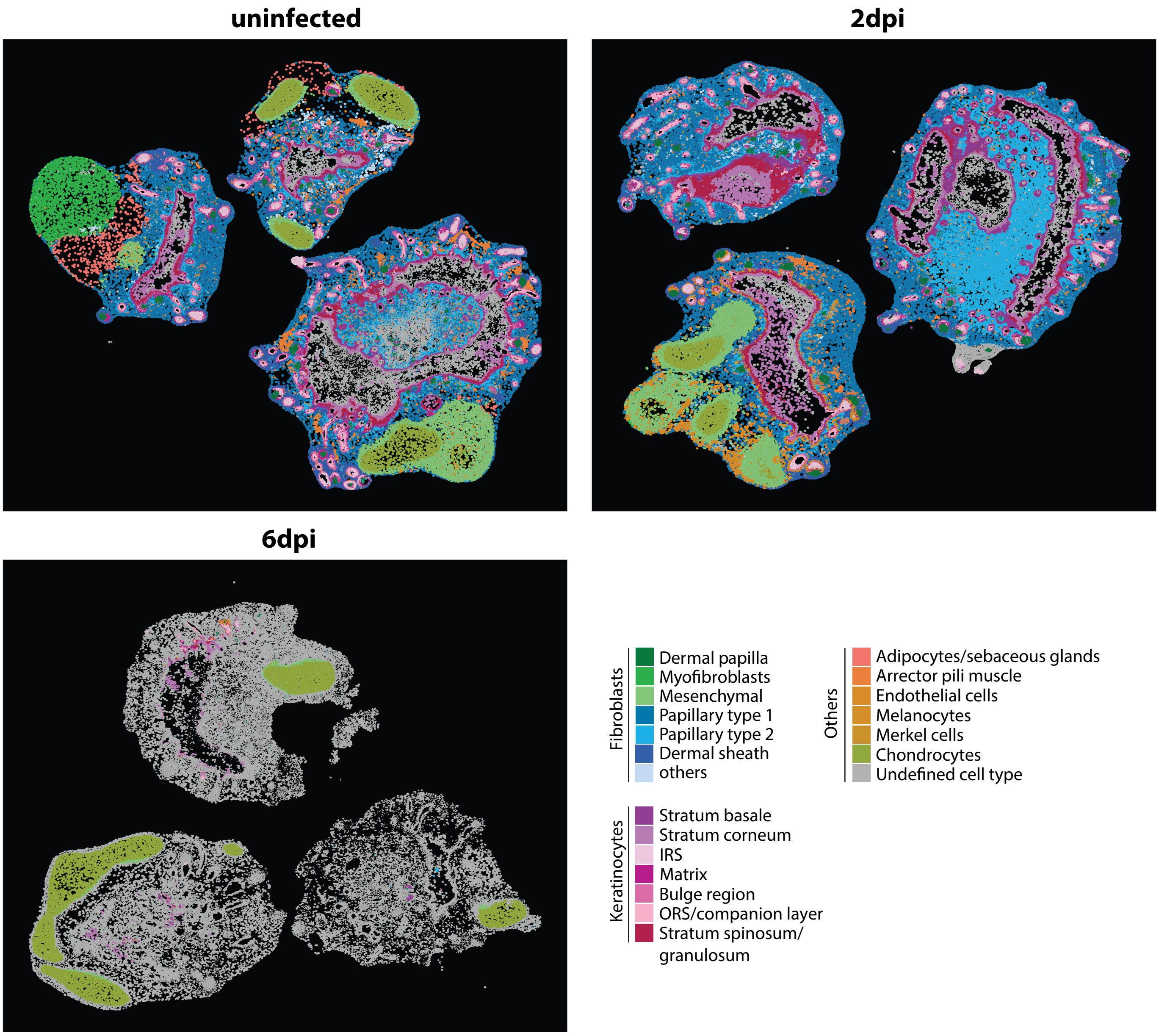

Fig. S5. Overview of all SkOs analyzed in the pilot spatial transcriptomics experiment with cells colored by cell types.

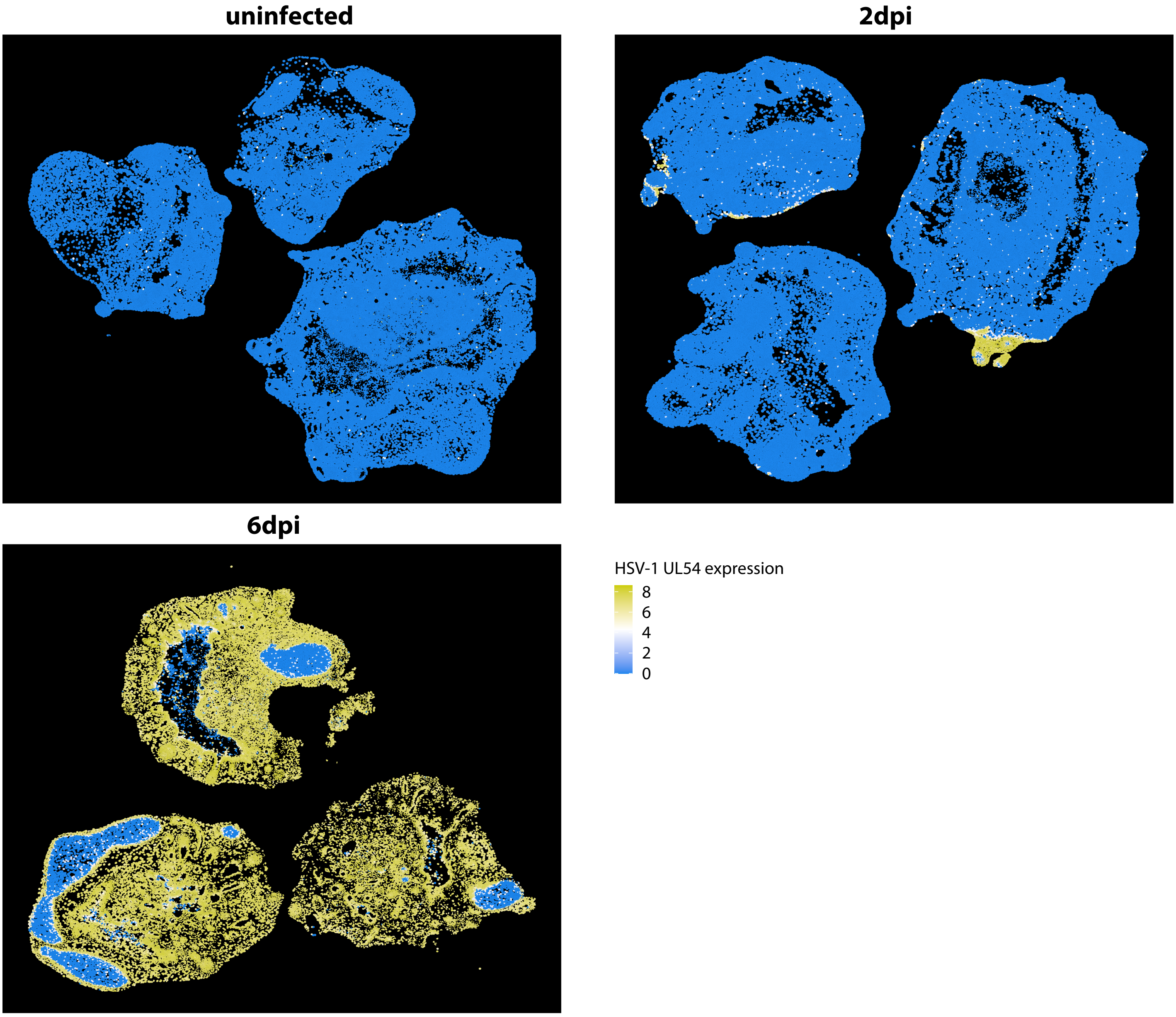

Fig. S6. Overview of all SkOs analyzed in the pilot spatial transcriptomics experiment with cells colored by HSV-1 UL54 trancript expression.

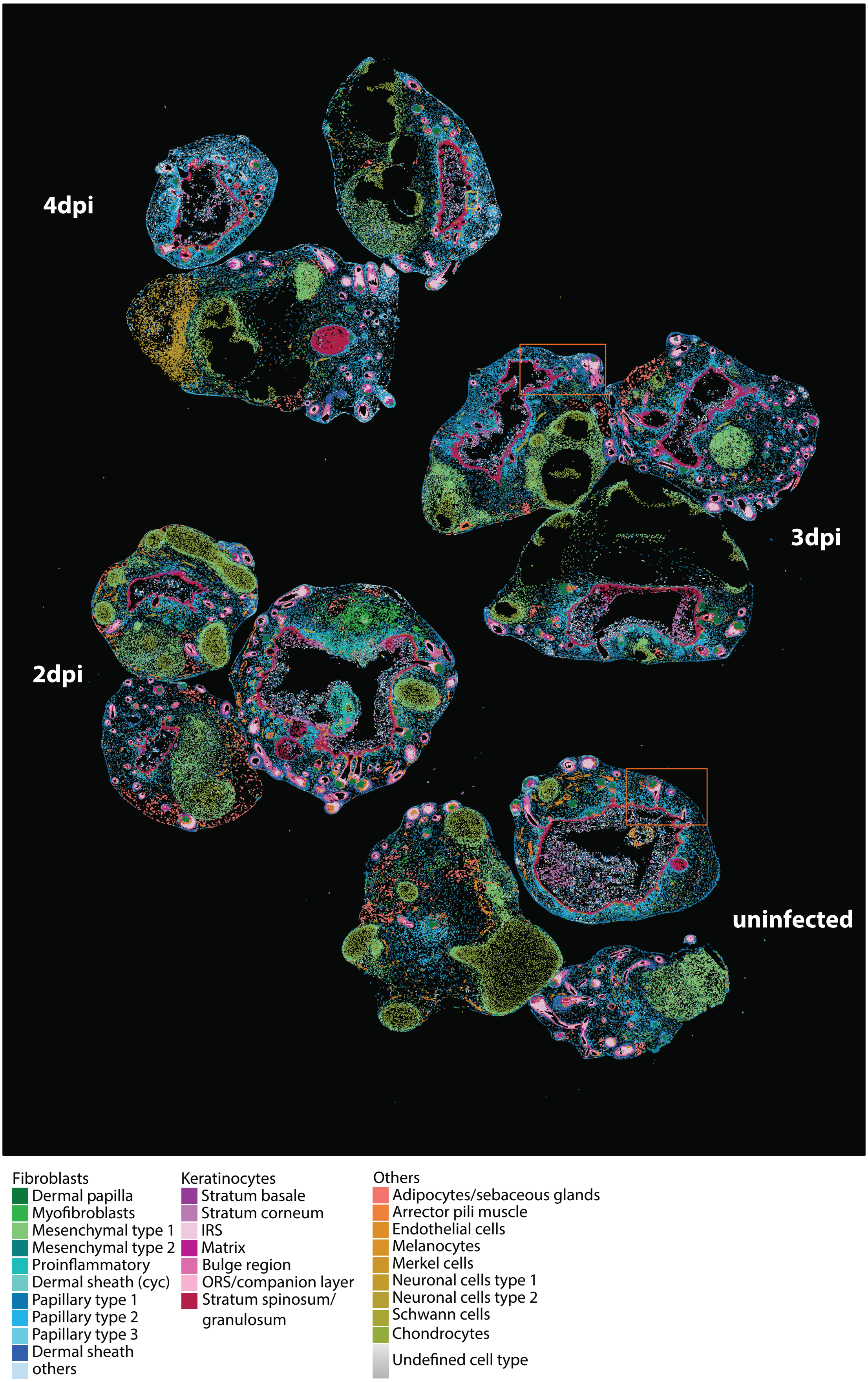

Fig. S7. Overview of all SkOs analyzed in the main spatial transcriptomics experiment with cells colored by cell types. The sections shown in Fig. 3 are highlighted by orange frames (uninfected organoid top right, 3dpi organoid top left), the part shown in fig. S19B is highlighted by an orange frame (4dpi, organoid on the right).

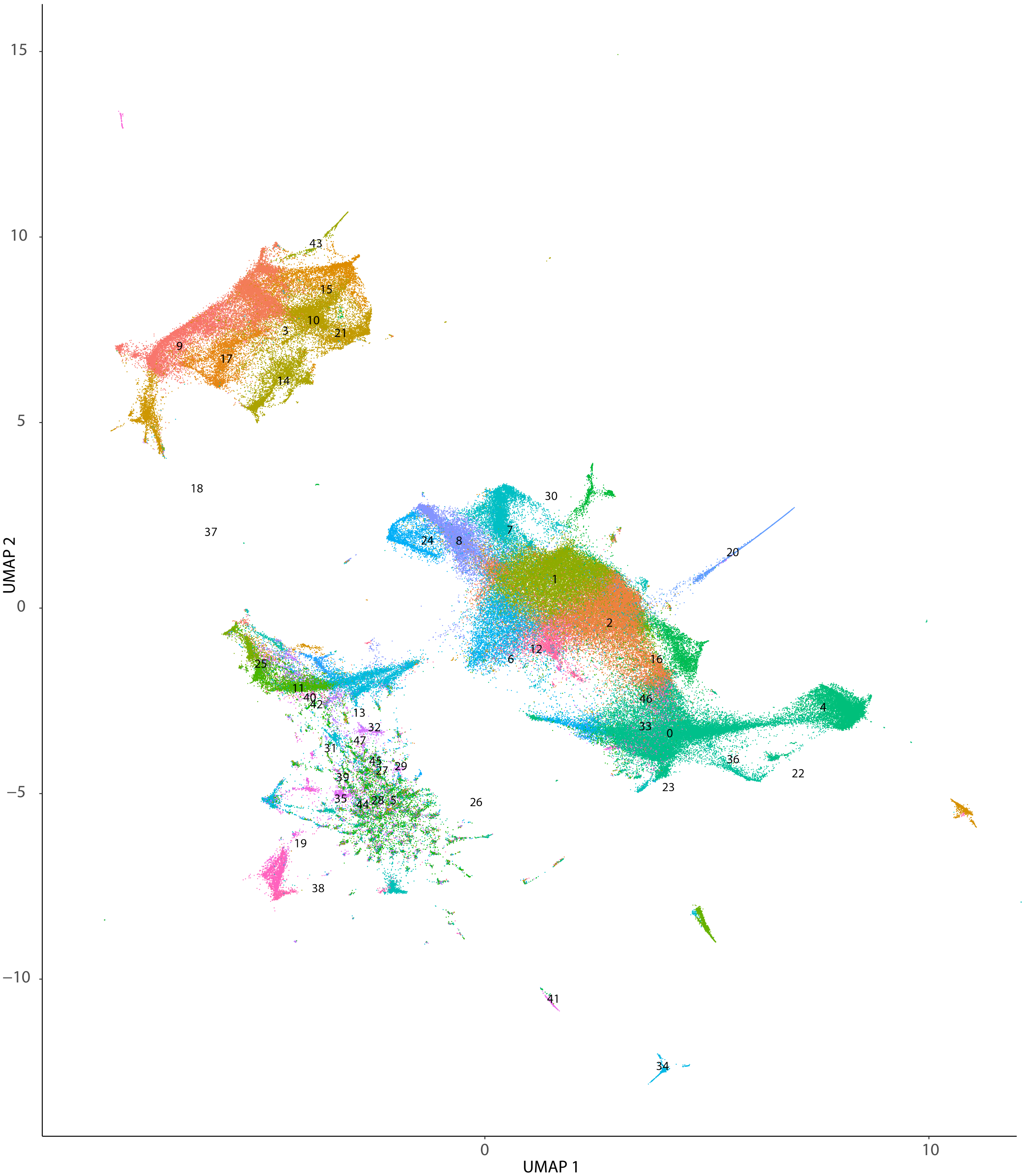

Fig. S8. UMAP representation of spatial transcriptomics data from main experiment.

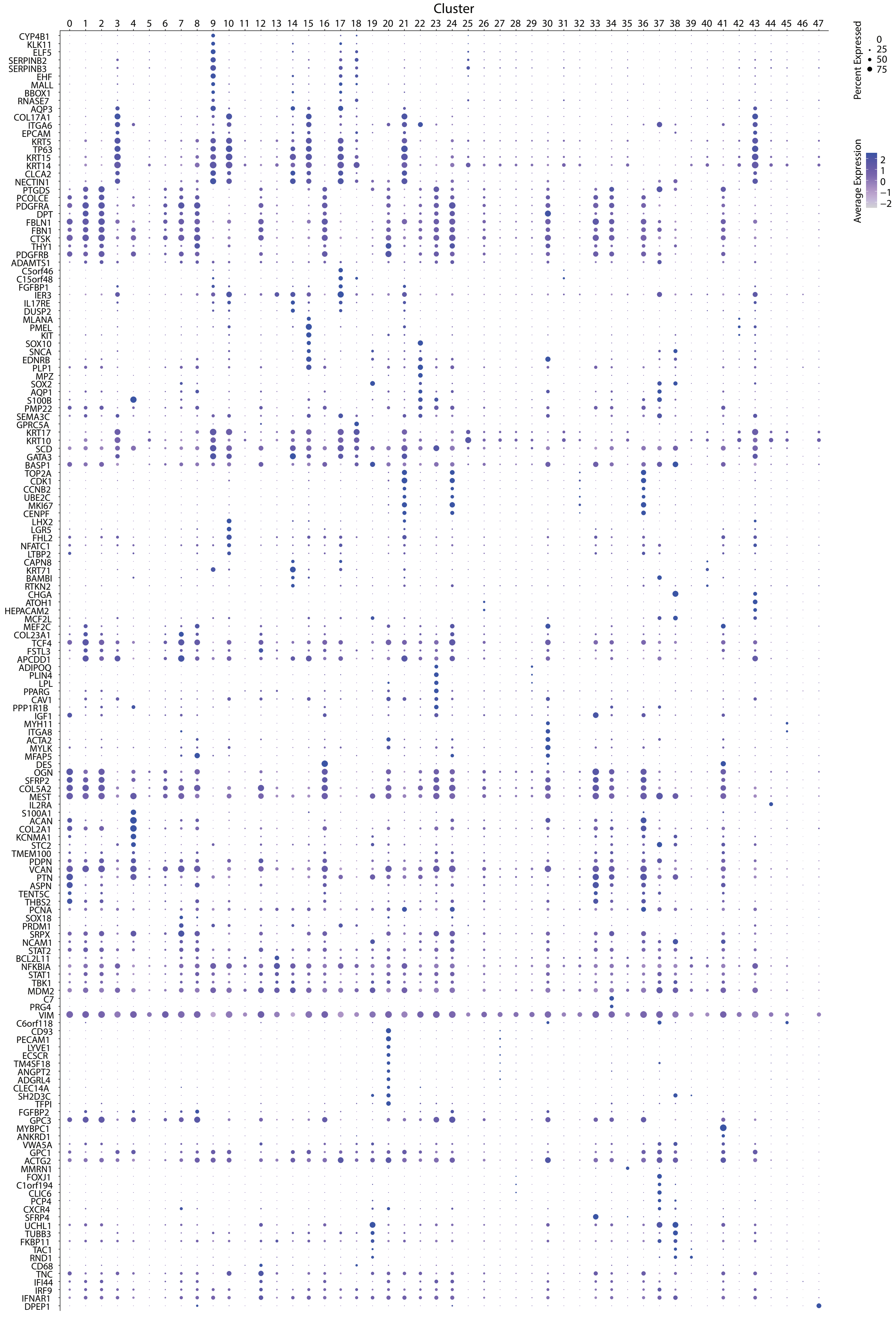

Fig. S9. Expression of marker genes in clusters from main experiment.

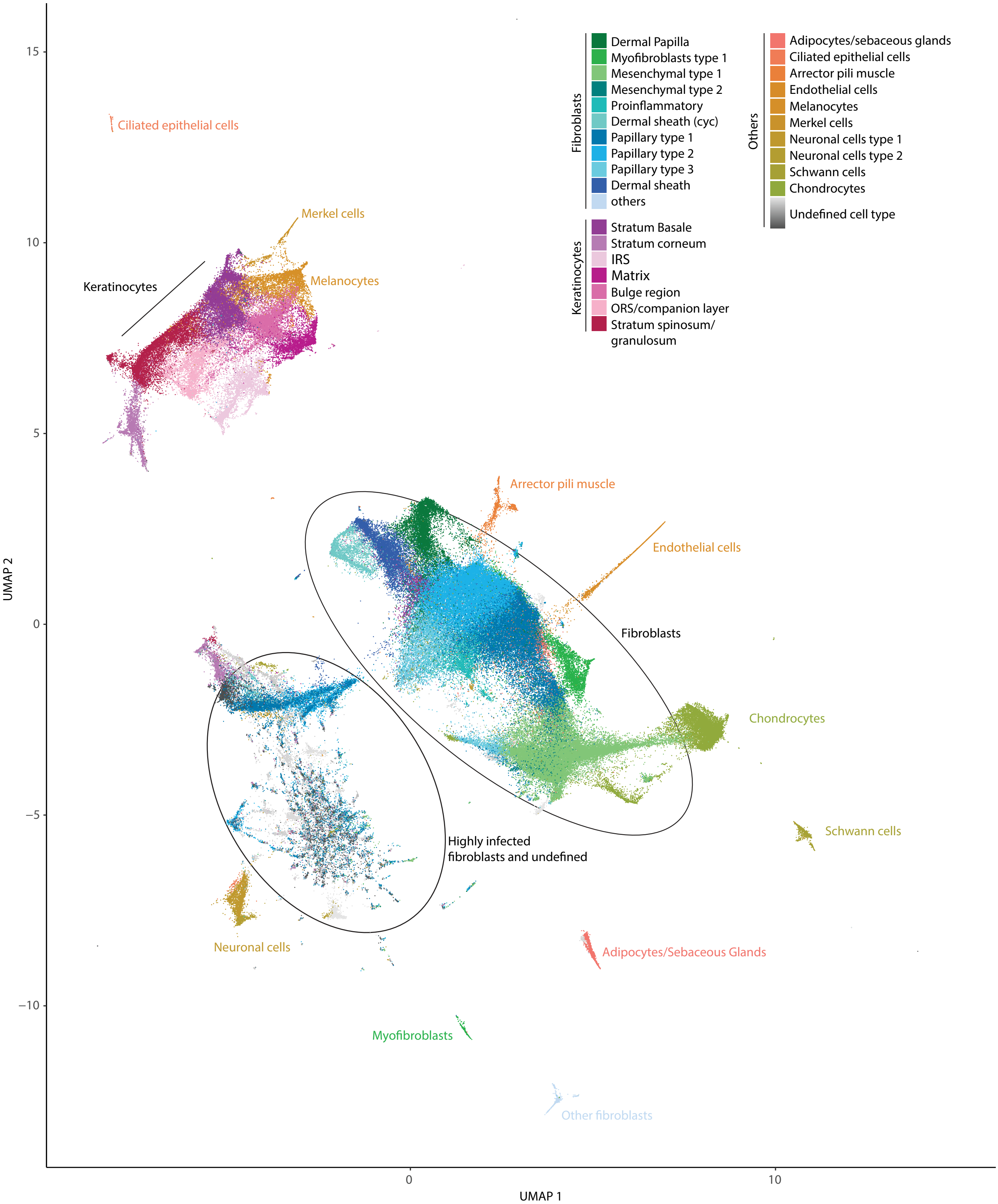

Fig. S10. UMAP representation of spatial transcriptomics data from main experiment with annotation of cell types.

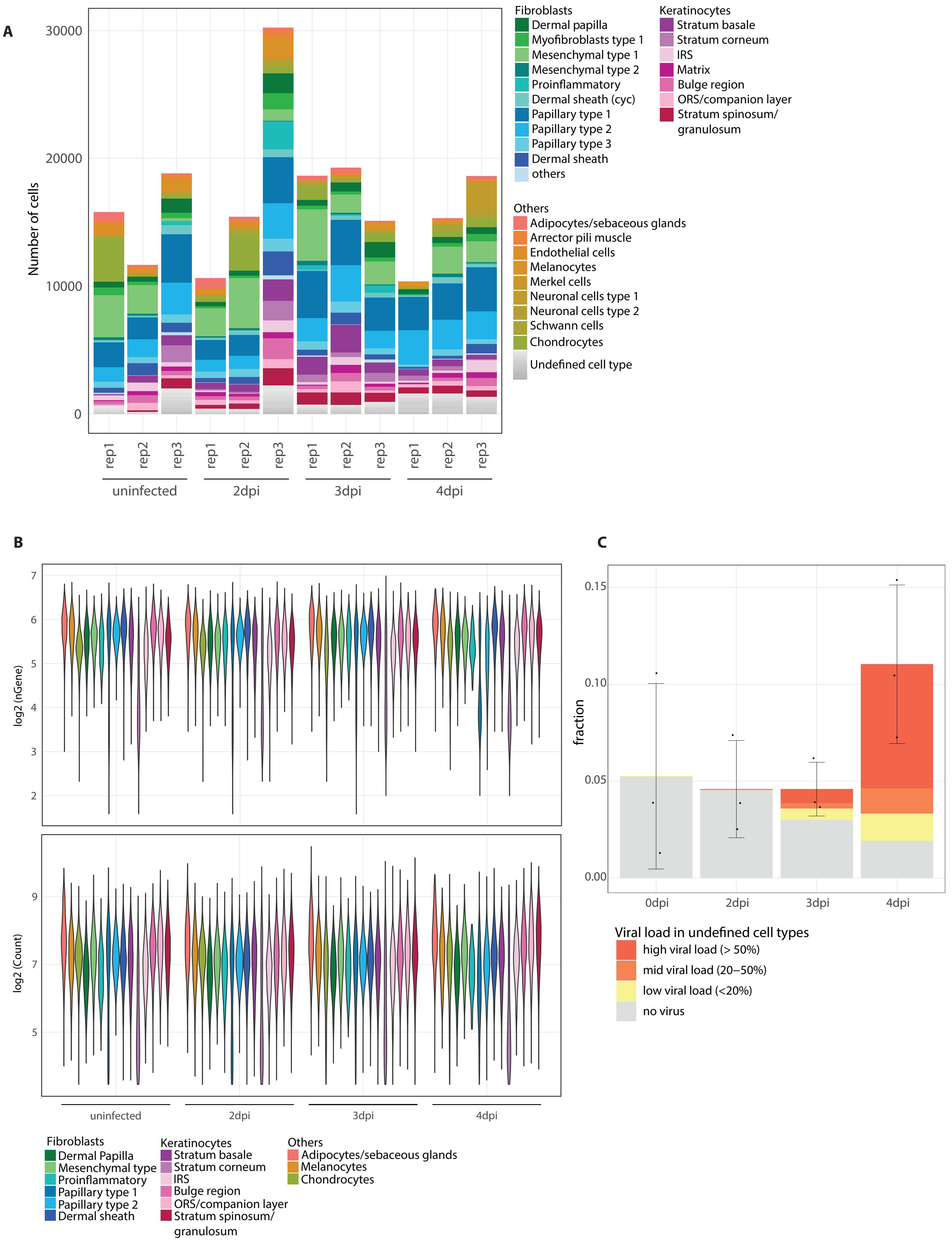

Fig. S11. Cell types and viral in gene expression spatial transcriptomics main experiment. (**A**) Number of cells in cell types per organoid replicate. (**B**) Violin plot depicting distribution of detected genes and RNA counts for the major cell types. (**C**) Percent viral transcripts in undefined cell types.

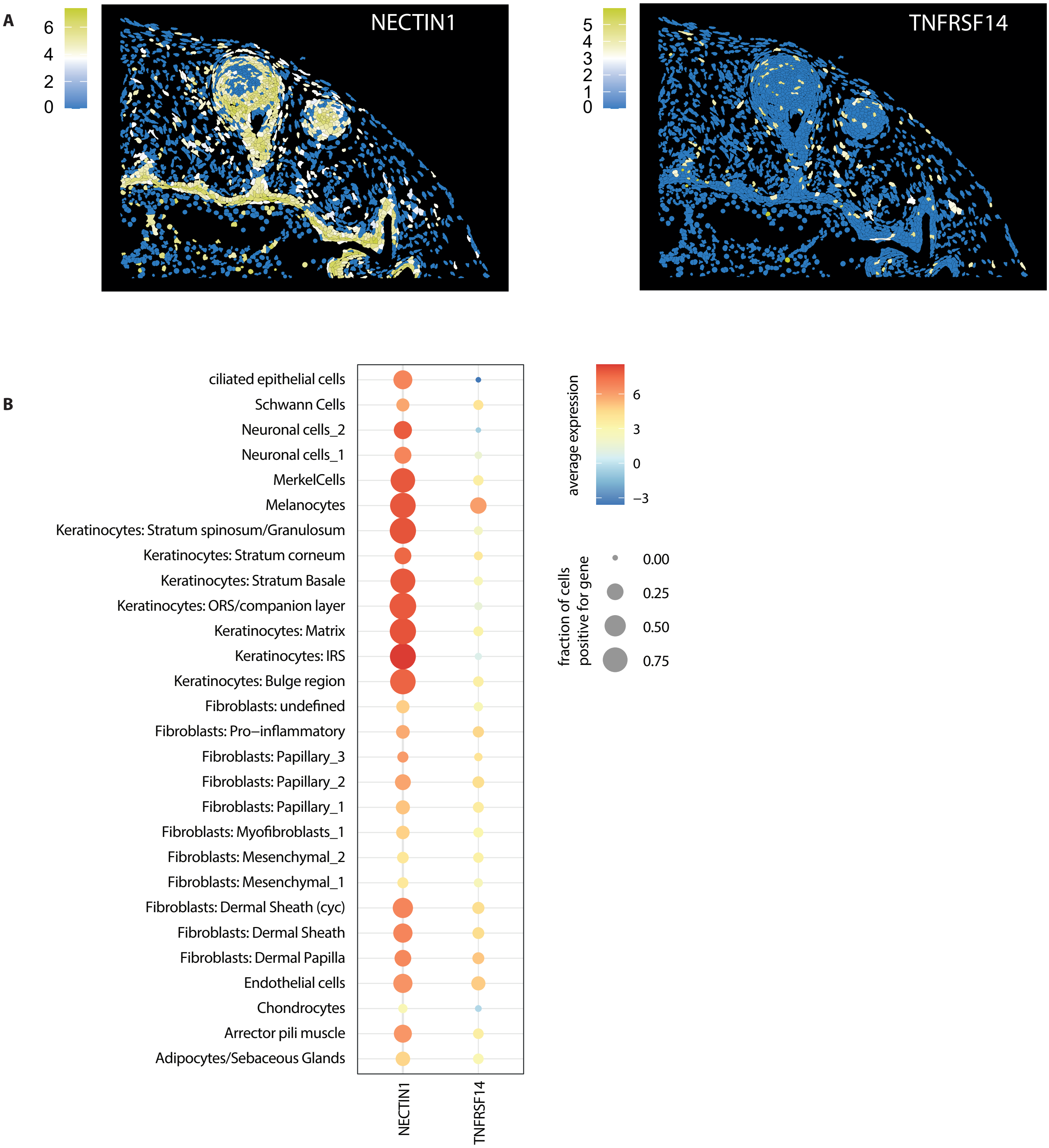

Fig. S12. Expression of the HSV-1 entry receptors Nectin-1 and TNFRSF14 (HVEM). (A) Representative region from an uninfected SkO with cells colored by expression levels of NECTIN1 and TNFRSF14. (**B**) Quantified expression of NECTIN1 and TNFRSF14 in selected cell types.

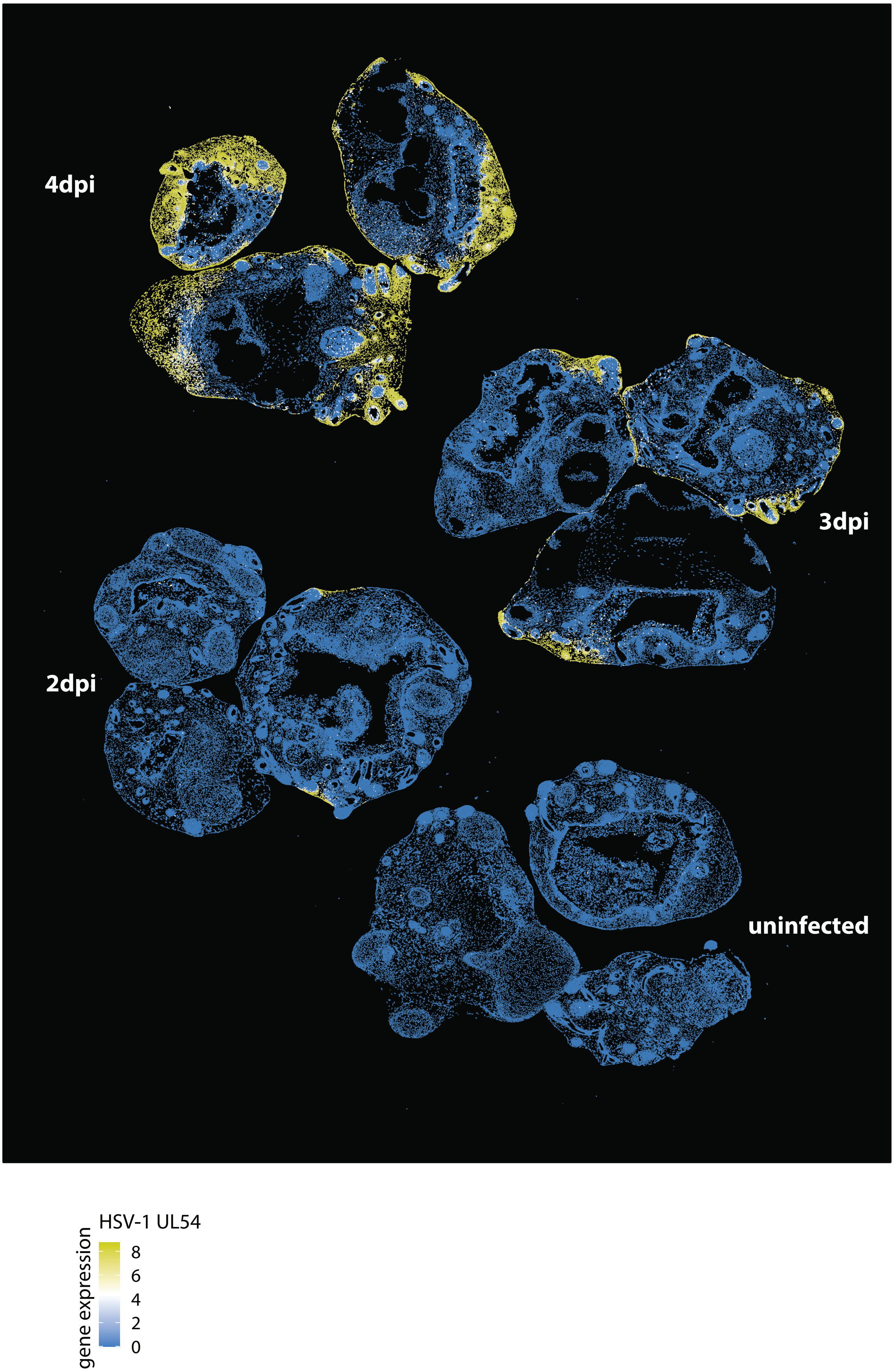

Fig. S13. Overview of all SkOs analyzed in the main spatial transcriptomics experiment with cells colored by normalized expression of HSV-1 UL54 RNA.

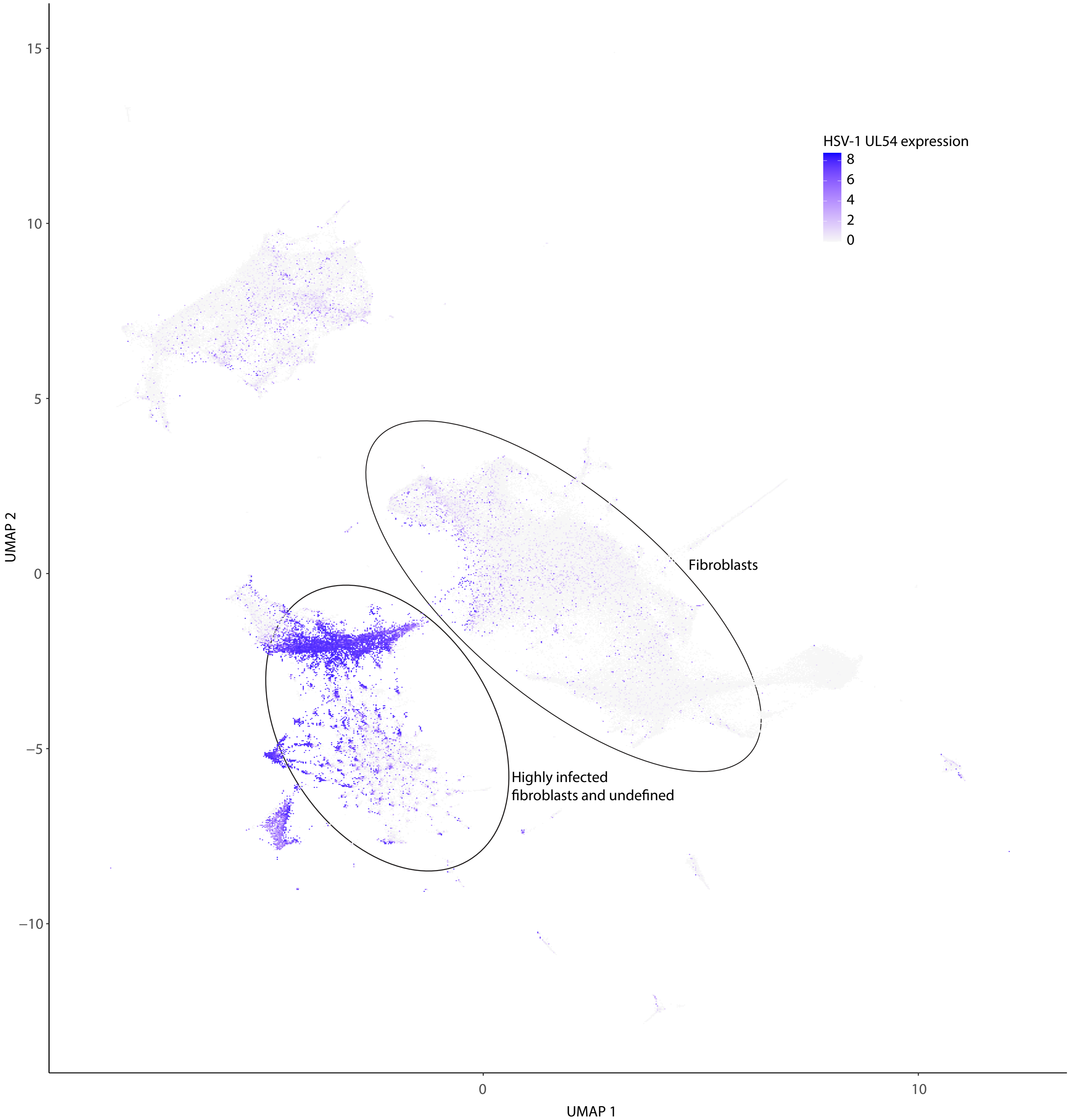

Fig. S14. UMAP representation of spatial transcriptomics data from main experiment with cells colored by normalized expression of HSV-1 UL54 RNA.

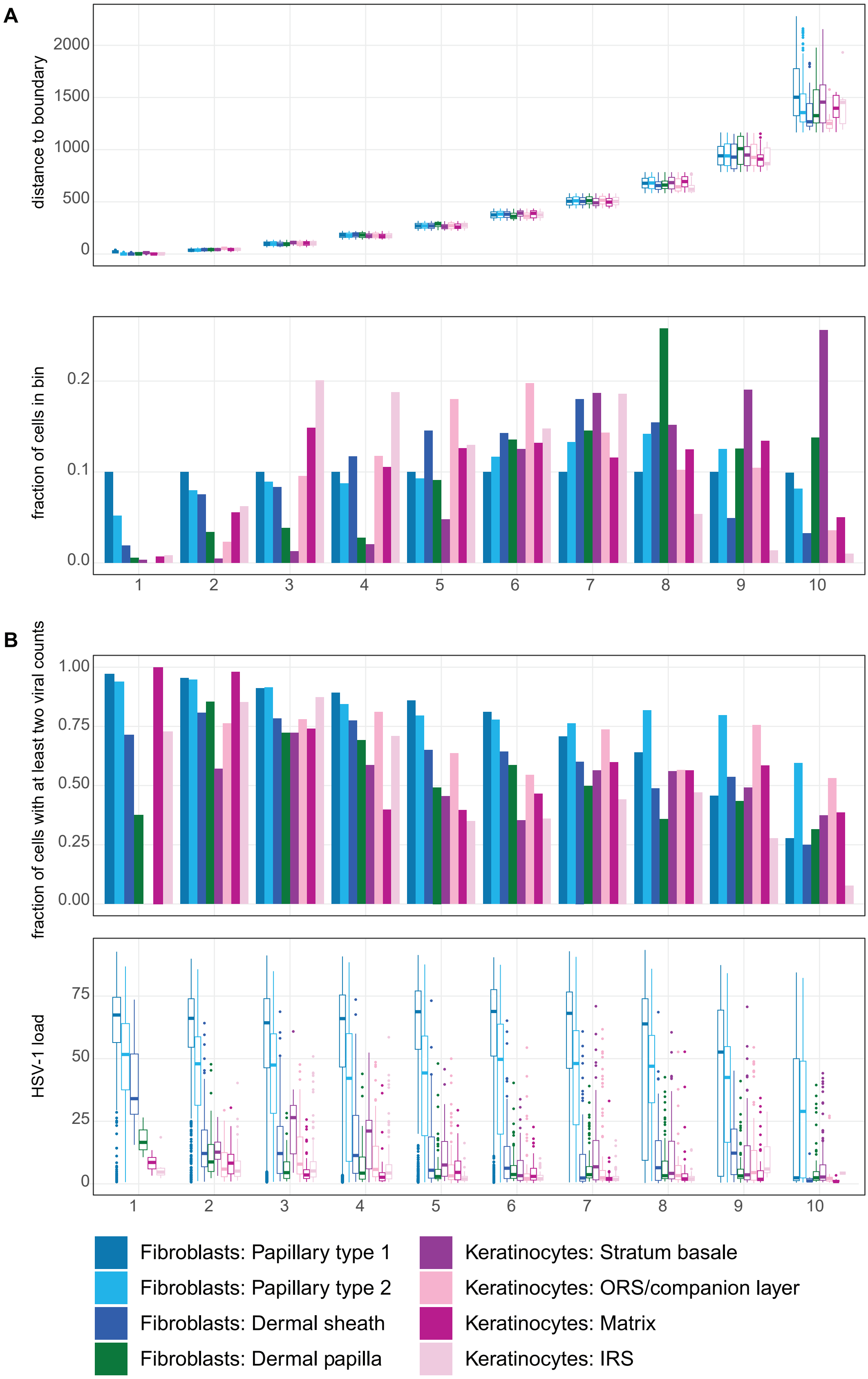

Fig. S15. Distribution of HSV-1-infected cells relative to the organoid boundary. (**A**) Papillary fibroblasts type 1 cells were binned according to their distance from the organoid boundary (µm) in 10 bins of equal size. Other cell types as indicated were sorted into the bins with the same distance range as the respective papillary fibroblasts type 1 bin. By that, the distance distribution per bin is the same for all cell types (top panel), but the fraction of cells per bin is equal only for papillary fibroblasts type 1 in all bins (bottom panel), since the other cell types in general reside further inside the organoid. (**B**) Top panel, fraction of cells with at least two viral counts per distance bin. Bottom panel, viral load (percent viral transcripts) in the different cell types per distance bins.

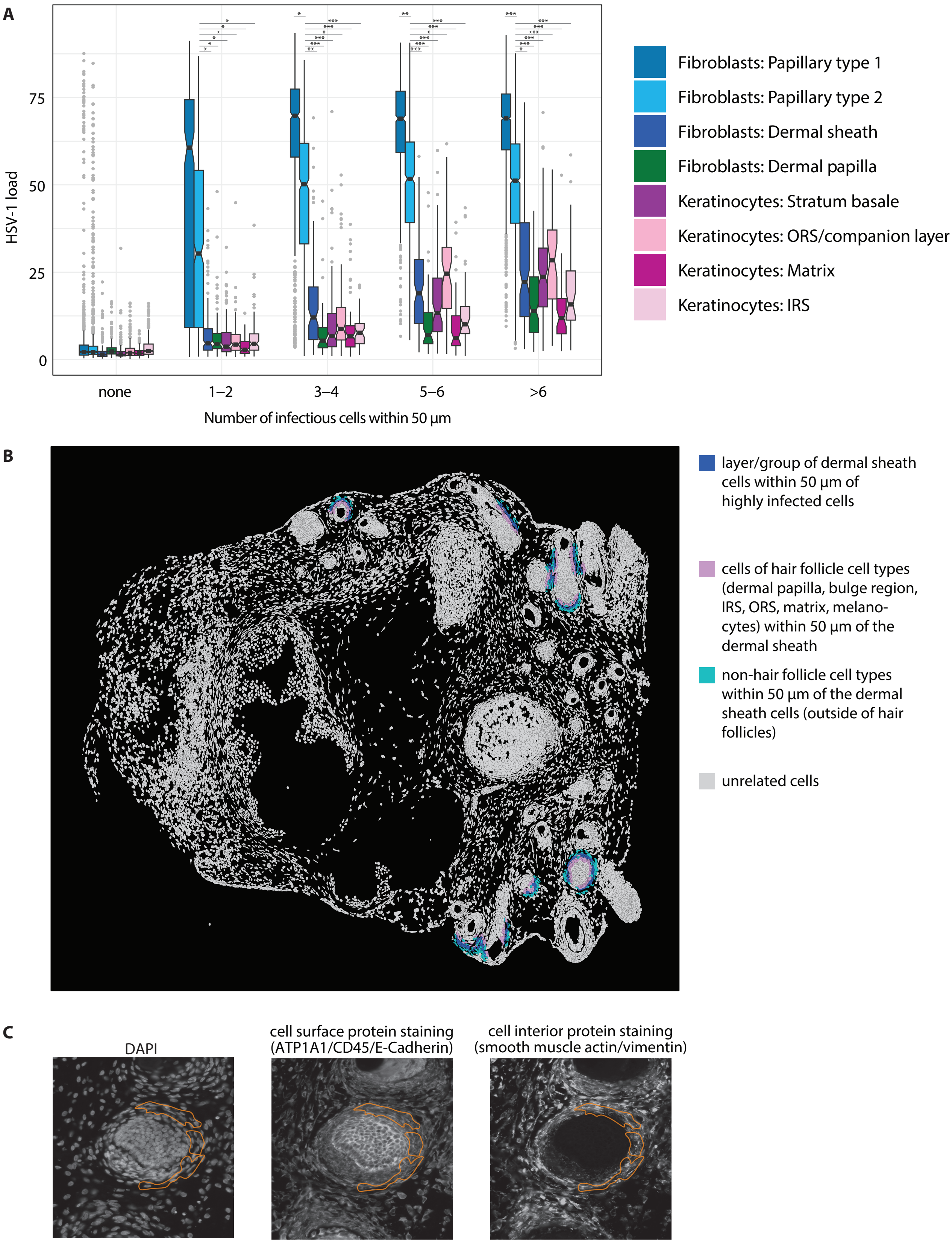

Fig. S16. Distribution of HSV-1-infected cells relative to neighboring infectious cells. (**A**) Cells from SkOs at 4dpi were binned by cell type and by the number of infectious cells (defined as >20% viral transcripts) in their neighborhood (within 50µm). Shown is the viral load (percent viral transcripts) per group as boxplots. The middle line in the boxplot displays the median, the box indicates the first and third quartile, whiskers the 1.5 interquartile range (IQR). Outliers beyond are marked by single dots. Displayed are significant changes in comparison to papillary fibroblasts type 2 determined with a generalized linear mixed-effects model (GLMM) (* padj<0.05, ** padj <0.01, *** padj <0.001). All p-values are listed in table S4. (**B**) Shown is one replicate of a HSV-1 infected SkO at 4dpi (bottom right of the 4dpi organoids in Fig. S7). Dermal sheath cells within 50µm of infectious cells (defined as >20% viral transcripts) are labelled in dark blue. Cells of hair follicle cell types (dermal papilla, bulge region, IRS, ORS, matrix, melanocytes) within 50µm of these dermal sheath cells are labelled in pink. Other cell types (i.e., non-hair follicle cell types, thus outside of HFs, such as papillary fibroblasts) are labelled in petrol. (**C**) DAPI, cell surface protein and interior protein staining on the Xenium slide. Surrounded with orange blue are dermal sheath cells as in Fig. 4D.

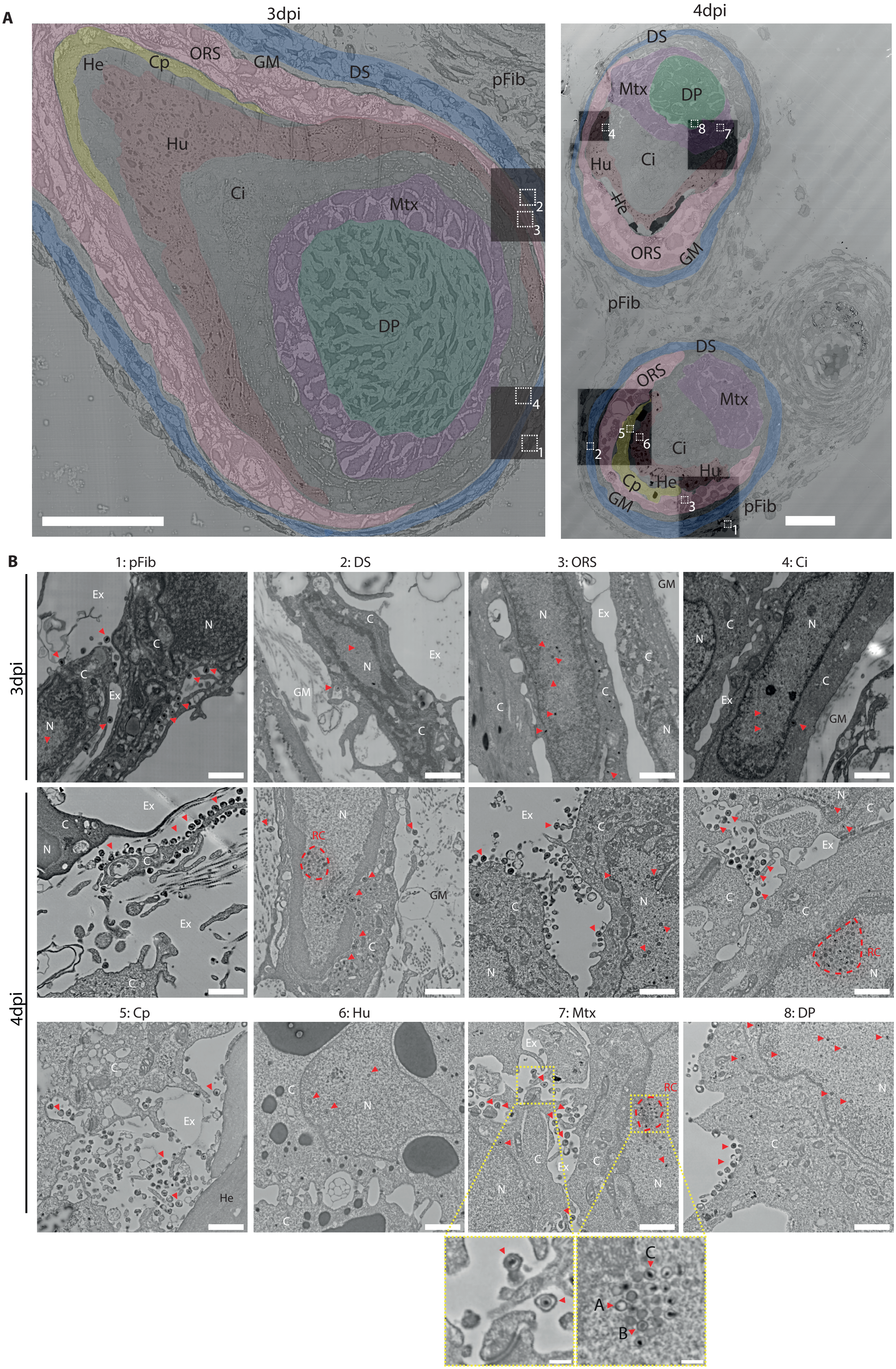

Fig. S17. Scanning electron microscopy (SEM) of HFs from HSV-1 infected SkOs at 3dpi and 4 dpi. (**A**) Overviews showing the scanned areas. Black boxes indicate areas scanned with higher resolution. White boxes with numbers indicate areas of zoom-ins shown in (B). pFib: papillary fibroblasts; DS: dermal sheath; GM: glassy membrane; ORS: outer root sheath; parts of inner root sheath (IRS): Cp: companion layer, He: Henle's layer, Hu: Huxley's layer, Ci: IRS cuticle; Mtx: matrix; DP: dermal papilla. Scale bar: 50µm. (**B**) Zoom-ins on areas indicated in (A). Red arrows indicate viral particles. Yellow boxes indicate areas of zoom-ins showing extracellular viral particles and a nuclear replication compartment with A, B and C capsids. RC: viral replication compartment; Ex: extracellular space, N: nucleus, C: cytoplasm. Scale bar: 1µm; 200nm in zoom-ins.

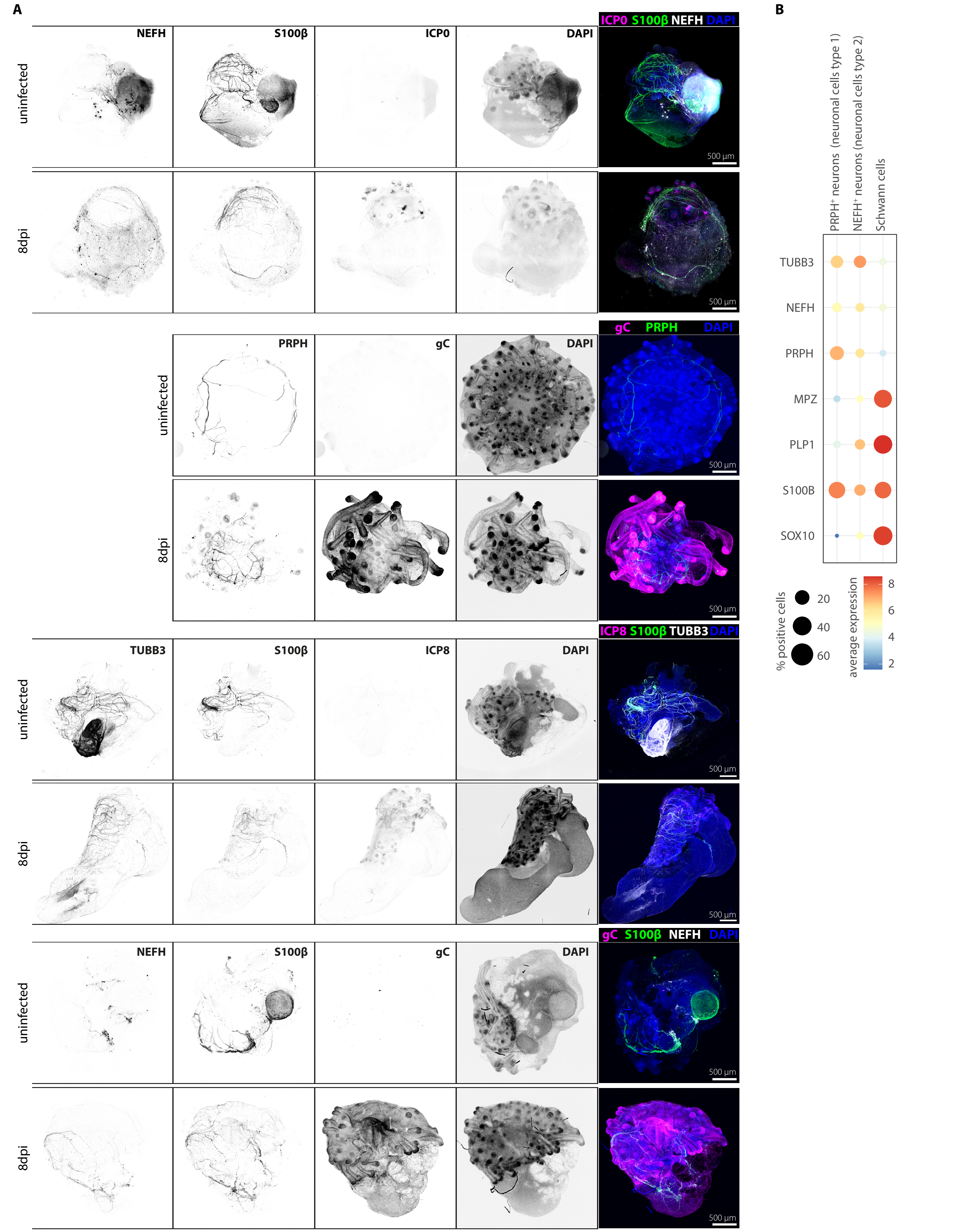

Fig. S18. (**A**) Individual fluorescence channels of overview WMI staining shown in Fig. 5. Maximum intensity projections of Z-stacks showing WMI staining of TUBB3 (neurons), S100β (Schwann cells, satellite glial cells), NEFH (sensory neurons), PRPH (sensory neurons), and HSV-1 ICP8, ICP0 and gC in uninfected SkOs and at 8dpi. (**B**) Expression of the indicated genes in Schwann and neuronal cells. Dot sizes represent the fraction of cells having at least one count of the specified gene, color the average expression.

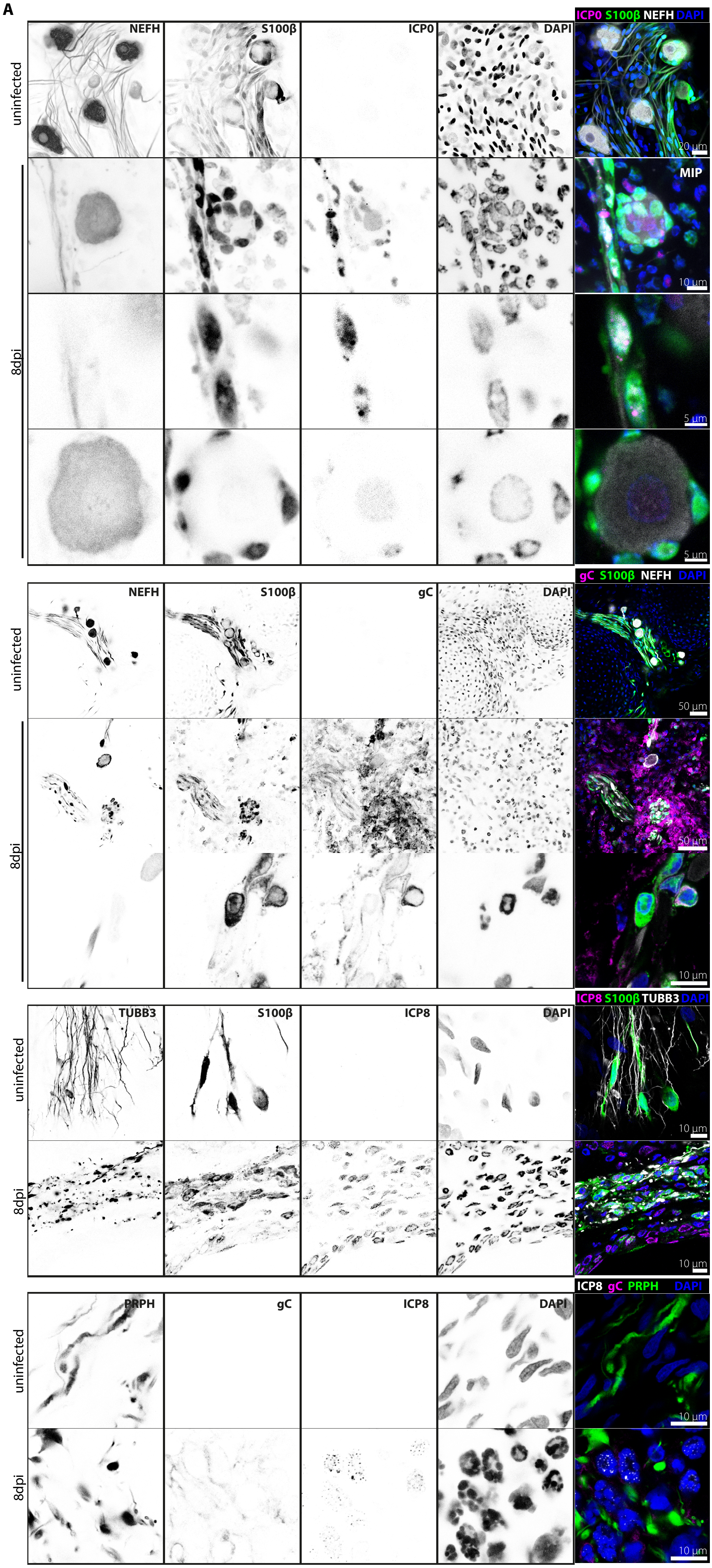

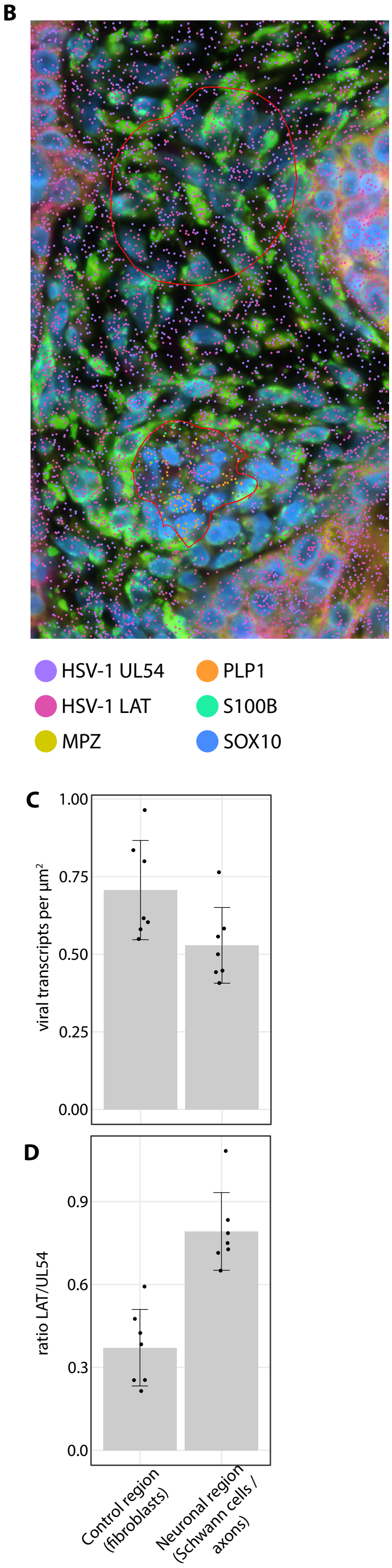

Fig. S19. (A) individual fluorescence channels of zoom-ins from whole-mount immunofluorescence (WMI) staining shown in Fig. 5. WMI staining shows TUBB3 (neurons), S100β (Schwann cells, satellite glial cells), NEFH (sensory neurons), PRPH (sensory neurons), and HSV-1 ICP8, ICP0 and gC in uninfected SkOs and at 8dpi. MIP: maximum intensity projection of a Z-stack, the two images below show zoom-ins of two planes from the Z-stack. (**B**) Definition of Control region (upper encircled region) containing mostly fibroblasts and neuronal region (lower encircled region) containing Schwann cells and putative axons in spatial transcriptomic dataset used for comparing (**C**) the viral load and (**D**) the ratio between the LAT and UL54 trancripts.

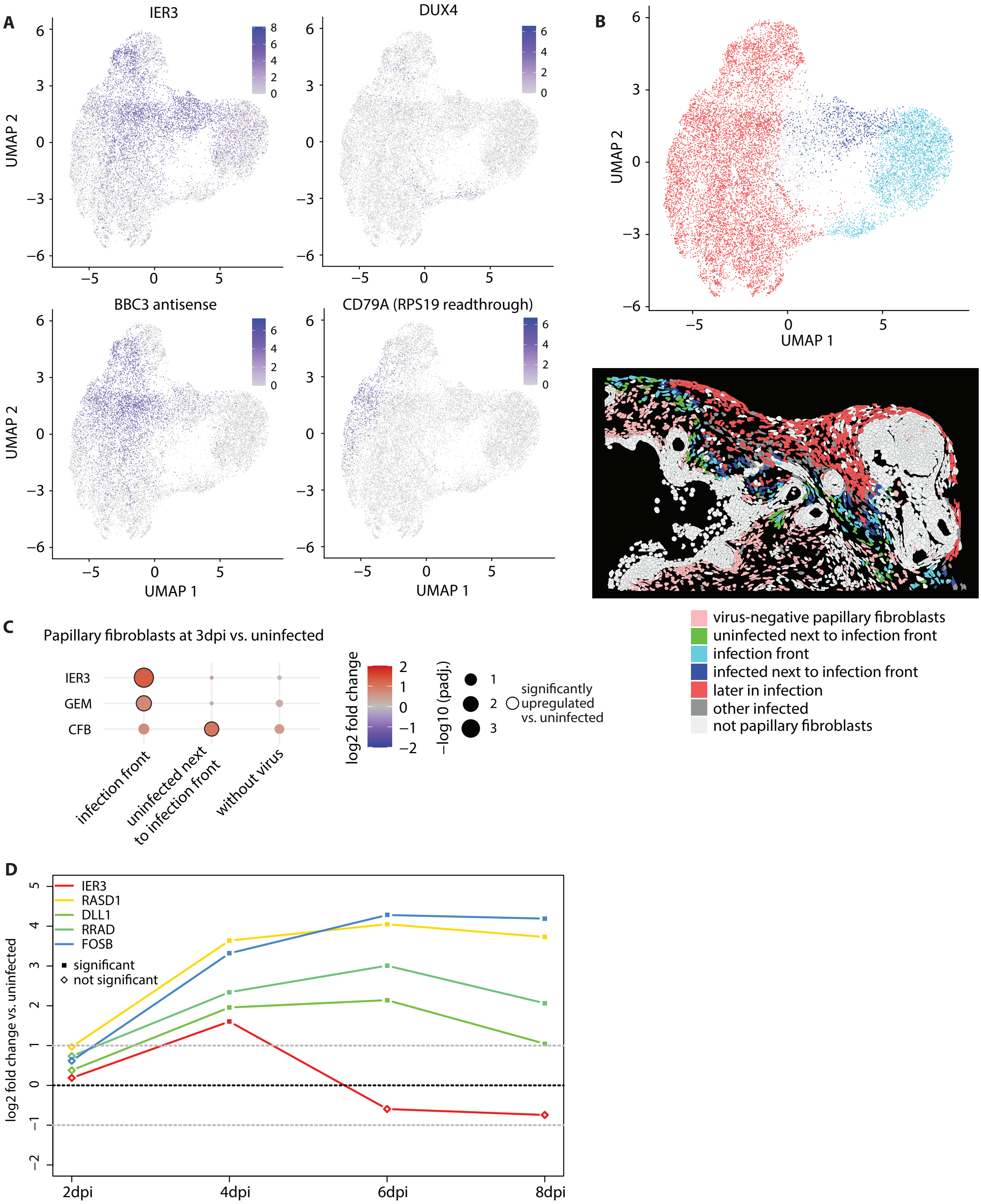

Fig. S20. Host gene expression in HSV-1 infected SkO at the infection front. (**A**) Two-dimensional projection of papillary fibroblasts as in Fig. 6A, with cells colored by expression levels of the indicated host genome transcripts. (**B**) Papillary fibroblasts were categorized in "infection front" (cluster 1 in Fig. 6AB), "uninfected next to infection front", "infected next to infection front", "later in infection" and "no virus" (i.e., without viral RNA and further away from the infection front). Top: two-dimensional projection as in (A), bottom: the same region as in Fig. 6B, both with cells colored by these categories. (**C**) Differential gene expression in papillary fibroblasts in the cell categories shown in (B) at 3dpi vs uninfected. Shown are the genes with padj < 0.05 in at least one category determined by DESeq2. All p-values are listed in table S4. (**D**) Fold changes in the bulk RNA-seq data at the indicated timepoints vs. uninfected organoids for IER3 and selected induced host genes. Significantly upregulated genes have padj <0.05 determined by DESeq2. All p-values are listed in table S4.

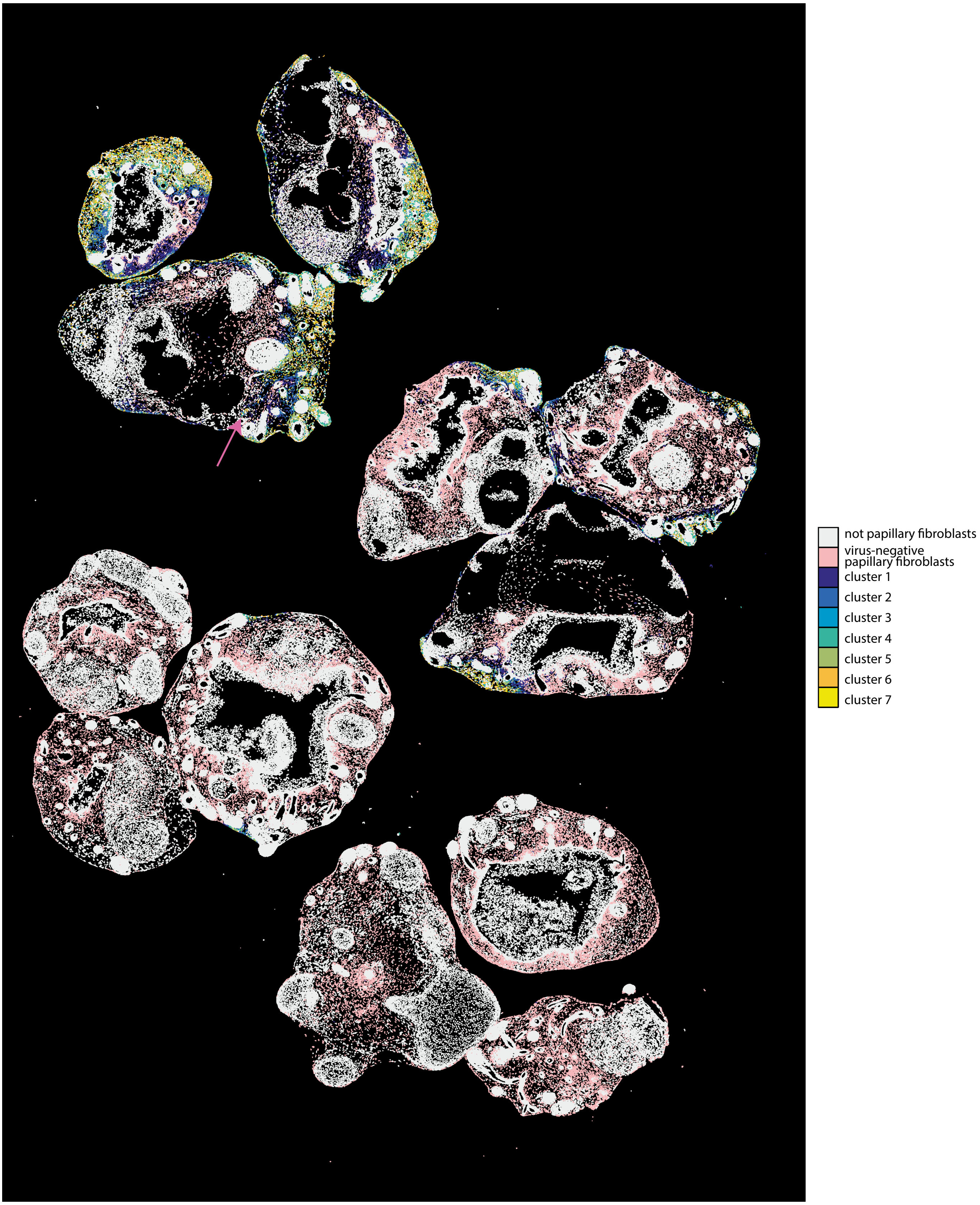

Fig. S21. Infection stage of infected papillary fibroblasts. Cells are colored by infection clusters defined in papillary fibroblasts in Fig. 6. The pink arrow at the lowest SkO at 4dpi indicates a region with cells in late stages of viral infection (clusters 5-7) which is not preceded by cells at earlier infection stages in the more inner parts of the organoid.

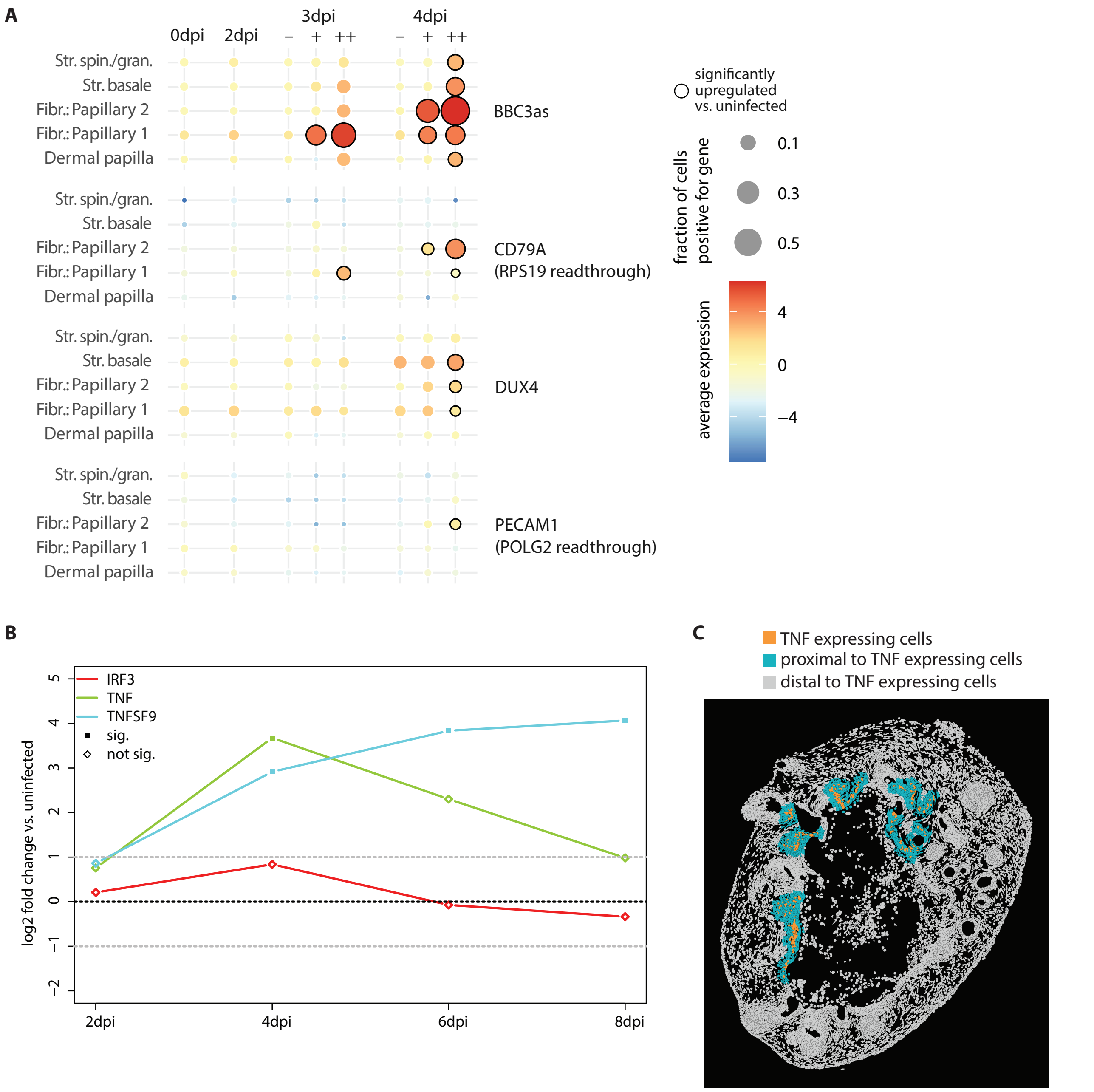

Fig. S22. Host gene expression in HSV-1 infected SkO in specific cell types. (**A**) Expression values of the indicated genes in a subset of cell types. For uninfected and 2dpi SkOs, all cells were combined for the analysis. For 3dpi and 4dpi, cells are split up in those with no or only 1 viral count (-), and those with viral load below (+) and above (++) the median. The median viral load was calculated only within cells with more than 1 count. Dot sizes represent the fraction of cells having at least one count of the specified gene, the color the average expression. Black circles around the dots indicate significant differential expression (padj <0.05) in a pseudobulk comparison vs. all cells in uninfected cells determined with DESeq2. All p-values are listed in table S4. (**B**) Fold changes in the bulk RNA-seq data at the indicated timepoints vs. uninfected organoids for IRF3, TNF and TNFSF9. Significantly upregulated genes have padj <0.05 determined by DESeq2. All p-values are listed in table S4. (**C**) Shown is one replicate of a HSV-1 infected SkO at 4dpi (top left of the 4dpi organoids in Fig. S7 and Fig. 8C). Cells are colored by TNF expressing cells, proximal and distal cells as indicated.

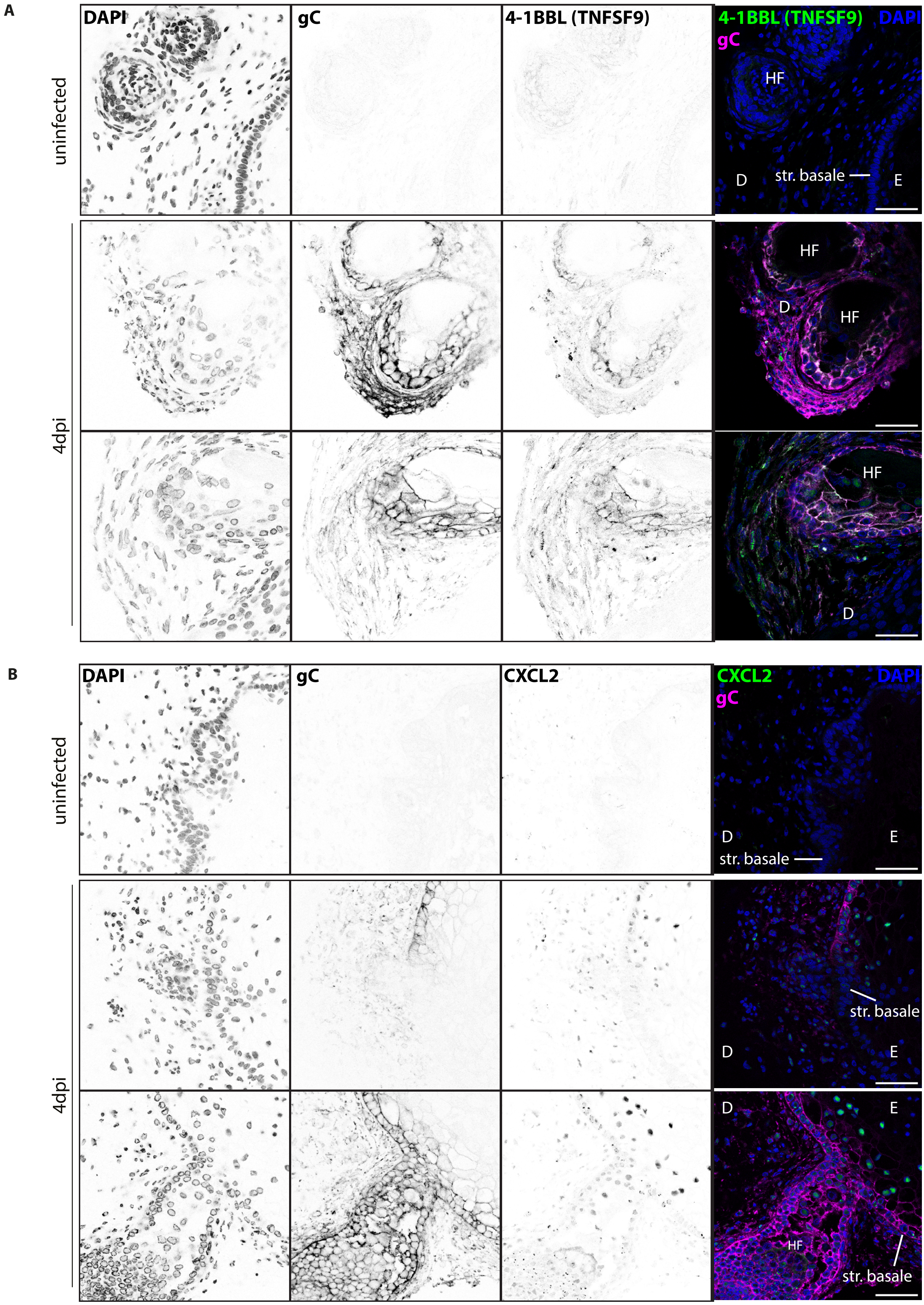

Fig. S23. IHC staining of HSV-1–infected SkOs for 4-1BBL and CXCL2 showing individual fluorescence channels. (**A**) IHC staining of HSV-1–infected SkO for 4-1BBL (TNFSF9) and HSV-1 gC, corresponding to the merged images shown in Fig. 8E, with the individual fluorescence channels displayed separately. (**B**) IHC staining of HSV-1–infected SkO for CXCL2 and HSV-1 gC, corresponding to the merged images shown in Fig. 8E, with the individual fluorescence channels displayed separately.

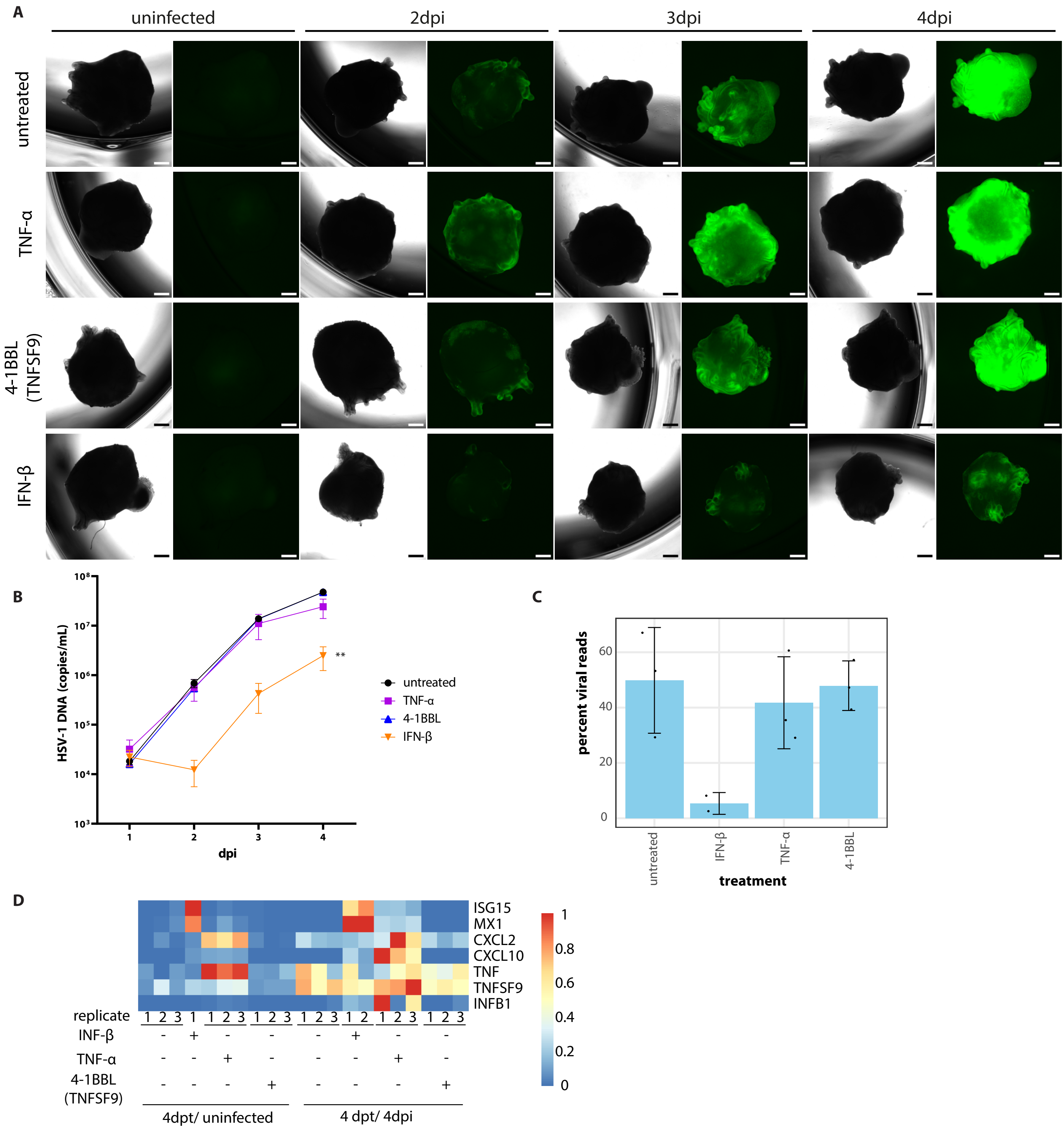

Fig. S24. Comparison of HSV-1 infection in SkOs treated with TNF-α, 4-1BBL (TNFSF9), or IFN-β. **(A)** Representative brightfield (BF) and fluorescence (GFP) images of SkOs infected with HSV-1 17 CMV-IEproEGFP and treated with the indicated factors. **(B)** Quantification of HSV-1 DNA in the culture supernatants of the infected SkOs shown in (A) by qPCR. Biological replicates were n=4 for the untreated, TNF-α-treated, and 4-1BBL-treated groups, and n=3 for the IFN-β-treated group. Error bars represent the standard error of the mean (SEM). Statistical analysis performed with aligned rank transform (ARTool package) with Holm adjustment for multiple comparisons showed a significant reduction in HSV-1 DNA levels in the culture supernatant of IFN-β-treated SkOs (** padj < 0.01). All p-values are listed in table S4. (**C**) Bulk RNA-seq analysis of HSV-1-infected SkOs at 4dpi, either untreated or treated with TNF-α, 4-1BBL (TNFSF9), or IFN-β, showing the percentage of total sequencing reads mapping to the HSV-1 genome. Biological replicates were n=3 for the untreated, TNF-α-treated, and 4-1BBL-treated groups, and n=2 for the IFN-β-treated group. (**D**) Heatmap of the bulk RNA-seq analysis shown in (C), displaying the relative expression of selected genes across individual biological replicates.

Table S1. Cell type annotation of cell clusters from main and pilot spatial transcriptomics experiment

Cell type	Key markers (positive)	Key markers (negative, if relevant)	Spatial localization	Notes	Cluster # main exp	Cluster # pilot exp
Adipocytes/ sebaceous glands	ADIPOQ, PLIN4, LPL, PPP1R1B		large clusters of cells in dermis and associated with hair follicles	Adipocytes and sebaceous glands are not distinguishable based on markers alone, only by localization	23	38
Arrector pili muscle	MEF2C, EDNRB, MYH11, ITGA8, MYLK, ACTA2		cells in strands, associated with hair follicles		30	16
Chondrocytes	ACAN, COL2A1		tail		4; 36	7; 55
Ciliated epithelial cells	CLIC6, SCB2A1, C1orf194, FOXJ1, PCB4, DNAAF1, CCDC39, C6orf118, CFAP53		single structure with lumen in one HSV-1 infected SkO at 3dpi	probably an off-target differentiation	37	not found
Endothelial cells	CD34, CD93, PECAM1, LYVE		strands in dermis and at outer rim		20	32
Fibroblasts: Dermal papilla	SOX2, SOX18, CXCR4		base of hair follicle		7	10
Fibroblasts: Dermal sheath	MFAP5, ACTA2		around hair follicle		8	4
Fibroblasts: Dermal sheath (cyc)	MFAP5, ACTA2, TOP2A, CDK1, MKI67, CENPF, UBE2C		around hair follicle	cycling dermal sheath cells	24	not found
Fibroblasts: Mesenchymal type 1	ASPN, GPC3	SFRP4	cells in dermis associated with chondrocytes		0	5
Fibroblasts: Mesenchymal type 2	ASPN, GPC3, SFRP4		single cells in dermis		33	not found

Fibroblasts: Myofibroblasts	DES, ASPN, SFRP2, MYBPC1, NCAM1		single cells in dermis		16; 41	25
Fibroblasts: others	GPC3, PRG4, C7, TMEM100		single cells in dermis		34	40
Fibroblasts: Papillary type 1	APCDD1, PTGDS, GPC3, PDGFRA, POLCE		outer rim of dermis	Cells of clusters 5 and 11 (main exp.) showed similar localization as cluster 2 (main exp.). High viral gene expression and downregulation of fibroblast markers led to assignment in different clusters.	2; 5; 11	0
Fibroblasts: Papillary type 2	APCDD1(high), PTGDS, GPC3, PDGFRA, POLCE, MEF2C		in dermis below epidermis, partially associated with hair follicles	Cells of clusters 13 (main exp.) and 12 (pilot exp.) showed similar localization as clusters 1 (main exp.) and 2 (pilot exp.). High viral gene expression and downregulation of fibroblast markers led to assignment in different clusters.	1;13	2;12
Fibroblasts: Papillary type 3	APCDD1, GPC3, PDGFRA	THBS2, PTGDS, POLCE	single cells in dermis		6	not found
Fibroblasts: Pro-inflammatory	PDGFRA, TNC, UCHL1, PDPN, IRF9, STAT2, IFNAR1		single cells in dermis		12	not found
Keratinocytes: Bulge region	LHX2, LGR5, KRT5, KRT15, NFATC1		outer layer at hair follicles		10	8
Keratinocytes: IRS	KRT71		inner layer at hair follicles		14	14

Keratinocytes: Matrix	LHX2, LRG5, TOP2A, CDK1, MKI67, CENPF, UBE2C		hair follicle bulb		21	17
Keratinocytes: ORS/ companion layer	KRT15, TP63, SERPINB3, C5orf46, C15orf48, FGFBP1, SEMA3C, PRDM1		outer layer at hair follicle		17	18
Keratinocytes: Stratum basale	COL17A1, KRT5, KRT15		basal layer in epidermis		3	1
Keratinocytes: Stratum corneum	LOR, SERPINB2, SERPINB3, KRT10, KRT14	COL17A1, KRT5, KRT15	higher layers in epidermis		18; 25	6
Keratinocytes: Stratum spinosum/ granulosum	COL17A1, KRT5, KRT15, SERPINB2, SERPINB3, KLK11, KRT10, LOR		above stratum basale in epidermis		9	11; 47
Melanocytes	MLANA, PMEL, KIT, SOX10		single cells in basal layer and at hair follicles		15	22
Merkel cells	ATOH1, HEPACAM2, KRT17, CHGA		single cells at hair follicles		43	37
Neuronal cells type 1	TUBB3, SOX2		tail of HSV-1 infected SkO at 4dpi		19	not found
Neuronal cells type 2	UCHL1, PTN, MST, SOX2, TUBB3, NCAM1, CHGA, MCF2L, SH2D3C		tail of HSV-1 infected SkO at 4dpi		38	not found
Schwann cells	SOX10, MPZ, S100B		covering potential axons		22	not found

Undefined	too few markers for identification		dying cells in the middle of the head and highly infected cells	Clusters contain dying cells in the middle of the head and highly infected cells with little to no host gene expression. A cell type assignment was not possible.	26-29; 31; 32; 35; 39; 40; 42; 44-47	3; 9; 13; 15; 19-21; 23; 24; 26-31; 33-36; 39; 41-46; 48-54; 56

Table S2. Custom panel CZBXK3 for Xenium Spatial transcriptomics

ACAN	IL17RE	PRAMEF13
AGA	IL22	PRPH
ANKRD1	IL25	RNASE7
AQP1	IL4	RORC
ATOH1	IRF3	RSAD1
BBC3	IRF7	RSAD2
BBC3as	IRF9	S100B
CAMP	ISL1	SARM1
CD207	ITGA6	SCD
CD38	ITGA8	SLC27A4as
COL23A1	ITGAM	SLC9A1
COL2A1	ITGAX	SOX10
DDX58	KLF18	SRSF3rt
DEFB103A	KRT10	SRSF6rt
DUX4	KRT14	STAT1
EDAR	KRT15	STAT2
GATA3	KRT17	TBK1
HSV1_LAT	KRT5	TBX21
HSV1_UL27	KRT71	TLR3
HSV1_UL29	LHX2	TMEM173
HSV1_UL54	LOR	TNF
HSV1_US1	MAVS	TNFRSF14
IDO1	MPZ	TNFSF9
IER3	MX1	TP53
IFI44	MX2	TP63
IFNA1	NCAM1	TRIM43
IFNAR1	NECTIN1	TSLP
IFNB1	NEFH	TUBB3
IFNG	NFATC1	UCHL1
IFNGR1	NFKBIA	UTP6
IFNGR2	NPNT	VIM
IL13	PLP1	ZSCAN4
IL17C	PMEL	

Table S3. Antibodies used for whole mount immunostaining (WMI) and Immunohistochemistry (IHC)

antibody	vendor	catalog #	dilution WMI	dilution IHC
c-Caspase 3	Cell Signaling	9661T	-	1:200
CXCL2	Invitrogen	PA5-122058	-	1:200
GFP	Abcam	ab290	-	1:1000
HSV-1 gC	Santa Cruz	sc-51626	1:50	1:50
HSV-1 gD	Santa Cruz	sc-21719	-	1:50
HSV-1 ICP0	Santa Cruz	sc-53070	1:50	-
HSV-1 ICP8	Abcam	ab20194	1:50	-
IER3	Abcam	ab65152	1:75	-
IRF3	Cell Signaling	11904	1:200	-
Ki67	BD Biosciences	550609	1:100	1:100
KRT 15	Santa Cruz	sc-47697	1:50	1:100
KRT 17	Santa Cruz	sc-393091	1:100	1:200
KRT 20	Cell Signaling	13063S	1:100	1:100
KRT 71	Invitrogen	PA5-83965	1:500	-
LHX2	Millipore	ABE1402	1:1000	1:750
NEFH	Cell Signaling	2836S	1:100	1:100
PDGFRα	Cell Signaling	5241	1:100	1:200
Peripherin	Abcam	ab4666	1:2500	-
S100β	Abcam	ab52642	1:200	1:200
SCD1	Sigma-Aldrich	HPA012107	-	1:50
SOX2	BD Biosciences	561469	1:100	1:100
TNFSF9 (4-1BBL)	Antibodies-online	ABIN6940973	-	1:100
TUBB3	BioLegend	801202	1:100	1:100

Table S4. Table containing all fold-changes, p-values, and adjusted p-values from the statistical analyses of the manuscript.

REFERENCES

1. C. James, M. Harfouche, N. J. Welton, K. M. Turner, L. J. Abu-Raddad, S. L. Gottlieb, K. J. Looker, Herpes simplex virus: Global infection prevalence and incidence estimates, 2016. *Bull. World Health Organ.* **98**, 315–329 (2020).

2. S. Zhu, A. Viejo-Borbolla, Pathogenesis and virulence of herpes simplex virus. *Virulence* **12**, 2670–2702 (2021).

3. A. L. Verzosa, L. A. McGeever, S. J. Bhark, T. Delgado, N. Salazar, E. L. Sanchez, Herpes simplex virus 1 infection of neuronal and non-neuronal cells elicits specific innate immune responses and immune evasion mechanisms. *Front. Immunol.* **12**, 644664 (2021).

4. A. L. Cunningham, R. J. Diefenbach, M. Miranda-Saksena, L. Bosnjak, M. Kim, C. Jones, M. W. Douglas, The cycle of human herpes simplex virus infection: Virus transport and immune control. *J. Infect. Dis.* **194** (Suppl. 1), S11–S18 (2006).

5. A. van Lint, M. Ayers, A. G. Brooks, R. M. Coles, W. R. Heath, F. R. Carbone, Herpes simplex virus-specific $CD8^+$ T cells can clear established lytic infections from skin and nerves and can partially limit the early spread of virus after cutaneous inoculation. *J. Immunol.* **172**, 392–397 (2004).

6. C. M. Kollias, R. B. Huneke, B. Wigdahl, S. R. Jennings, Animal models of herpes simplex virus immunity and pathogenesis. *J. Neurovirol.* **21**, 8–23 (2015).

7. J. R. Silva, A. H. Lopes, J. Talbot, N. T. Cecilio, M. F. Rossato, R. L. Silva, G. R. Souza, C. R. Silva, G. Lucas, B. A. Fonseca, E. Arruda, J. C. Alves-Filho, F. Q. Cunha, T. M. Cunha, Neuroimmune-glia interactions in the sensory ganglia account for the development of acute herpetic neuralgia. *J. Neurosci.* **37**, 6408–6422 (2017).

8. L. Wirtz, N. C. De La Cruz, M. Möckel, D. Knebel-Mörsdorf, Susceptibility of human and murine dermal fibroblasts to herpes simplex virus 1 in the absence and presence of extracellular matrix. *J. Virol.* **96**, e0206821 (2022).

9. E. Rahn, P. Petermann, K. Thier, W. Bloch, J. Morgner, S. A. Wickström, D. Knebel-Mörsdorf, Invasion of herpes simplex virus type 1 into murine epidermis: An ex vivo infection study. *J. Invest. Dermatol.* **135**, 3009–3016 (2015).

10. L. Wirtz, M. Möckel, D. Knebel-Mörsdorf, Invasion of herpes simplex virus 1 into murine dermis: Role of nectin-1 and herpesvirus entry mediator as cellular receptors during aging. *J. Virol.* **94**, e02046-19 (2020).

11. N. C. De La Cruz, M. Möckel, L. Wirtz, K. Sunaoglu, W. Malter, M. Zinser, D. Knebel-Mörsdorf, Ex vivo infection of human skin with herpes simplex virus 1 reveals mechanical wounds as insufficient entry portals via the skin surface. *J. Virol.* **95**, e0133821 (2021).

12. P. Tajpara, M. Mildner, R. Schmidt, M. Vierhapper, J. Matiasek, T. Popow-Kraupp, C. Schuster, A. Elbe-Bürger, A preclinical model for studying herpes simplex virus infection. *J. Invest. Dermatol.* **139**, 673–682 (2019).

13. S. Sun, L. Jin, Y. Zheng, J. Zhu, Modeling human HSV infection via a vascularized immune-competent skin-on-chip platform. *Nat. Commun.* **13**, 5481 (2022).

14. G. Andrei, J. van den Oord, P. Fiten, G. Opdenakker, C. De Wolf-Peeters, E. De Clercq, R. Snoeck, Organotypic epithelial raft cultures as a model for evaluating compounds against alphaherpesviruses. *Antimicrob. Agents Chemother.* **49**, 4671–4680 (2005).

15. J. T. S. Chu, M. M. Lamers, Organoids in virology. *npj Viruses* **2**, 5 (2024).

16. X. Su, P. Yue, J. Kong, X. Xu, Y. Zhang, W. Cao, Y. Fan, M. Liu, J. Chen, A. Liu, F. Bao, Human brain organoids as an in vitro model system of viral infectious diseases. *Front. Immunol.* **12**, 792316 (2021).

17. Q. Shen, Y. H. Zhou, Y. Q. Zhou, A prospects tool in virus research: Analyzing the applications of organoids in virus studies. *Acta Trop.* **254**, 107182 (2024).

18. J. Lee, C. C. Rabbani, H. Gao, M. R. Steinhart, B. M. Woodruff, Z. E. Pflum, A. Kim, S. Heller, Y. Liu, T. Z. Shipchandler, K. R. Koehler, Hair-bearing human skin generated entirely from pluripotent stem cells. *Nature* **582**, 399–404 (2020).

19. J. Lee, W. H. van der Valk, S. A. Serdy, C. Deakin, J. Kim, A. P. Le, K. R. Koehler, Generation and characterization of hair-bearing skin organoids from human pluripotent stem cells. *Nat. Protoc.* **17**, 1266–1305 (2022).

20. T. Hennig, L. Djakovic, L. Dölken, A. W. Whisnant, A review of the multipronged attack of herpes simplex virus 1 on the host transcriptional machinery. *Viruses* **13**, 1836 (2021).

21. A. Mukhopadhyay, S. Indra, J. Ghosh, S. Biswas, P. Palit, D. Chattopadhyay, Herpes simplex virus-mediated skin infections: Cytokines and its interplay. *Explor. Immunol.* **4**, 394–413 (2024).

22. J. Piret, G. Boivin, Immunomodulatory strategies in herpes simplex virus encephalitis. *Clin. Microbiol. Rev.* **33**, e00105-19 (2020).

23. G. Katzilieris-Petras, X. Lai, A. S. Rashidi, G. M. G. M. Verjans, L. S. Reinert, S. R. Paludan, Microglia activate early antiviral responses upon herpes simplex virus 1 entry into the brain to counteract development of encephalitis-like disease in mice. *J. Virol.* **96**, e0131121 (2022).

24. A. Rybak-Wolf, E. Wyler, T. M. Pentimalli, I. Legnini, A. Oliveras Martinez, P. Glažar, A. Loewa, S. J. Kim, B. B. Kaufer, A. Woehler, M. Landthaler, N. Rajewsky, Modelling viral encephalitis caused by herpes simplex virus 1 infection in cerebral organoids. *Nat. Microbiol.* **8**, 1252–1266 (2023).

25. N. H. Gopee, E. Winheim, B. Olabi, C. Admane, A. R. Foster, N. Huang, R. A. Botting, F. Torabi, D. Sumanaweera, A. P. Le, J. Kim, L. Verger, E. Stephenson, D. Adão, C. Ganier, K. Y. Gim, S. A. Serdy, C. Deakin, I. Goh, L. Steele, K. Annusver, M.-U. Miah, W. M. Tun, P. Moghimi, K. A. Kwakwa, T. Li, D. Basurto Lozada, B. Rumney, C. L. Tudor, K. Roberts, N.-J. Chipampe, K. Sidhpura, J. Englebert, L. Jardine, G. Reynolds, A. Rose, V. Rowe, S. Pritchard, I. Mulas, J. Fletcher, D.-M. Popescu, E. Poyner, A. Dubois, A. Guy, A. Filby, S. Lisgo, R. A. Barker, I. A. Glass, J.-E. Park, R. Vento-Tormo, M. T. Nikolova, P. He, J. E. G. Lawrence, J. Moore, S. Ballereau, C. B. Hale, V. Shanmugiah, D. Horsfall, N. Rajan, J. A. McGrath, E. A. O'Toole, B. Treutlein, O. Bayraktar, M. Kasper, F. Progatzky, P. Mazin, J. Lee, L. Gambardella, K. R. Koehler, S. A. Teichmann, M. Haniffa, A prenatal skin atlas reveals immune regulation of human skin morphogenesis. *Nature* **635**, 679–689 (2024).

26. B. L. Trus, W. W. Newcomb, N. Cheng, G. Cardone, L. Marekov, F. L. Homa, J. C. Brown, A. C. Steven, Allosteric signaling and a nuclear exit strategy: Binding of UL25/UL17 heterodimers to DNA-filled HSV-1 capsids. *Mol. Cell* **26**, 479–489 (2007).

27. A. J. Rutkowski, F. Erhard, A. L'Hernault, T. Bonfert, M. Schilhabel, C. Crump, P. Rosenstiel, S. Efstathiou, R. Zimmer, C. C. Friedel, L. Dölken, Widespread disruption of host transcription termination in HSV-1 infection. *Nat. Commun.* **6**, 7126 (2015).

28. E. Wyler, J. Menegatti, V. Franke, C. Kocks, A. Boltengagen, T. Hennig, K. Theil, A. Rutkowski, C. Ferrai, L. Baer, L. Kermas, C. Friedel, N. Rajewsky, A. Akalin, L. Dölken, F. Grässer, M. Landthaler, Widespread activation of antisense transcription of the host genome during herpes simplex virus 1 infection. *Genome Biol.* **18**, 209 (2017).

29. F. Full, M. van Gent, K. M. J. Sparrer, C. Chiang, M. A. Zurenski, M. Scherer, N. H. Brockmeyer, L. Heinzerling, M. Stürzl, K. Korn, T. Stamminger, A. Ensser, M. U. Gack, Centrosomal protein TRIM43 restricts herpesvirus infection by regulating nuclear lamina integrity. *Nat. Microbiol.* **4**, 164–176 (2019).

30. E. Wyler, V. Franke, J. Menegatti, C. Kocks, A. Boltengagen, S. Praktiknjo, B. Walch-Rückheim, J. Bosse, N. Rajewsky, F. Grässer, A. Akalin, M. Landthaler, Single-cell RNA-sequencing of herpes simplex virus 1-infected cells connects NRF2 activation to an antiviral program. *Nat. Commun.* **10**, 4878 (2019).

31. F. Full, S. Walter, E. Neugebauer, J. Tan, N. Drayman, V. Franke, S. Tay, M. Landthaler, A. Akalin, A. Ensser, E. Wyler, Herpesviruses mimic zygotic genome activation to promote viral replication. *Res Sq*. (2023).

32. E. Neugebauer, S. Walter, J. Tan, N. Drayman, V. Franke, M. van Gent, S. Pennisi, P. Veratti, K. S. Stein, I. Welker, S. Tay, G. M. G. M. Verjans, H. T. M. Timmers, A. Akalin, M. Landthaler, A. Ensser, E. Wyler, F. Full, Herpesviruses mimic zygotic genome activation to promote viral replication. *Nat. Commun.* **16**, 710 (2025).

33. F. Uhlitz, A. Sieber, E. Wyler, R. Fritsche-Guenther, J. Meisig, M. Landthaler, B. Klinger, N. Blüthgen, An immediate-late gene expression module decodes ERK signal duration. *Mol. Syst. Biol.* **13**, 928 (2017).

34. W. L. Hsu, H. A. Saffran, J. R. Smiley, Herpes simplex virus infection stabilizes cellular IEX-1 mRNA. *J. Virol.* **79**, 4090–4098 (2005).

35. B. Taddeo, A. Esclatine, W. Zhang, B. Roizman, The stress-inducible immediate-early responsive gene IEX-1 is activated in cells infected with herpes simplex virus 1, but several viral mechanisms, including 3′ degradation of its RNA, preclude expression of the gene. *J. Virol.* **77**, 6178–6187 (2003).

36. J. A. Corcoran, W. L. Hsu, J. R. Smiley, Herpes simplex virus ICP27 is required for virus-induced stabilization of the ARE-containing IEX-1 mRNA encoded by the human IER3 gene. *J. Virol.* **80**, 9720–9729 (2006).

37. O. Haller, G. Kochs, Mx genes: Host determinants controlling influenza virus infection and trans-species transmission. *Hum. Genet.* **139**, 695–705 (2020).

38. S. Chopra, L. M. Roesner, K. Döhner, J. Zeitvogel, S. Traidl, E. Rodriguez, I. Harder, W. Lieb, S. Weidinger, T. F. Schulz, B. Sodeik, T. Werfel, Identification and characterization of collagen XXIII alpha 1 as a novel risk factor for eczema herpeticum. medRxiv 24310236 [Preprint] (2024). https://doi.org/10.1101/2024.07.13.24310236.

39. K. Jones, S. Ramirez-Perez, S. Niu, U. Gangishetti, H. Drissi, P. Bhattaram, SOX4 and RELA function as transcriptional partners to regulate the expression of TNF-responsive genes in fibroblast-like synoviocytes. *Front. Immunol.* **13**, 789349 (2022).

40. H. Rana, N. R. Truong, D. R. Sirimanne, A. L. Cunningham, Breaching the barrier: Investigating initial herpes simplex viral infection and spread in human skin and mucosa. *Viruses* **16**, 1790 (2024).

41. T. Richardo, X. Liu, K. Döhner, T.-Y. Chao, A. Buch, A. Binz, A. Pohlmann, M. de le Roi, W. Baumgärtner, K. Brand, R. Bauerfeind, R. Förster, B. Sodeik, S. Halle, Herpes simplex virus

assembly and spread in murine skin after infection from the outside. *J. Virol.* **99**, e0163824 (2025).

42. P. Petermann, K. Thier, E. Rahn, F. J. Rixon, W. Bloch, S. Özcelik, C. Krummenacher, M. J. Barron, M. J. Dixon, S. Scheu, K. Pfeffer, D. Knebel-Mörsdorf, Entry mechanisms of herpes simplex virus 1 into murine epidermis: Involvement of nectin-1 and herpesvirus entry mediator as cellular receptors. *J. Virol.* **89**, 262–274 (2015).

43. L. Bai, J. Xu, L. Zeng, L. Zhang, F. Zhou, A review of HSV pathogenesis, vaccine development, and advanced applications. *Mol. Biomed.* **5**, 35 (2024).

44. D. Gopinath, K. H. Koe, M. K. Maharajan, S. Panda, A comprehensive overview of epidemiology, pathogenesis and the management of herpes labialis. *Viruses* **15**, 225 (2023).

45. C. Barreau, L. Paillard, H. B. Osborne, AU-rich elements and associated factors: Are there unifying principles? *Nucleic Acids Res.* **33**, 7138–7150 (2005).

46. J. L. E. Dean, G. Sully, A. R. Clark, J. Saklatvala, The involvement of AU-rich element-binding proteins in p38 mitogen-activated protein kinase pathway-mediated mRNA stabilisation. *Cell. Signal.* **16**, 1113–1121 (2004).

47. A. Arlt, H. Schäfer, Role of the immediate early response 3 (IER3) gene in cellular stress response, inflammation and tumorigenesis. *Eur. J. Cell Biol.* **90**, 545–552 (2011).

48. M. X. Wu, Z. Ao, K. V. Prasad, R. Wu, S. F. Schlossman, IEX-1L, an apoptosis inhibitor involved in NF-κB-mediated cell survival. *Science* **281**, 998–1001 (1998).

49. O. E. Akilov, M. X. Wu, I. V. Ustyugova, L. D. Falo Jr., L. J. Geskin, Resistance of Sézary cells to TNF-α-induced apoptosis is mediated in part by a loss of TNFR1 and a high level of the IER3 expression. *Exp. Dermatol.* **21**, 287–292 (2012).

50. J. Oyler-Yaniv, A. Oyler-Yaniv, E. Maltz, R. Wollman, TNF controls a speed-accuracy tradeoff in the cell death decision to restrict viral spread. *Nat. Commun.* **12**, 2992 (2021).

51. T. Recaldin, L. Steinacher, B. Gjeta, M. F. Harter, L. Adam, K. Kromer, M. P. Mendes, M. Bellavista, M. Nikolaev, G. Lazzaroni, R. Krese, U. Kilik, D. Popovic, B. Stoll, R. Gerard, M. Bscheider, M. Bickle, L. Cabon, J. G. Camp, N. Gjorevski, Human organoids with an autologous tissue-resident immune compartment. *Nature* **633**, 165–173 (2024).

52. W. Hafezi, E. U. Lorentzen, B. R. Eing, M. Müller, N. J. King, B. Klupp, T. C. Mettenleiter, J. E. Kühn, Entry of herpes simplex virus type 1 (HSV-1) into the distal axons of trigeminal neurons favors the onset of nonproductive, silent infection. *PLOS Pathog.* **8**, e1002679 (2012).

53. T. Köller, D. Kurze, M. Lange, M. Scherdin, A. Podbielski, P. Warnke, Implementation and evaluation of a fully automated multiplex real-time PCR assay on the BD max platform to detect and differentiate herpesviridae from cerebrospinal fluids. *PLOS ONE* **11**, e0153991 (2016).

54. M. Weidmann, U. Meyer-König, F. T. Hufert, Rapid detection of herpes simplex virus and varicella-zoster virus infections by real-time PCR. *J. Clin. Microbiol.* **41**, 1565–1568 (2003).

55. P. A. Ewels, A. Peltzer, S. Fillinger, H. Patel, J. Alneberg, A. Wilm, M. U. Garcia, P. Di Tommaso, S. Nahnsen, The nf-core framework for community-curated bioinformatics pipelines. *Nat. Biotechnol.* **38**, 276–278 (2020).

56. P. Di Tommaso, M. Chatzou, E. W. Floden, P. P. Barja, E. Palumbo, C. Notredame, Nextflow enables reproducible computational workflows. *Nat. Biotechnol.* **35**, 316–319 (2017).

57. A. Dobin, C. A. Davis, F. Schlesinger, J. Drenkow, C. Zaleski, S. Jha, P. Batut, M. Chaisson, T. R. Gingeras, STAR: Ultrafast universal RNA-seq aligner. *Bioinformatics* **29**, 15–21 (2013).

58. R. Patro, G. Duggal, M. I. Love, R. A. Irizarry, C. Kingsford, Salmon provides fast and bias-aware quantification of transcript expression. *Nat. Methods* **14**, 417–419 (2017).

59. C. Soneson, M. Love, M. Robinson, Differential analyses for RNA-seq: Transcript-level estimates improve gene-level inferences. *F1000Res.* **4**, 1521 (2015).

60. M. I. Love, W. Huber, S. Anders, Moderated estimation of fold change and dispersion for RNA-seq data with DESeq2. *Genome Biol.* **15**, 550 (2014).

61. J. M. Elizarraras, Y. Liao, Z. Shi, Q. Zhu, A. R. Pico, B. Zhang, WebGestalt 2024: Faster gene set analysis and new support for metabolomics and multi-omics. *Nucleic Acids Res.* **52**, W415–W421 (2024).

62. D. Kim, J. M. Paggi, C. Park, C. Bennett, S. L. Salzberg, Graph-based genome alignment and genotyping with HISAT2 and HISAT-genotype. *Nat. Biotechnol.* **37**, 907–915 (2019).

63. Y. Liao, G. K. Smyth, W. Shi, featureCounts: An efficient general purpose program for assigning sequence reads to genomic features. *Bioinformatics* **30**, 923–930 (2014).

64. A. Manukyan, E. Bahry, E. Wyler, E. Becher, A. Pascual-Reguant, I. Plumbom, H. O. Dikmen, S. Elezkurtaj, T. Conrad, J. Altmüller, A. E. Hauser, A. Hocke, H. Radbruch, D. Schmidt, M. Landthaler, A. Akalin, VoltRon: A spatial omics analysis platform for multi-resolution and multi-omics integration using image registration. bioRxiv 571667 [Preprint] (2023). https://doi.org/10.1101/2023.12.15.571667.

65. H. Wickham, M. Averick, J. Bryan, W. Chang, L. D. McGowan, R. François, G. Grolemund, A. Hayes, L. Henry, J. Hester, M. Kuhn, T. L. Pedersen, E. Miller, S. M. Bache, K. Müller, J. Ooms, D. Robinson, D. P. Seidel, V. Spinu, K. Takahashi, D. Vaughan, C. Wilke, K. Woo, H. Yutani, Welcome to the Tidyverse. *J. Open Source Softw.* **4**, 1686 (2019).

66. A. Zhu, J. G. Ibrahim, M. I. Love, Heavy-tailed prior distributions for sequence count data: Removing the noise and preserving large differences. *Bioinformatics* **35**, 2084–2092 (2019).

67. E. Pebesma, Simple features for R: Standardized support for spatial vector data. *R J.* **10**, 439–446 (2018).

68. J. Gombin, R. Vaidyanathan, V. Agafonkin, concaveman: A very fast 2D concave hull algorithm (2020).

69. M. Kay, L. Elkin, J. J. Higgins, J. O. Wobbrock, ARTool: Aligned rank transform for nonparametric factorial ANOVAs. R package version 0.11.2 (2025).

70. D. Bates, M. Mächler, B. Bolker, S. Walker, Fitting linear mixed-effects models using lme4. *J. Stat. Softw.* **67**, 1–48 (2015).

71. A. Kuznetsova, P. B. Brockhoff, R. H. B. Christensen, lmerTest package: Tests in linear mixed effects models. *J. Stat. Softw.* **82**, 1–26 (2017).

72. S. Albertini, M. Czech-Sioli, T. Günther, S. Virdi, P. Blümke, L. Röpke, V. Brinschwitz, M. Dandri, L. Allweiss, R. Reimer, C. Schneider, A. Hansen, S. Krasemann, E. Wyler, M. Landthaler, A. Grundhoff, N. Fischer, Merkel cell polyomavirus infection and persistence modelled in skin organoids. bioRxiv 637697 [Preprint] (2025). https://doi.org/10.1101/2025.02.11.637697.

73. Y. Hao, T. Stuart, M. H. Kowalski, S. Choudhary, P. Hoffman, A. Hartman, A. Srivastava, G. Molla, S. Madad, C. Fernandez-Granda, R. Satija, Dictionary learning for integrative, multimodal and scalable single-cell analysis. *Nat. Biotechnol.* **42**, 293–304 (2024).

74. I. Korsunsky, N. Millard, J. Fan, K. Slowikowski, F. Zhang, K. Wei, Y. Baglaenko, M. Brenner, P. R. Loh, S. Raychaudhuri, Fast, sensitive and accurate integration of single-cell data with Harmony. *Nat. Methods* **16**, 1289–1296 (2019).

75. C. Hafemeister, R. Satija, Normalization and variance stabilization of single-cell RNA-seq data using regularized negative binomial regression. *Genome Biol.* **20**, 296 (2019).

76. V. Singhal, N. Chou, J. Lee, Y. Yue, J. Liu, W. K. Chock, L. Lin, Y. C. Chang, E. M. L. Teo, J. Aow, H. K. Lee, K. H. Chen, S. Prabhakar, BANKSY unifies cell typing and tissue domain segmentation for scalable spatial omics data analysis. *Nat. Genet.* **56**, 431–441 (2024).
